# Supplementary material for: Sharing the effort of the European Green Deal among countries
Source: Nat Commun. 2022 Jun 27;13:3673. doi: 10.1038/s41467-022-31204-8 (PMC9237065; doi:10.1038/s41467-022-31204-8)
Supplement: Supplementary file 1 — Supplementary Information [file 41467_2022_31204_MOESM1_ESM.pdf]

Supplementary Information – Sharing the effort of the European Green Deal among countries

Karl W. Steininger[1,2], Keith Williges[1,3], Lukas H. Meyer[3], Florian Maczek[4], and Keywan Riahi[4]

[1] University of Graz, Wegener Center for Climate and Global Change, Brandhofgasse 5, A-8010 Graz, Austria
[2] University of Graz, Department of Economics, Universitaetsstr. 15, A-8010 Graz, Austria
[3] University of Graz, Institute of Philosophy, Attemsgasse 25, A-8010 Graz, Austria
[4] International Institute for Applied Systems Analysis, Energy, Climate and Environment Program, Schlossplatz 1, A-2361 Laxenburg, Austria

1. The Triptych Approach

In 1996, in preparation for its EU presidency and in order to resolve questions concerning the individual contribution of Member States to the aggregate EU climate target and the associated economic burden, the Netherlands' government commissioned a study on burden differentiation within the EU which resulted in the so-called Triptych sectoral approach[1]. This approach differentiates three economic segments, the power sector, the internationally operating energy-intensive industry and the remaining domestically oriented sectors. Emission reductions have been calculated by applying rules, such as limitation of coal use of power production, minimum requirements for renewable energy, and minimum energy efficiency improvement rates in the industry. For the domestic sectors a per capita emission allowance approach was used. The application of the Triptych approach is considered to have been successful in this initial political (negotiation) process of the EU and its Member States, because "it resulted in increased insight among EU negotiators concerning differences in national circumstances and their role in greenhouse gas emissions"[2]. Sijm et al. [2] conclude "The Triptych approach was accepted by the EU and its Member States because it was based on the main issues encountered in the negotiations: differences in population size, in standard of living, in fuel mix, in economic structure and the competitiveness of internationally oriented industries. It seems that the incorporation of different equity principles in the Triptych approach was successful for the political acceptability of the burden sharing agreement." Building upon that experience, the regulatory effort sharing framework to cover emissions outside the EU wide Emission Trading System was further developed, with the Effort Sharing Decision and its successor, the Effort Sharing Regulation, adopted in 2009 and 2018, respectively.

2. Alternative governance indicators to reflect capability

As described in section "Equity principles" in the main text, the capability of states to implement measures (see [3]) for the proposed carbon emission reductions is considered to be reflected by indicators beyond economic ones such as GDP. To incorporate governance capability, the following established indicators (see overviews by Savoia and Sen [4] and Hanson and Sigman [5]) have been considered and assessed with our effort sharing algorithm to identify further alternatives in the capability dimension. Supplementary Figure 1 plots the results of these indicators used in a *C3-Governance* interpretation to distribute 2030 emissions. Given that the three indicators specifically developed to assess state capacity (government effectiveness index, quality of government index, and state capacity) all result in similar allocations, minus occasional outliers, and that all three cover wider aspects of state capacity than that of GDP (or derivations based on it), just one of them, the indicator gee_n (government effectiveness), as showing largest impact divergence from GDP, was chosen to be used in the *C3-Governance* interpretation in the main text.

Logarithm of GDP:

A logarithmic version of GDP per capita. It has been used as a proxy for financial, administrative, police, and military capabilities of a state [6] and by the World Bank Group. For the effort sharing algorithm the data of 2020 was used.

Government effectiveness index (gee/gee_n):

The government effectiveness index is an index from the World Bank Group including 193 countries [7]. It uses 47 variables and its value ranges from -2,5 to 2,5. It is also available in a normalized version, that ranges from 0 to 1. Since the effort sharing algorithm is not compatible with negative number input, only the normalized version (gee_n) was used in the analysis. For the effort sharing algorithm the data of 2020 was used.

The Quality of Government Index:

The Quality of Government (QoG) standard data is a database maintained by the Quality of Government (QoG) Institute at the University of Gothenburg [8]. The primary index used here represents the mean value of three variables from the „International Country Risk Guide“ from “The PRS Group”. The three variables are: Corruption, Law and Order and Bureaucracy Quality. The index (Icqr_qog) is available from 1984 to present. For the effort sharing algorithm the data of 2020 was used.

State Capacity:

The state capacity indicator from Hanson and Sigman [5] is created from several measures that are conceptually linked to three dimensions of state capacity: extractive capacity, coercive capacity, and administrative capacity. The indicator covers the years from 1960 to 2015. For the effort sharing algorithm, the most recent year (2015) was used. The indicator was available for all EU member states except for Malta and Luxembourg. Therefore, the two countries are excluded in the results of the effort sharing algorithm for this indicator.

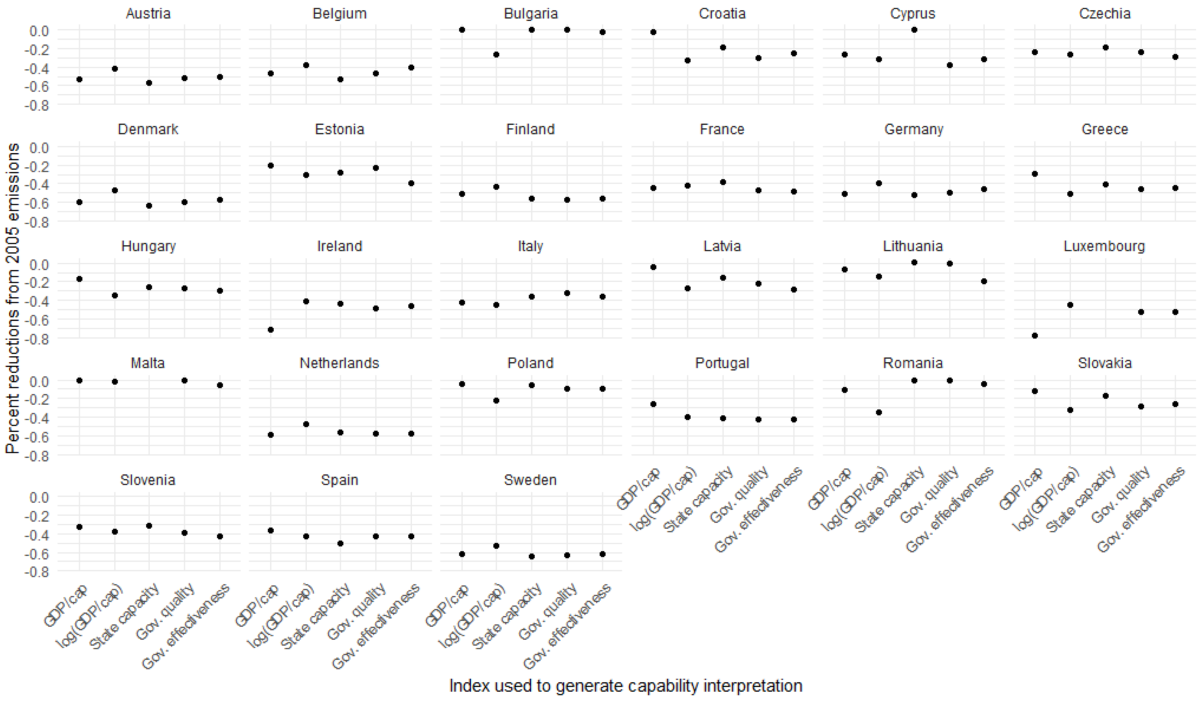

Supplementary Figure 1. Results of a 100% weighted *C3-Governenace* interpretation informed by the indicators listed on the x-axis, in percent 2030 target reductions (relative to 2005 emissions levels).

3. Sensitivity of emissions allowances to changing year of historical accounting

Discussed in the "Conclusions" section of the main text, a range of possible years could be seen as valid in discussions of establishing a historic accounting period for emissions. However, a sensitivity analysis using the *R1-hist-emi* interpretation, but with varying years at which accounting begins, finds that within a range of years 1990 to 2000, the choice of year makes little difference to overall results. Supplementary Figure 2 indicates the percentage reduction in emission required by 2030 for countries which would see a change if the historic accounting year were to change, when giving full weight to a historical emissions interpretation, and changing the year of accounting as indicated on the x-axis.

As shown, only 8 countries would notice any change, under the conditions in which our algorithm is implemented. Importantly, we assume countries cannot be burdened with negative emissions, nor do we assume allowances for emissions to rise by 2030 over their 2005 level. All countries missing from Supplementary Figure 2 would either be allocated zero emissions (i.e. 100% reductions) or those equal to 2005 emissions, due to the vast historical imbalances in emissions, and this does not change when the year of historic reference is shifted across the range 1990-2000. For all countries experiencing a change when the reference year is shifted, Supplementary Figure 2 illustrates that for all but a few countries, that change is only within a few percentage points; only Cyprus, Czechia, France and Greece would see changes of over 10% if the historic accounting period were changed across a full decade, from 1990 to 2000.

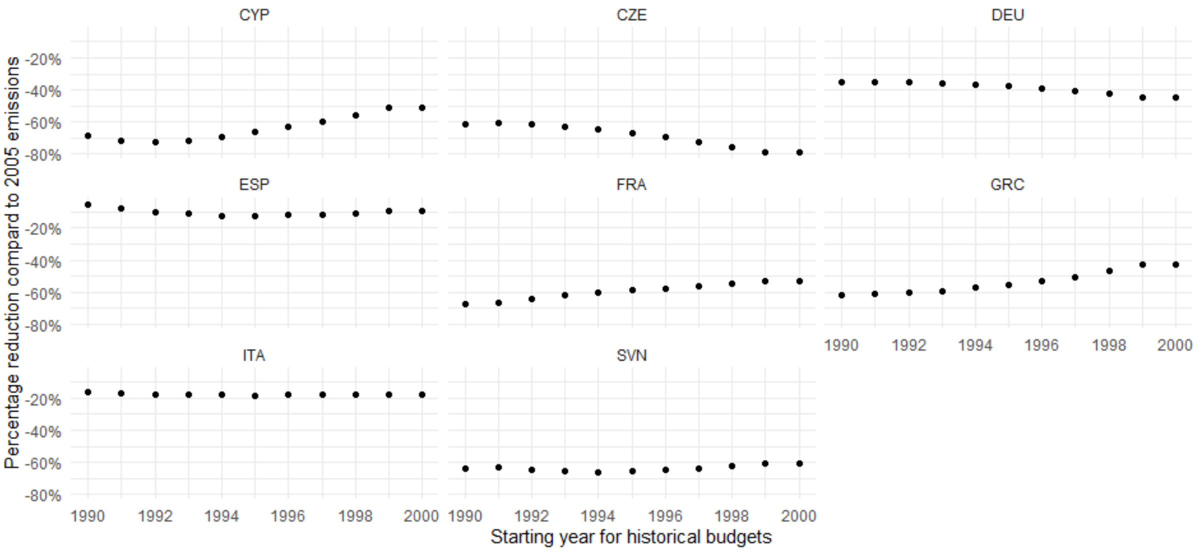

Supplementary Figure 2. 2030 emissions (in terms of reductions from 2005 levels) for countries that would experience a change under a *R1-hist-emi* interpretation if the year at which accounting begins changes (shown on the x-axis). For all other EU countries, no change is discernible as a result of changing the starting year.

4. Use of a renewable shares interpretation to address capability or responsibility concerns

Also discussed in the "Conclusions" section of the main text is the choice of Member States' renewables capacity as an indicator of either responsibility or capability equity concerns. In Supplementary Figure 3 we compare the different distribution of emissions burden – in terms of reductions compared to 2005 emissions – which emerge from using RES growth from 2005 to 2019 as either a capability (orange bar in Supplementary Figure 3) or responsibility

interpretation (black). The results shown can vary widely. We construct the capability and responsibility interpretations to have opposing impacts on emission allocation, i.e. countries with highest RES change compared to the EU average would see the highest additional emission allowances under a responsibility interpretation and conversely the least under a capability interpretation. As a result, swings in allocations are highest for countries further from the average, those countries having done the most (conversely, least) to develop RES over the specified time period.

Use of RES as either a responsibility or capability interpretation can be viewed as problematic, for two main reasons. The first deals with the capability interpretation; such an approach can be viewed as rewarding previous inaction in terms of meeting climate goals, and could encourage a future lack of effort on the part of low-emitting countries. Additionally, previous success in RES implementation is not necessarily solid evidence of being able to further expand in the future, and vice versa; e.g., countries who may not have strongly developed RES since 2005 may be in a particularly good position to increase their share in the future due to a number of factors, particularly as costs of renewables continues to fall. Alternatively, a responsibility interpretation could be seen as punishing countries for previous and/or current lack of ability (e.g. capital, be it financial, human, or natural) to increase its share of RES compared to other countries.

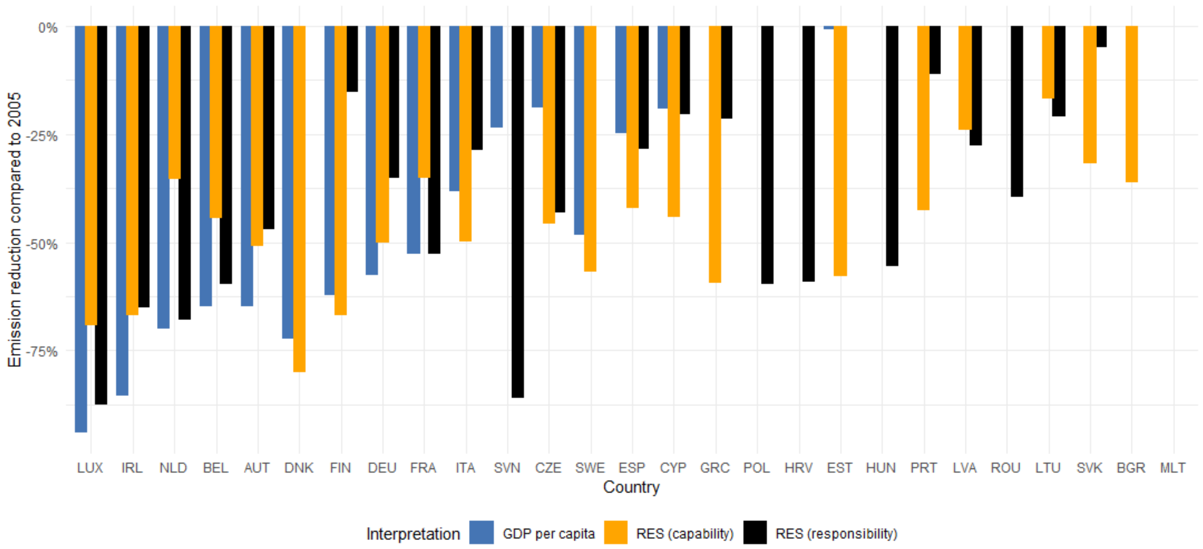

Supplementary Figure 3. Emissions reductions (in percent) by 2030 compared to 2005 emissions for all countries following a single interpretation at 100% weighting: (i) GDP per capita (C2-blue), change in renewables capacity as a capability interpretation (C4-orange), or change in renewables capacity as a responsibility interpretation (R4-black).

5. Individual country ternary chart results

This section provides individual country results, in the form of equity triangles illustrating the required emission reductions, which arises from combinations of equity interpretations, based on a fully qualified distribution approach (i.e. the weights of the capability, responsibility and equality interpretations sum to 1).

For clarity, these results can inform a country's negotiation position for the choice of budget allocation approaches. For each country, the equity triangle space depicts the emissions allowance which would result from a given combination of three qualifications. The graphs are all oriented in the same way; at the top is the C corner, for Capability, the bottom-left is R for Responsibility and bottom-right E for Equality.

The colour gradient of the ternary plot indicates the country's share of the overall EU allowance to 2030, with lighter colours indicating higher allowances. The contour lines represent constant shares as indicated. The first ternary chart displayed for each country is always the main scenario (R1, C1, E1 interpretations); this is followed by different scenarios as indicated in panel labels. Scenarios are defined as in Supplementary Table 1, which frames equity concepts along the lines of capability, responsibility and equality as discussed in IPCC framing.

Supplementary Table 1. Definition of main and alternative interpretation scenarios, and their contents.

Scenario	Capability	Responsibility	Equality
Main	EU-capability	Hist-emi	Basic-needs
C2	GDP/capita	Hist-emi	Basic-needs
C3	Governance	Hist-emi	Basic-needs
C4	RES-cap	Hist-emi	Basic-needs
E2	EU-capability	Hist-emi	ES-EPC
E3	EU-capability	Hist-emi	Full-EPC
R2	EU-capability	Benefits	Basic-needs
R3	EU-capability	C-budget	Basic-needs
R4	EU-capability	RES-expansion	Basic-needs
R5	EU-capability	Cumulative emi/cap	Basic-needs

For each scenario, we calculate a weighted combination of three interpretations (one from each equity cornerstone), with the sum of the weights of the three interpretations equal to 1. For further information on how each interpretation comprising the scenarios is generated, see *Methods* in the main text.

The page number corresponding to each country's results is listed in the table below.

**Country**	**Page number**
Austria	7
Belgium	9
Bulgaria	11
Cyprus	13
Czechia	15
Germany	17
Denmark	19
Spain	21
Estonia	23
Finland	25
France	27
Greece	29
Croatia	31
Hungary	33
Ireland	35
Italy	37
Lithuania	39
Luxembourg	41
Latvia	43
Malta	45
The Netherlands	47
Poland	49
Portugal	51
Romania	53
Slovakia	55
Slovenia	57
Sweden	59

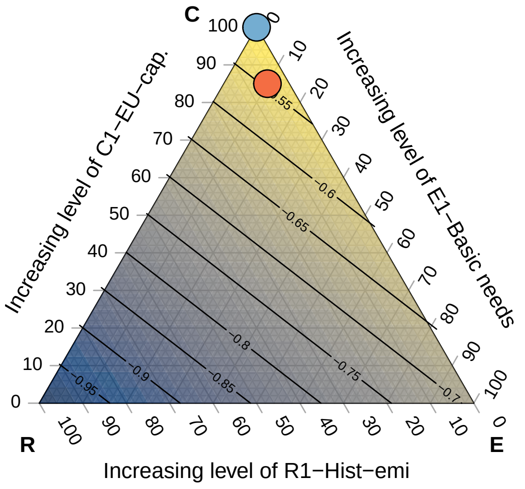

(a) Main

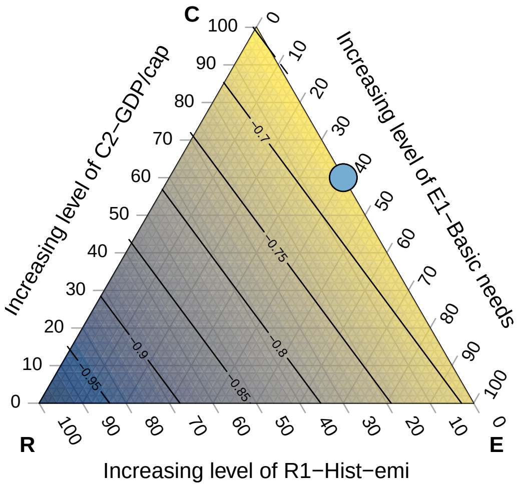

(b) C2-GDP/cap

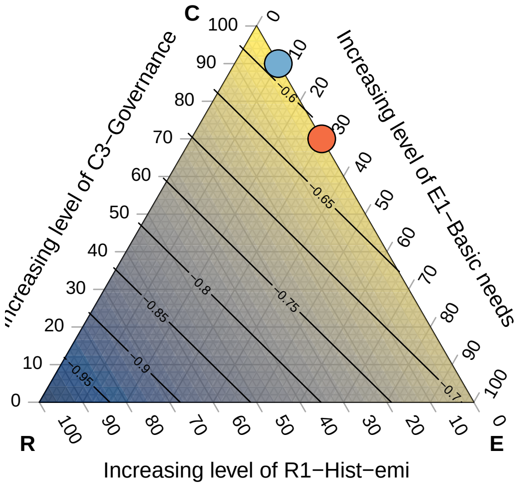

(c) C3-Governance

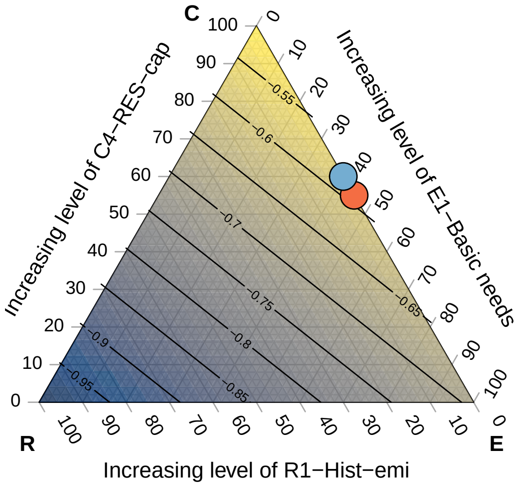

(d) C4-RES-cap.

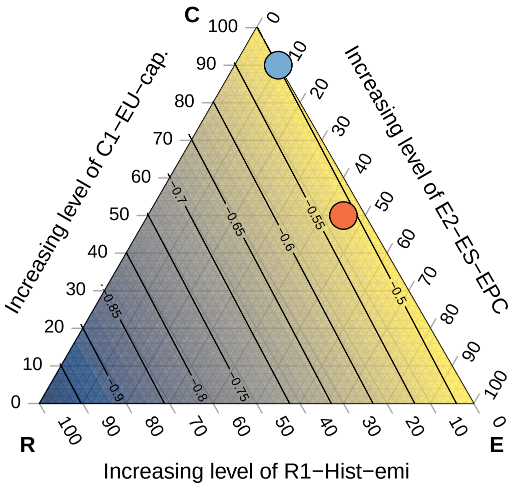

(e) E2-ES-EPC

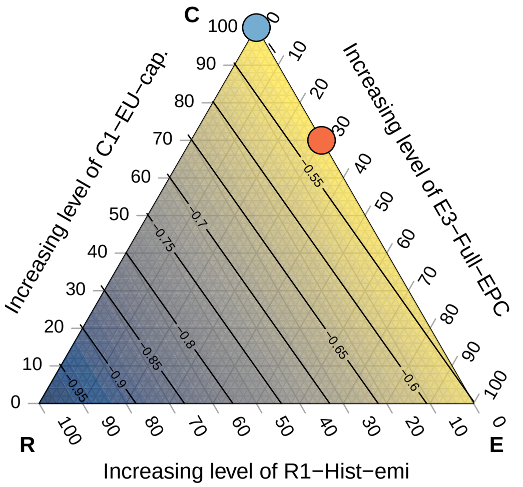

(f) E3-Full-EPC

Supplementary Figure 4. Ternary charts for Austria: combinations of various equality, responsibility and capability specifications. The current Fit for 55 target for the country is -48%.

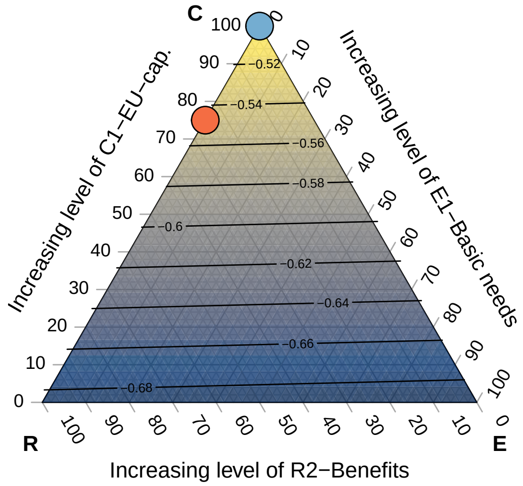

(a) R2-Benefits

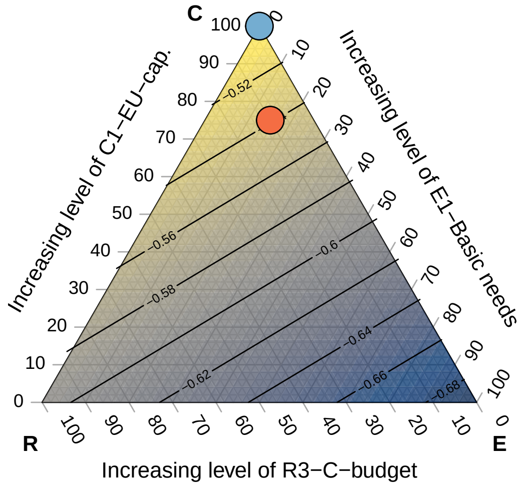

(b) R3-C-budget

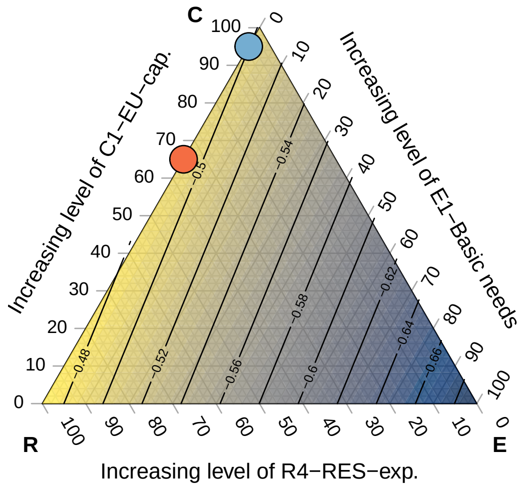

(c) R4-RES-expansion

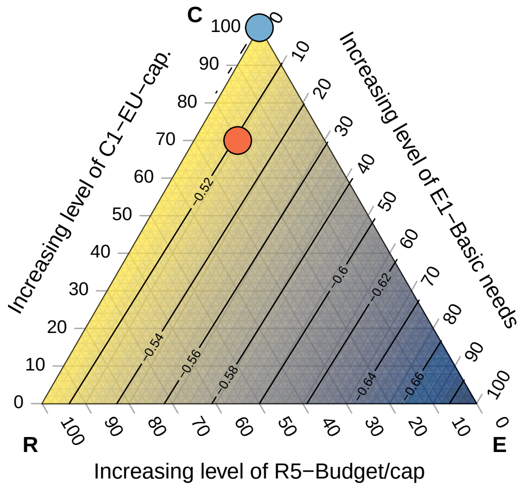

(d) R5-Budget/cap

Supplementary Figure 5. Ternary charts for Austria, cont'd

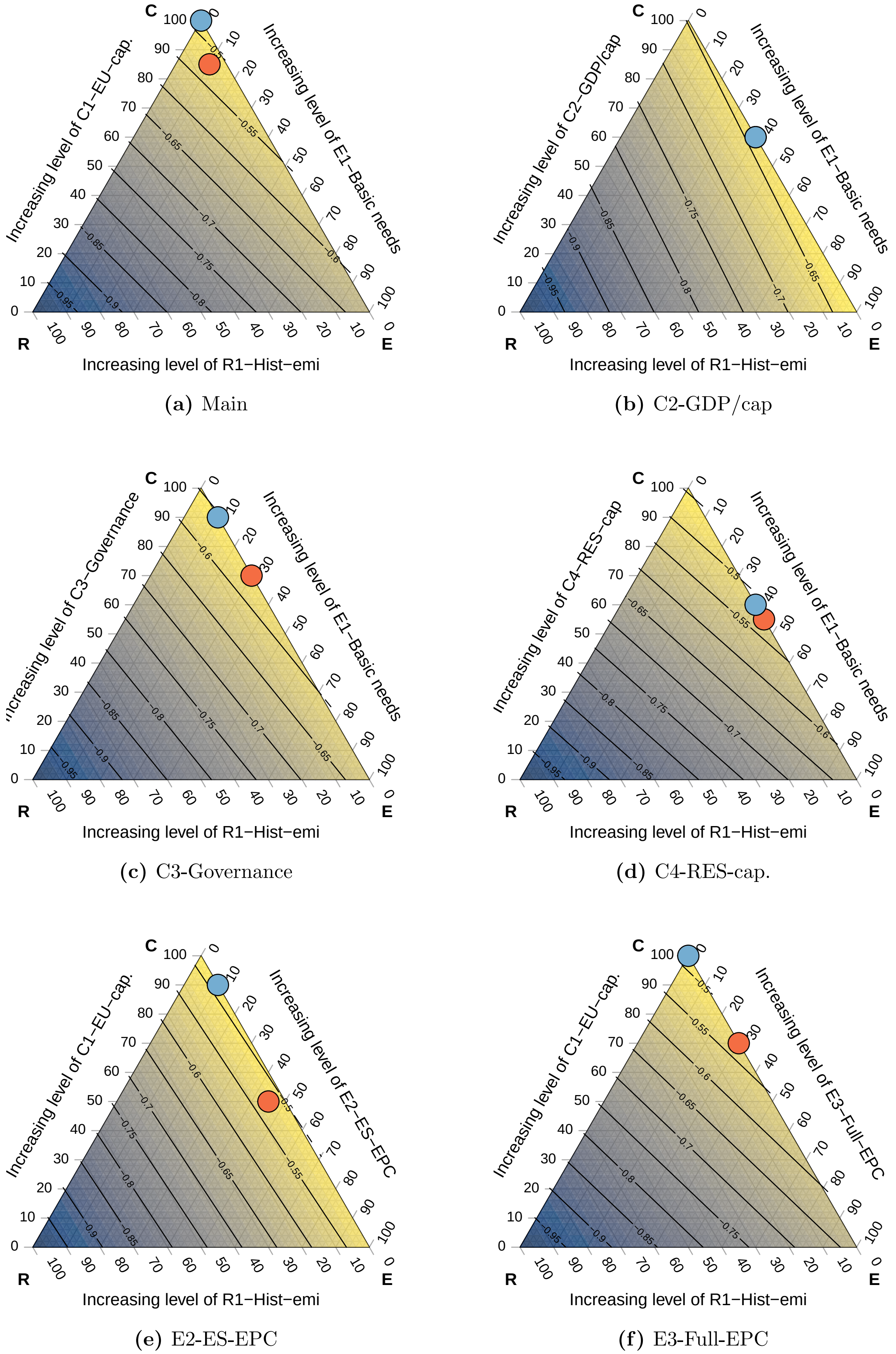

(a) Main

(b) C2-GDP/cap

(c) C3-Governance

(d) C4-RES-cap.

(e) E2-ES-EPC

(f) E3-Full-EPC

Supplementary Figure 6. Ternary charts for Belgium: combinations of various equality, responsibility and capability specifications. The current Fit for 55 target for the country is -47%.

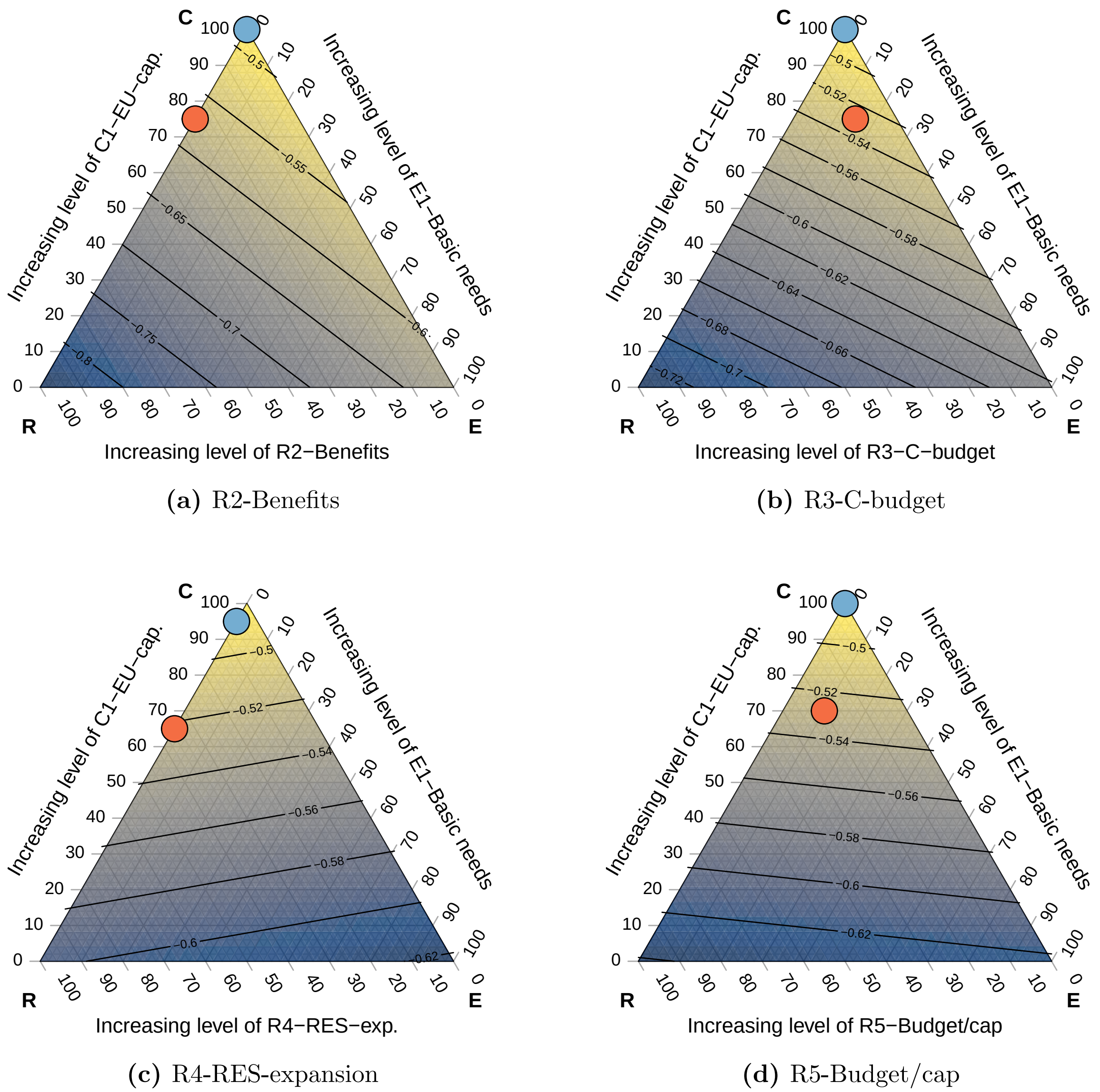

(a) R2-Benefits

(b) R3-C-budget

(c) R4-RES-expansion

(d) R5-Budget/cap

Supplementary Figure 7. Ternary charts for Belgium, cont'd

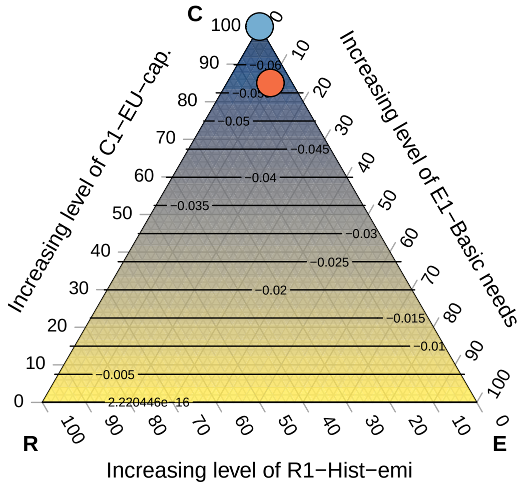

(a) Main

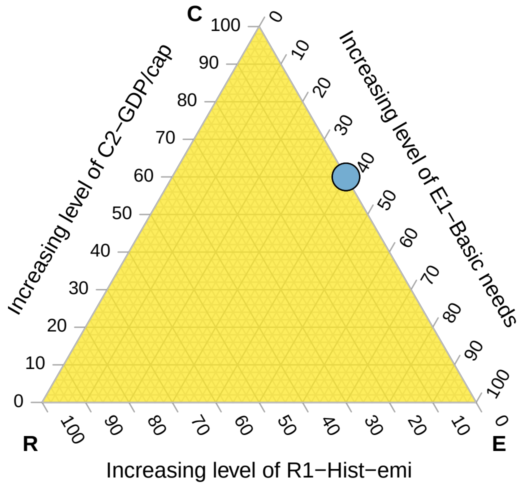

(b) C2-GDP/cap

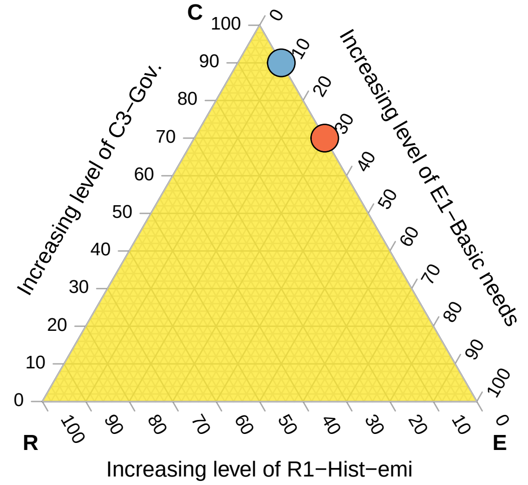

(c) C3-Governance

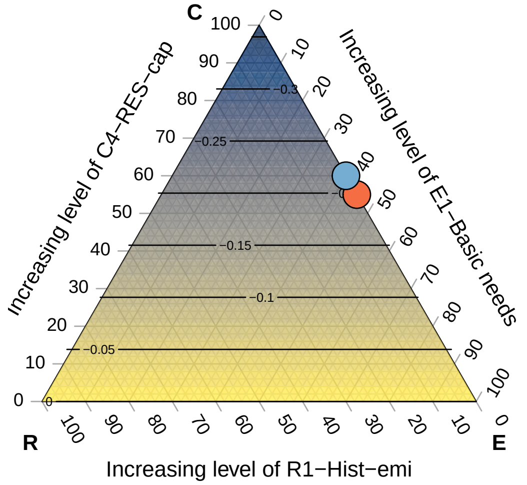

(d) C4-RES-cap.

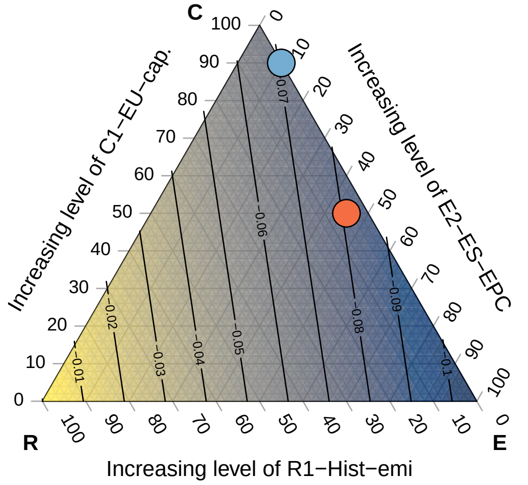

(e) E2-ES-EPC

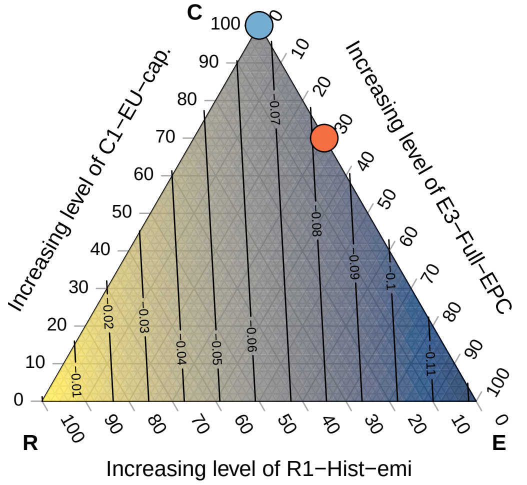

(f) E3-Full-EPC

Supplementary Figure 8. Ternary charts for Bulgaria: combinations of various equality, responsibility and capability specifications. The current Fit for 55 target for the country is -10%. For panels (b) and (c) all three interpretations result in the same reduction target.

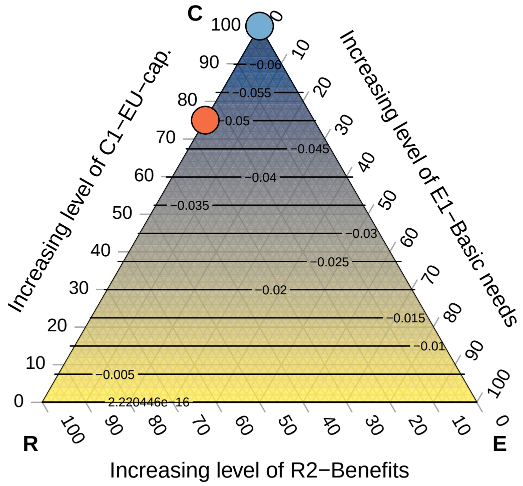

(a) R2-Benefits

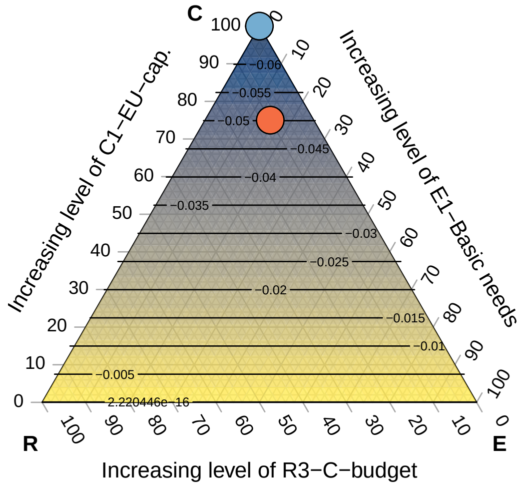

(b) R3-C-budget

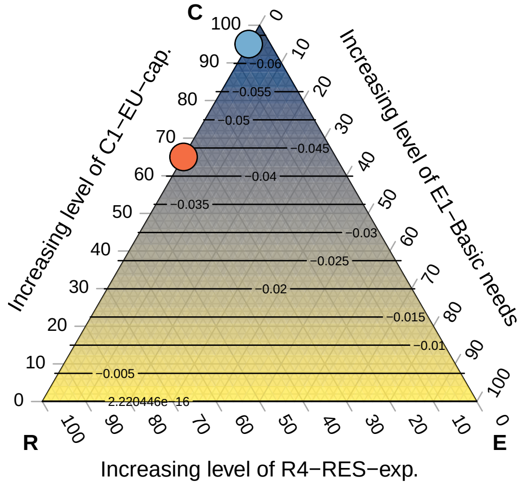

(c) R4-RES-expansion

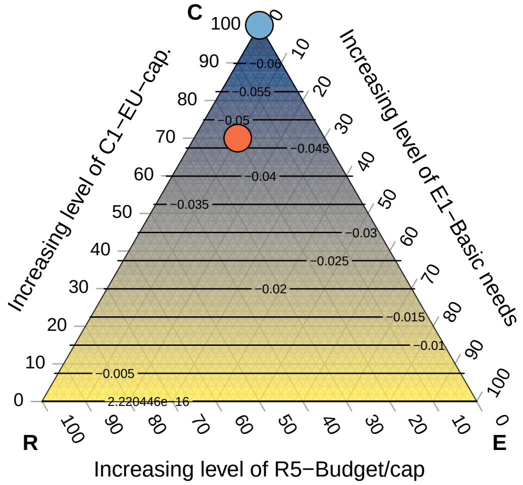

(d) R5-Budget/cap

Supplementary Figure 9. Ternary charts for Bulgaria, cont'd

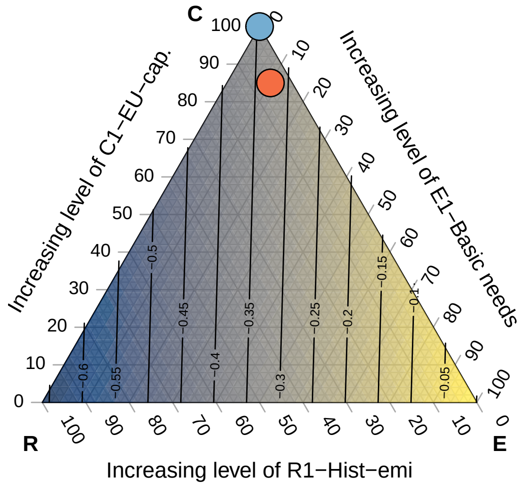

(a) Main

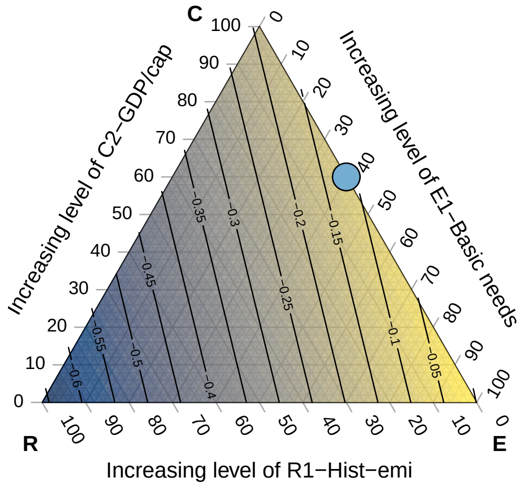

(b) C2-GDP/cap

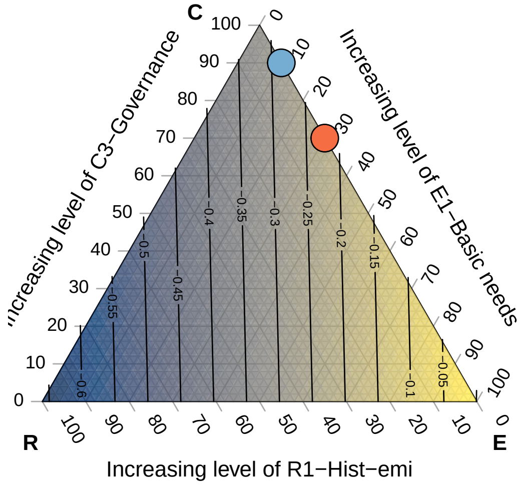

(c) C3-Governance

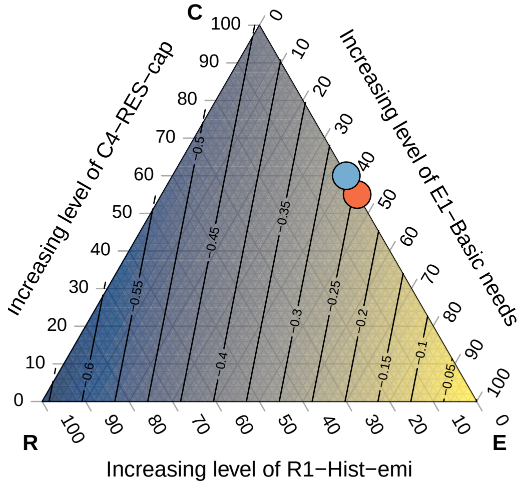

(d) C4-RES-cap.

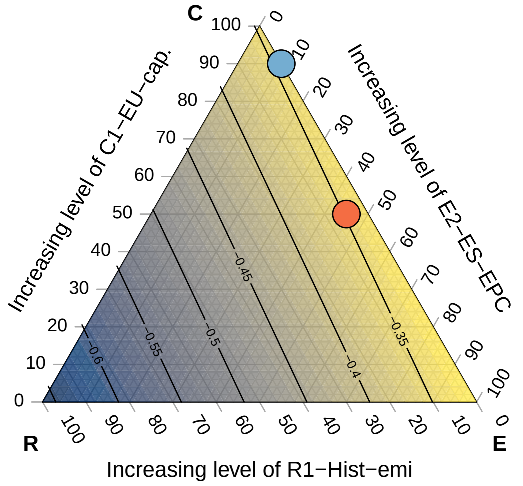

(e) E2-ES-EPC

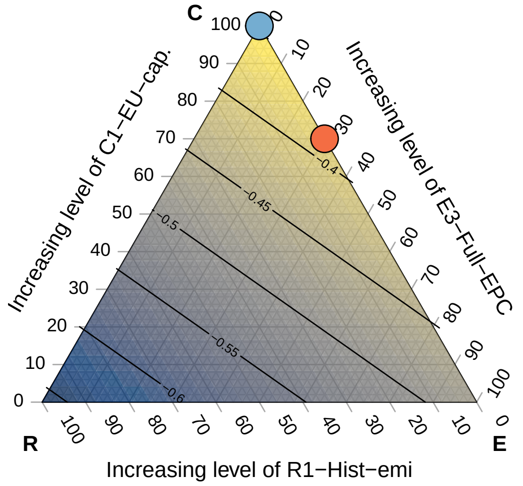

(f) E3-Full-EPC

Supplementary Figure 10. Ternary charts for Cyprus: combinations of various equality, responsibility and capability specifications. The current Fit for 55 target for the country is -32%.

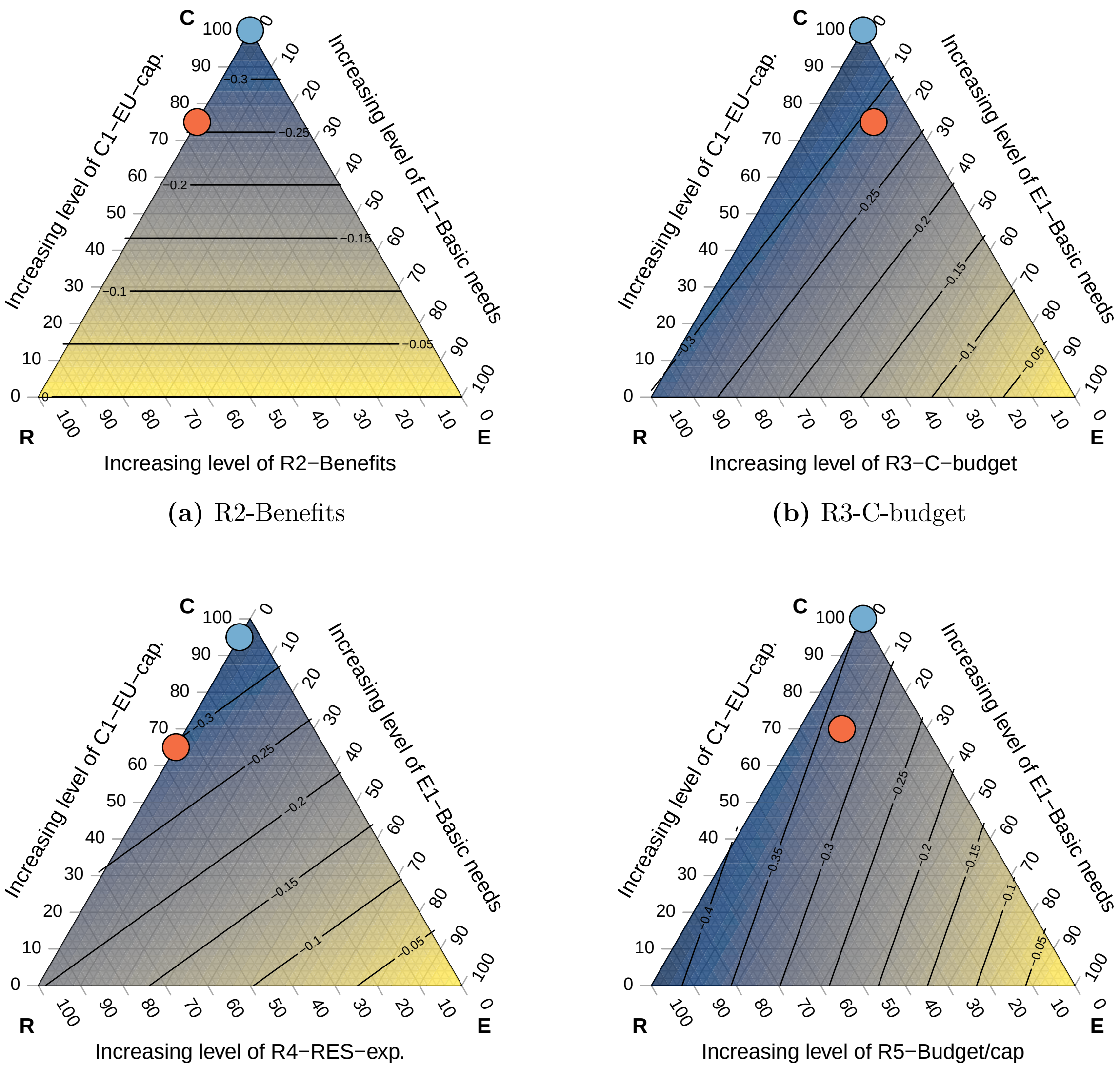

(a) R2-Benefits

(b) R3-C-budget

(c) R4-RES-expansion

(d) R5-Budget/cap

Supplementary Figure 11. Ternary charts for Cyprus, cont'd

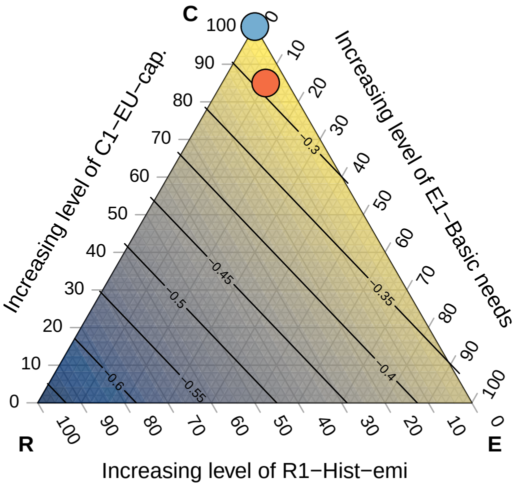

(a) Main

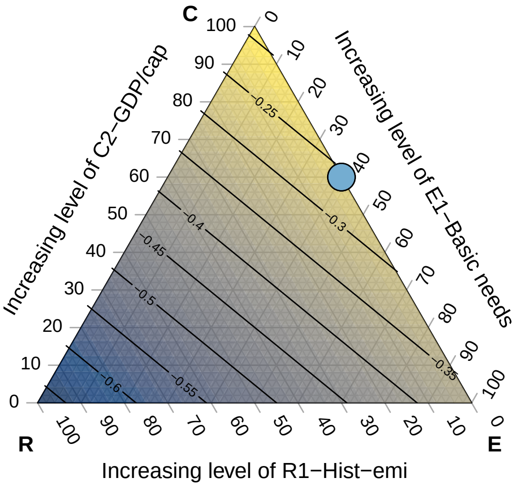

(b) C2-GDP/cap

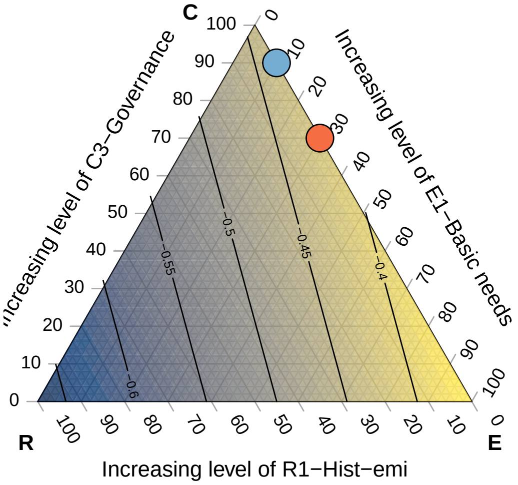

(c) C3-Governance

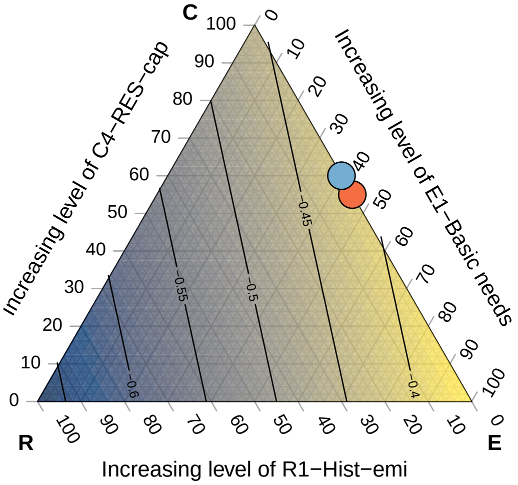

(d) C4-RES-cap.

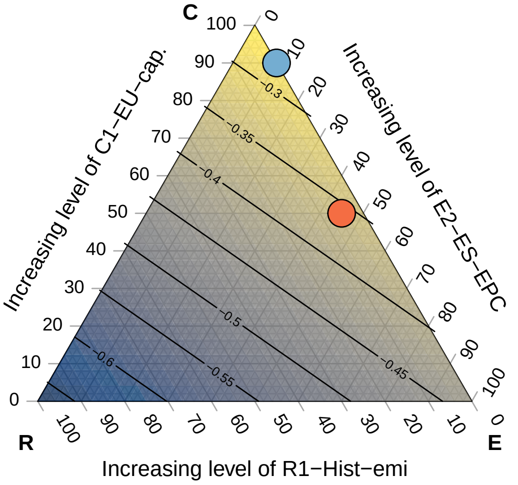

(e) E2-ES-EPC

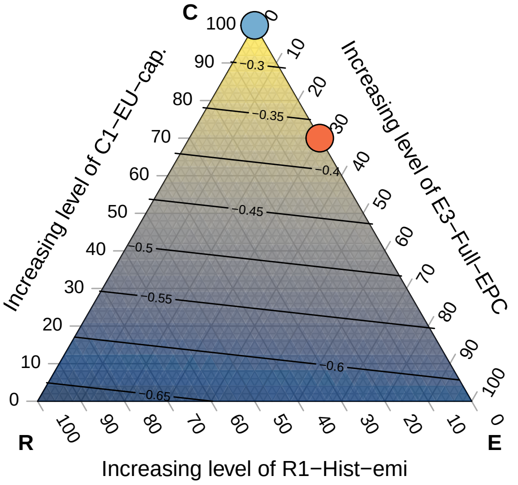

(f) E3-Full-EPC

Supplementary Figure 12. Ternary charts for Czechia: combinations of various equality, responsibility and capability specifications. The current Fit for 55 target for the country is -26%.

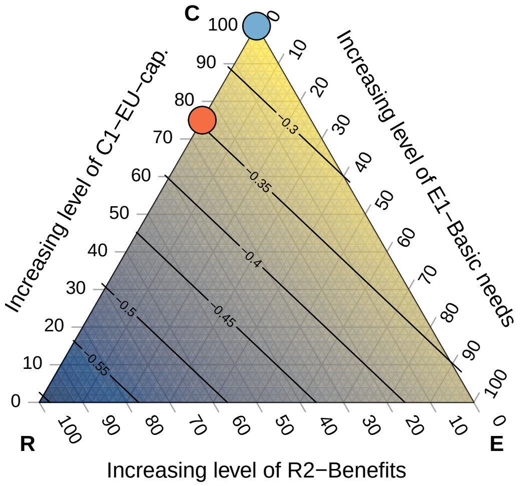

(a) R2-Benefits

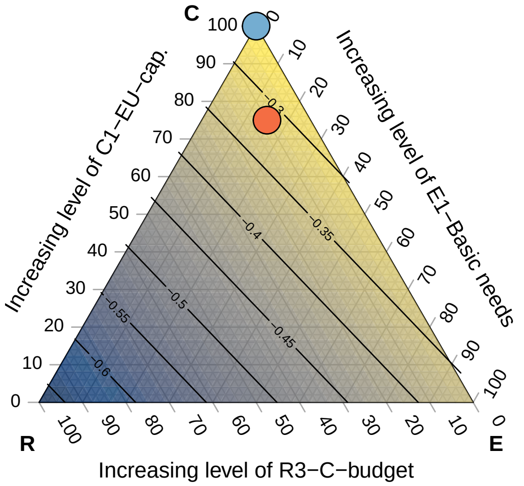

(b) R3-C-budget

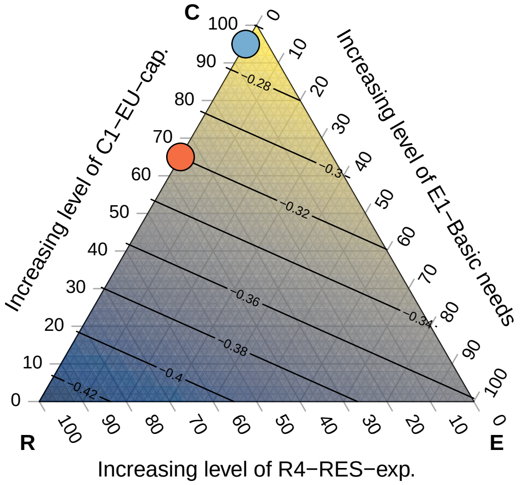

(c) R4-RES-expansion

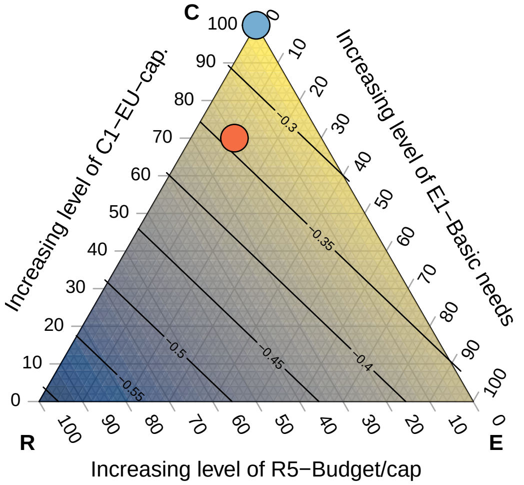

(d) R5-Budget/cap

Supplementary Figure 13. Ternary charts for Czechia, cont'd

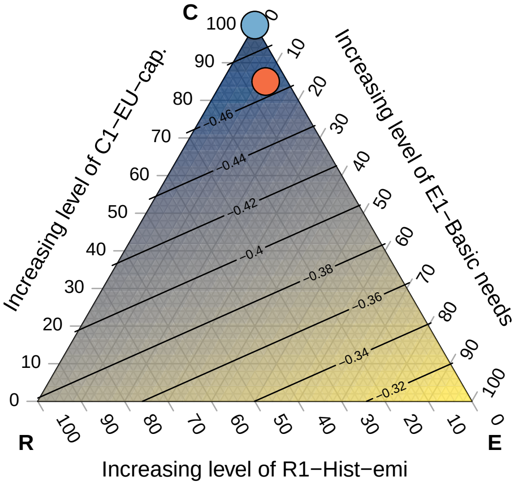

(a) Main

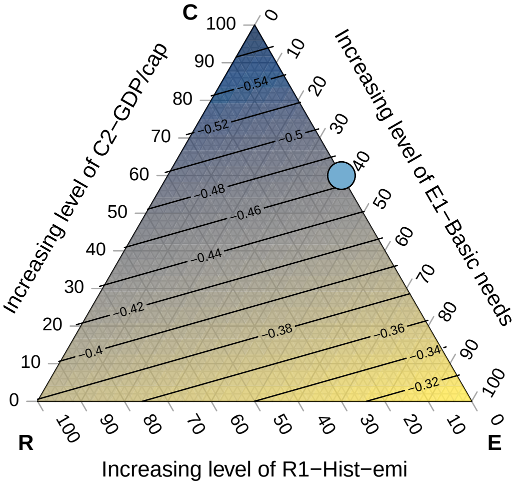

(b) C2-GDP/cap

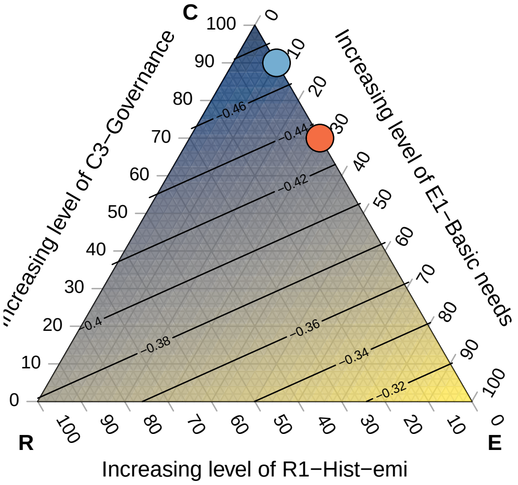

(c) C3-Governance

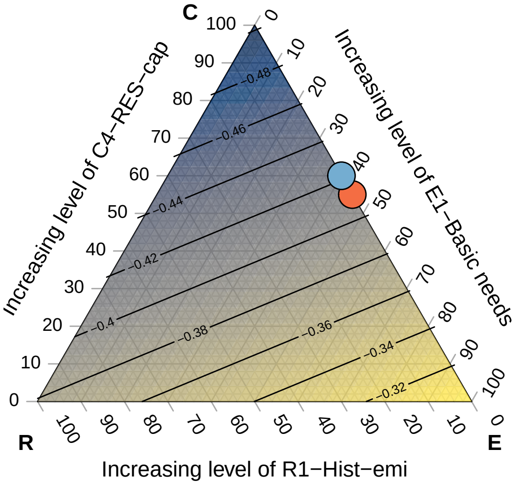

(d) C4-RES-cap.

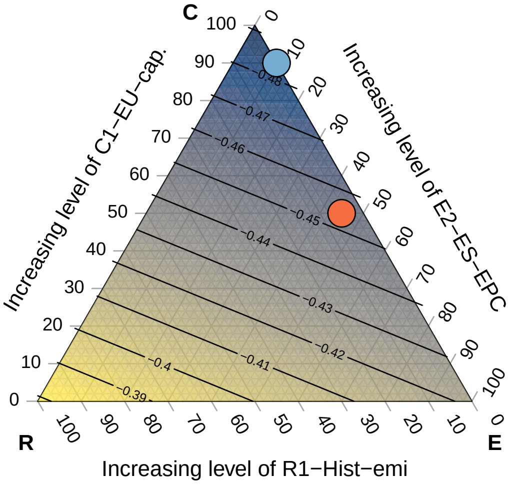

(e) E2-ES-EPC

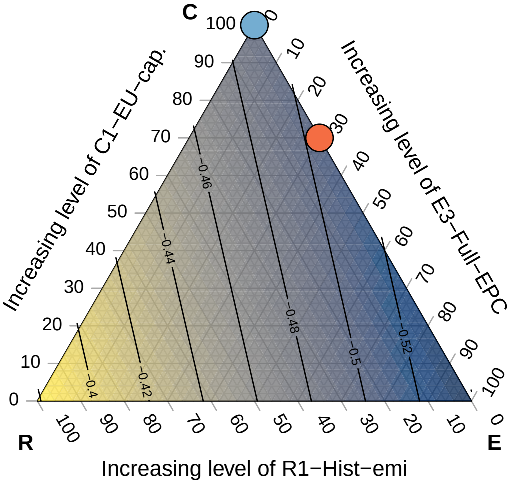

(f) E3-Full-EPC

Supplementary Figure 14. Ternary charts for Germany: combinations of various equality, responsibility and capability specifications. The current Fit for 55 target for the country is -50%.

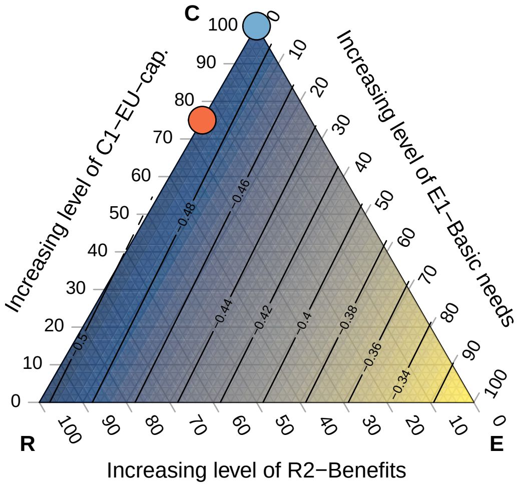

(a) R2-Benefits

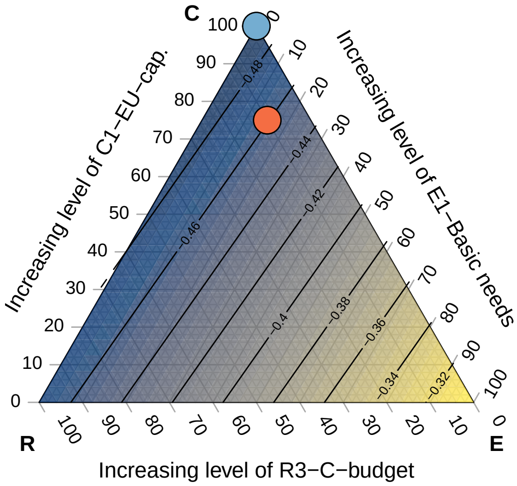

(b) R3-C-budget

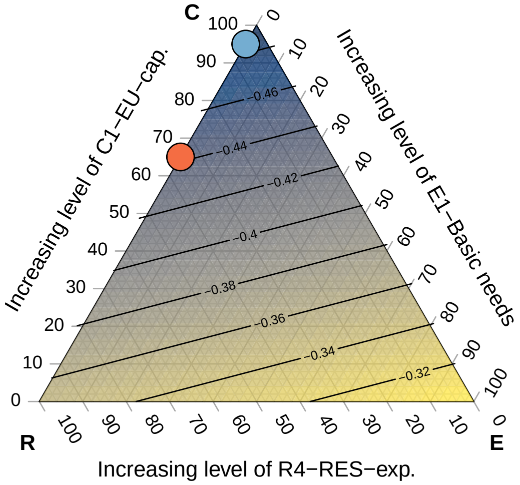

(c) R4-RES-expansion

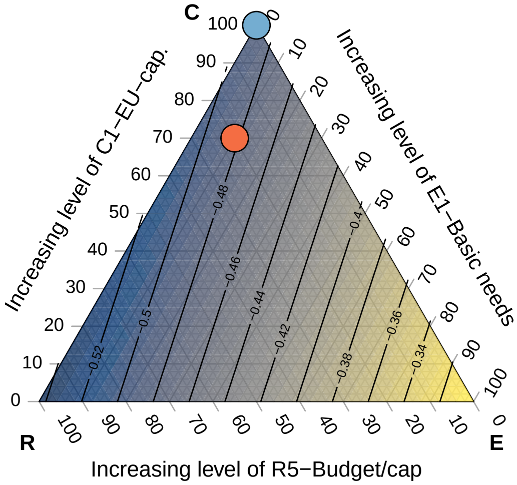

(d) R5-Budget/cap

Supplementary Figure 15. Ternary charts for Germany, cont'd

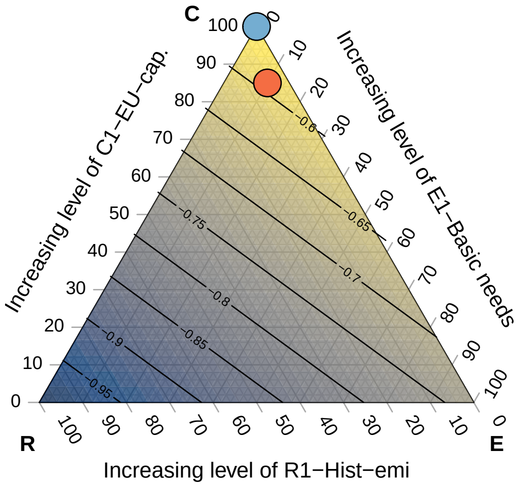

(a) Main

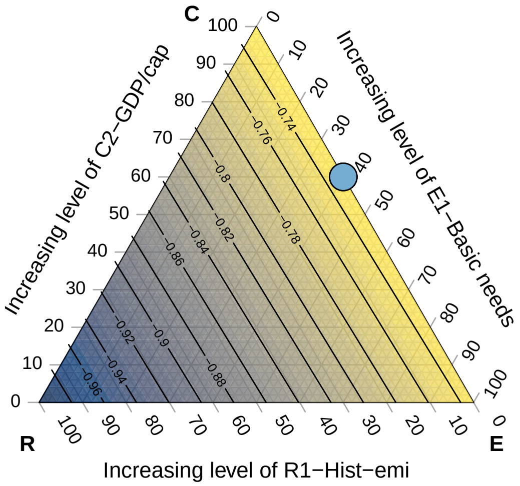

(b) C2-GDP/cap

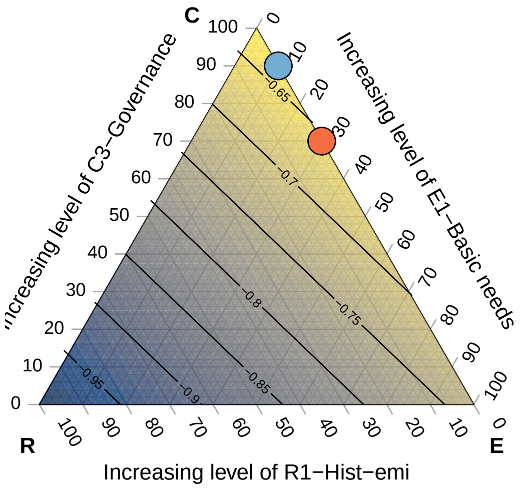

(c) C3-Governance

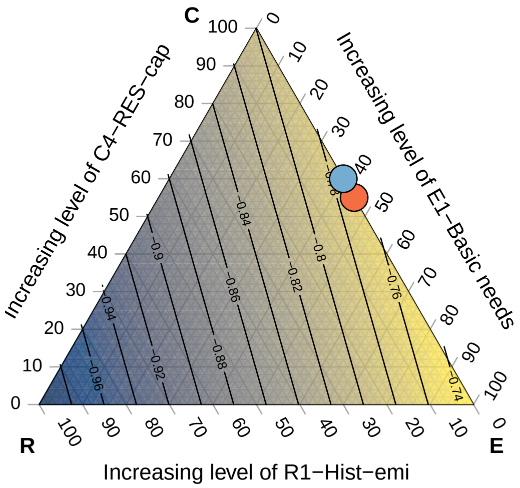

(d) C4-RES-cap.

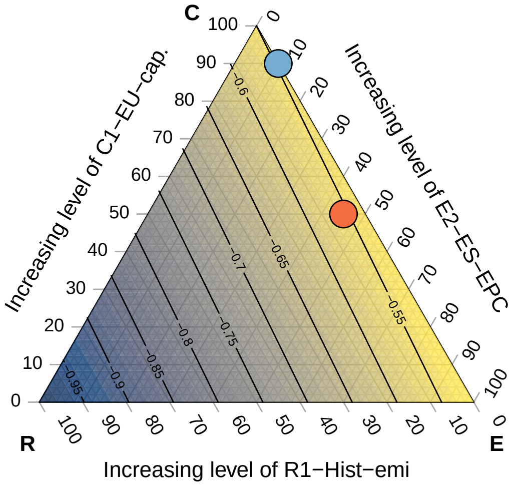

(e) E2-ES-EPC

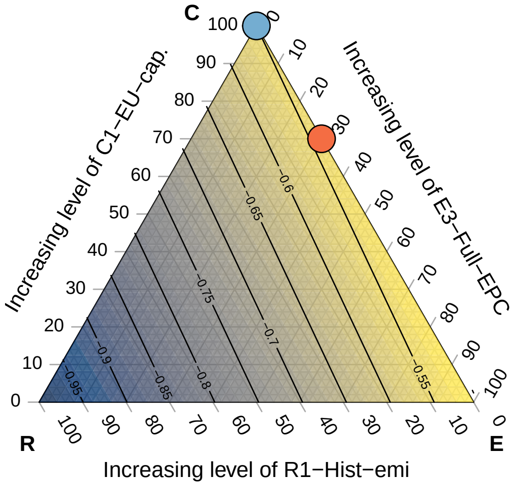

(f) E3-Full-EPC

Supplementary Figure 16. Ternary charts for Denmark: combinations of various equality, responsibility and capability specifications. The current Fit for 55 target for the country is -50%.

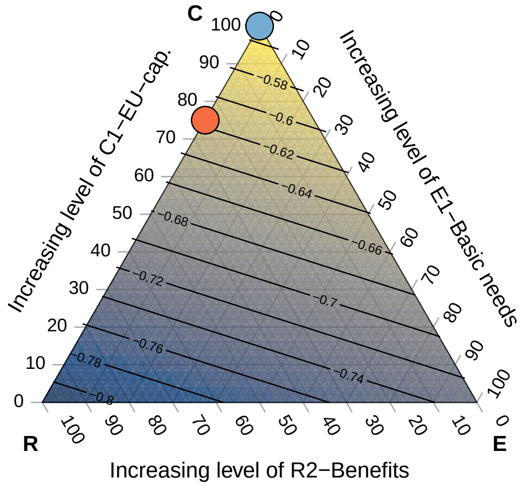

(a) R2-Benefits

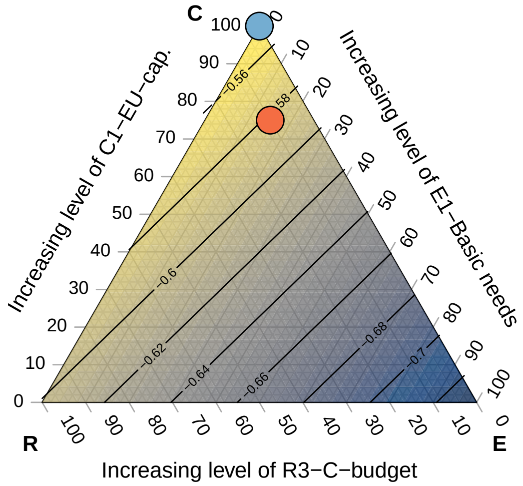

(b) R3-C-budget

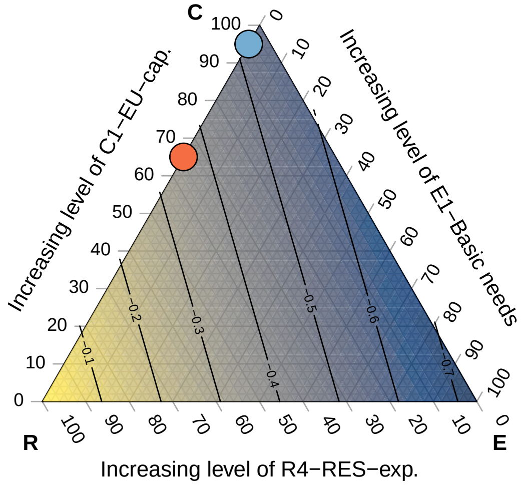

(c) R4-RES-expansion

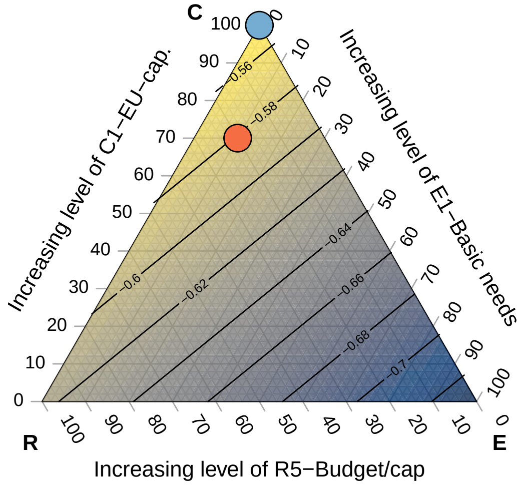

(d) R5-Budget/cap

Supplementary Figure 17. Ternary charts for Denmark, cont'd

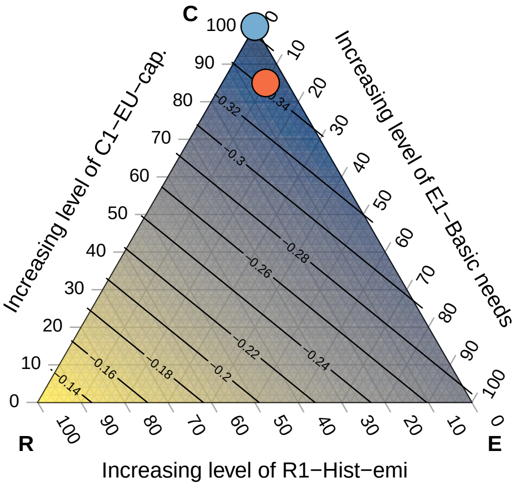

(a) Main

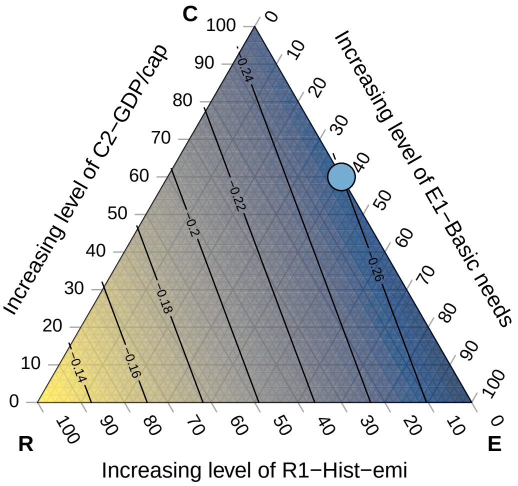

(b) C2-GDP/cap

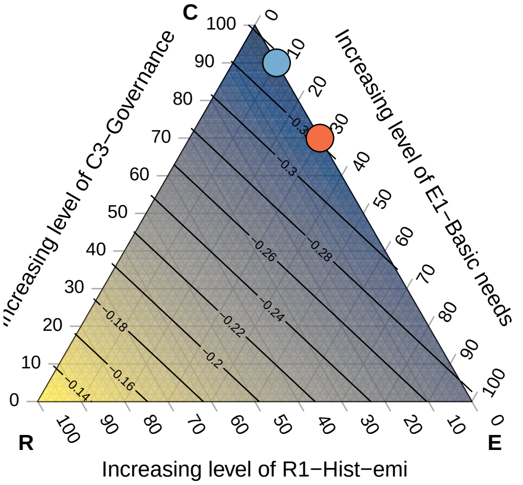

(c) C3-Governance

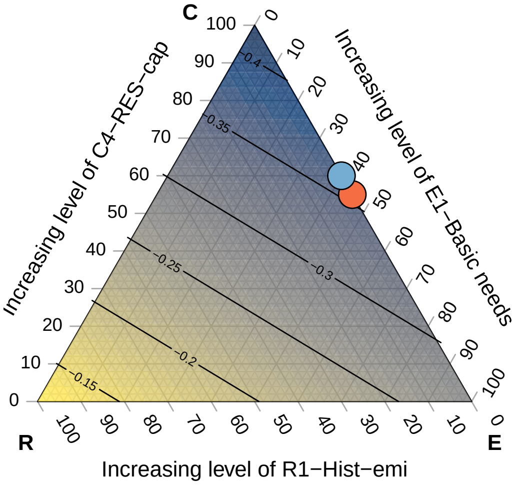

(d) C4-RES-cap.

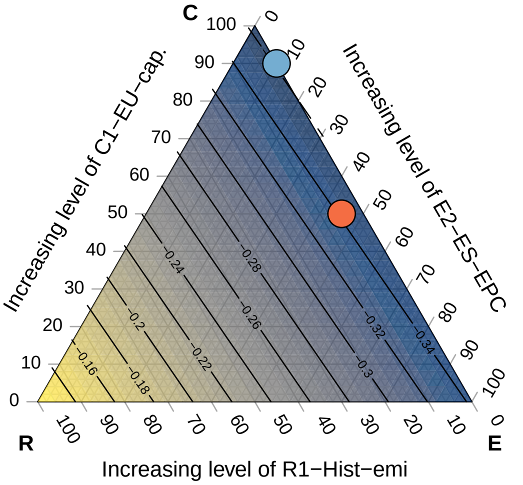

(e) E2-ES-EPC

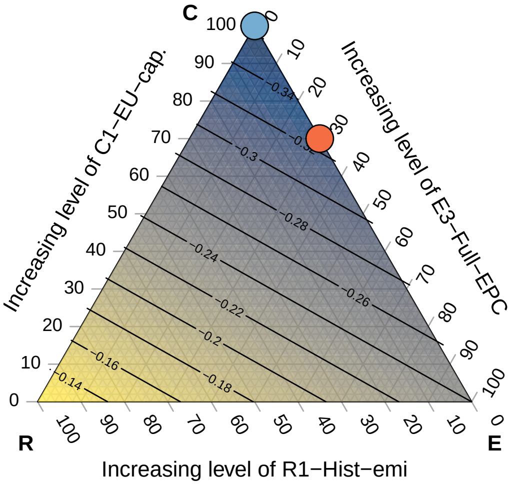

(f) E3-Full-EPC

Supplementary Figure 18. Ternary charts for Spain: combinations of various equality, responsibility and capability specifications. The current Fit for 55 target for the country is -33.7%.

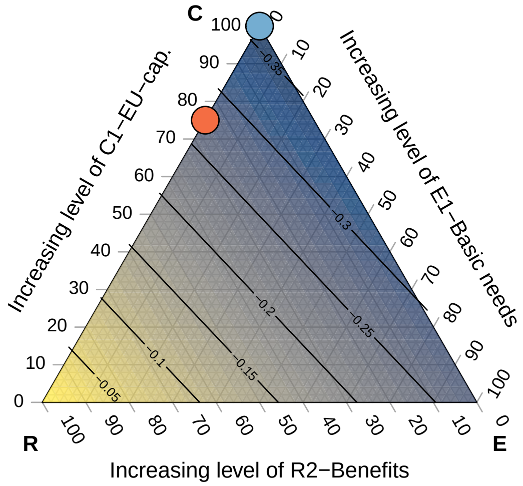

(a) R2-Benefits

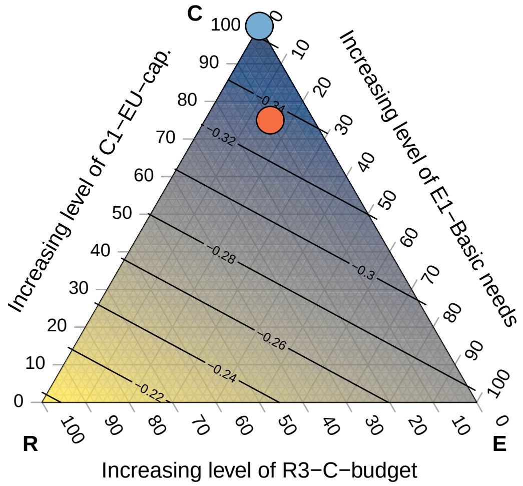

(b) R3-C-budget

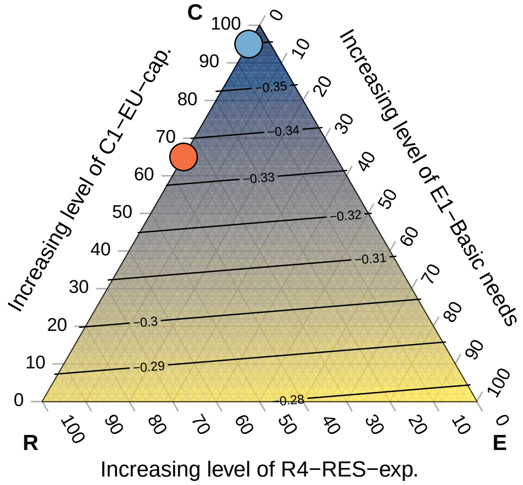

(c) R4-RES-expansion

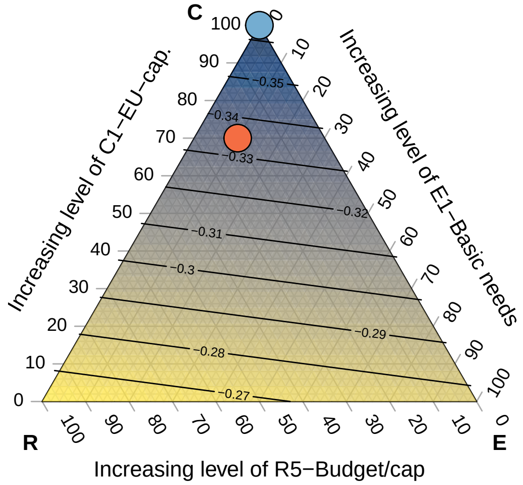

(d) R5-Budget/cap

Supplementary Figure 19. Ternary charts for Spain, cont'd

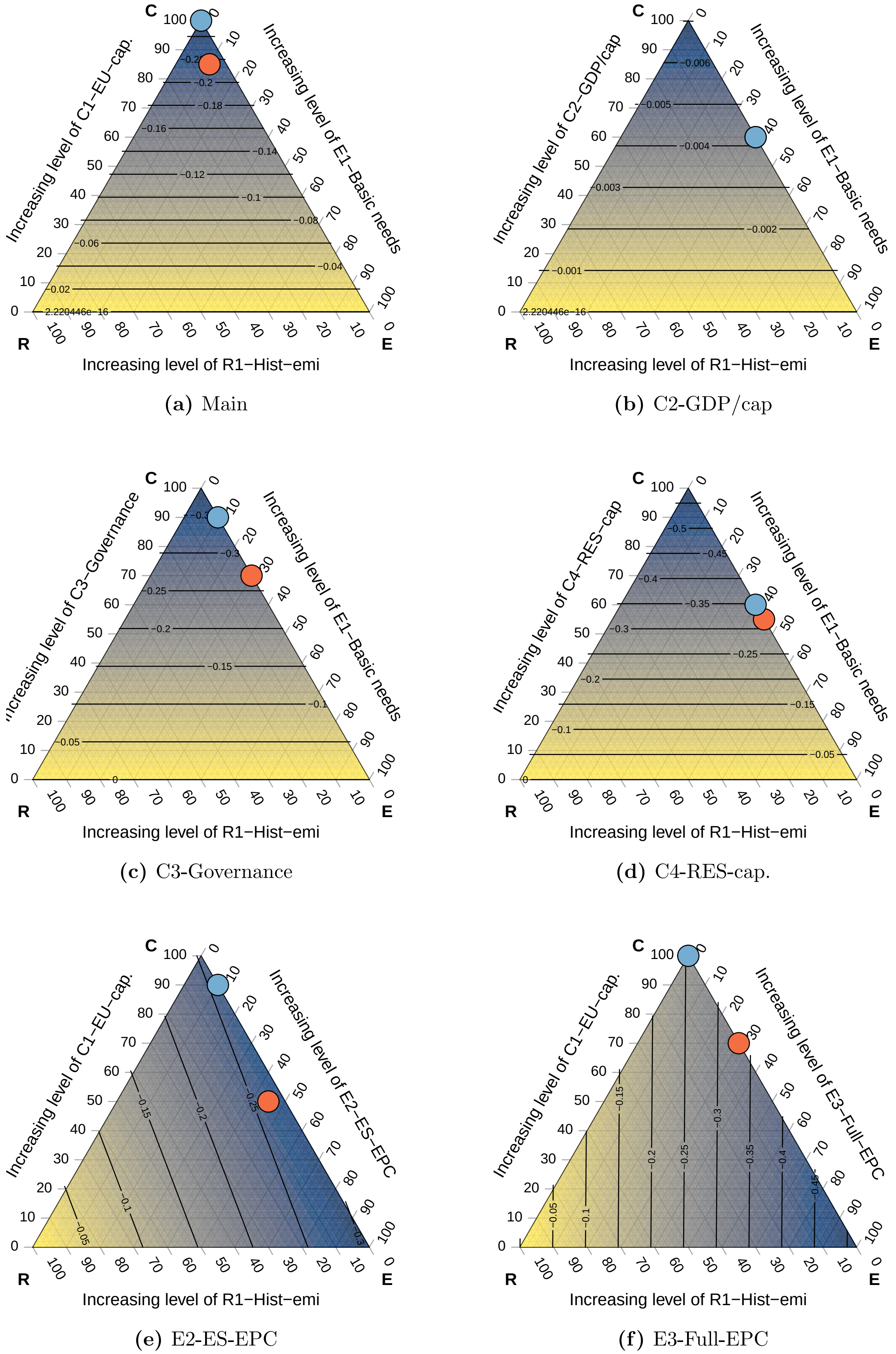

(a) Main

(b) C2-GDP/cap

(c) C3-Governance

(d) C4-RES-cap.

(e) E2-ES-EPC

(f) E3-Full-EPC

Supplementary Figure 20. Ternary charts for Estonia: combinations of various equality, responsibility and capability specifications. The current Fit for 55 target for the country is -24%.

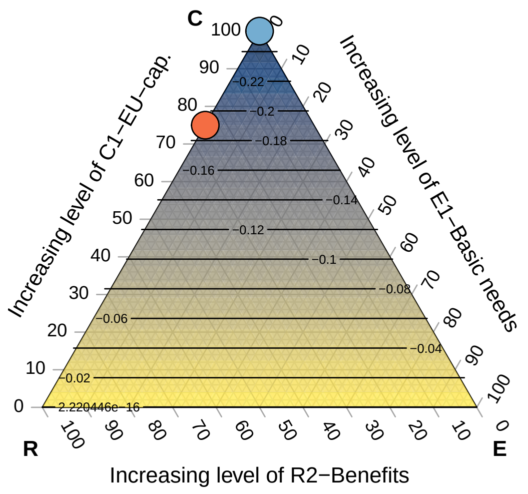

(a) R2-Benefits

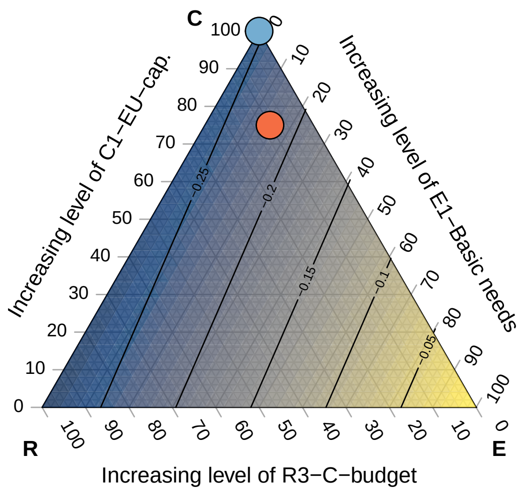

(b) R3-C-budget

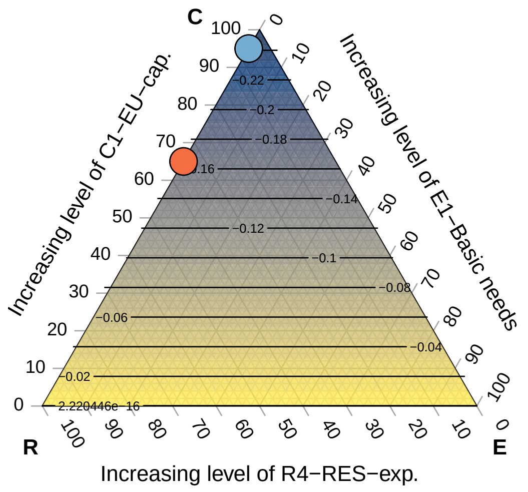

(c) R4-RES-expansion

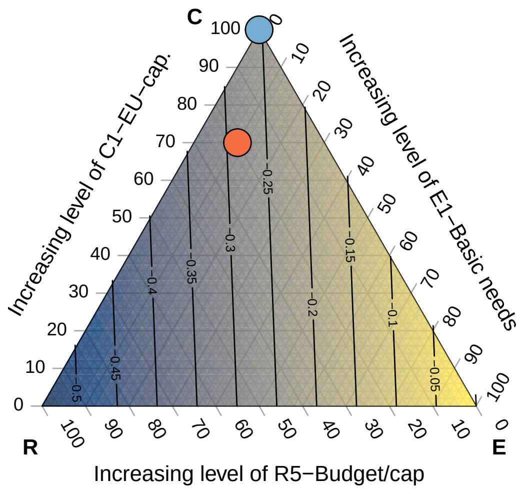

(d) R5-Budget/cap

Supplementary Figure 21. Ternary charts for Estonia, cont'd

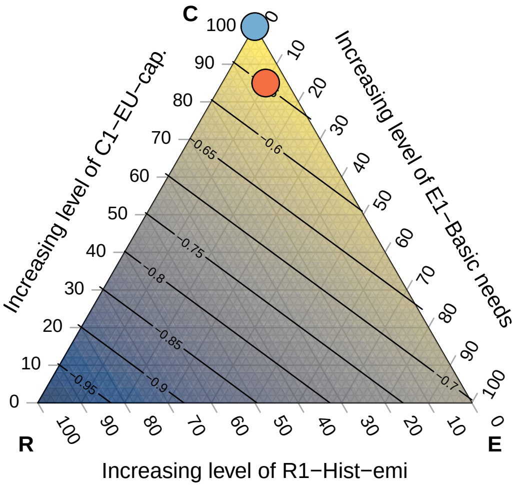

(a) Main

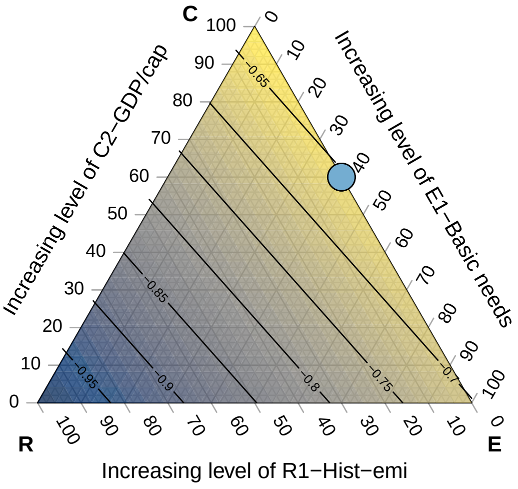

(b) C2-GDP/cap

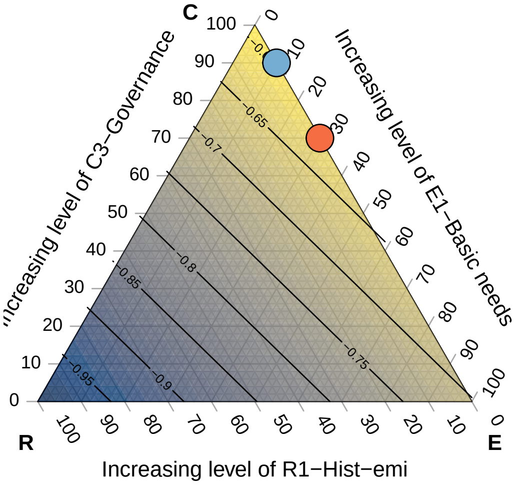

(c) C3-Governance

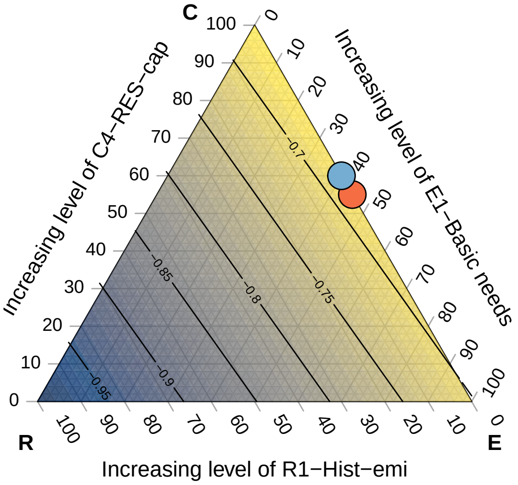

(d) C4-RES-cap.

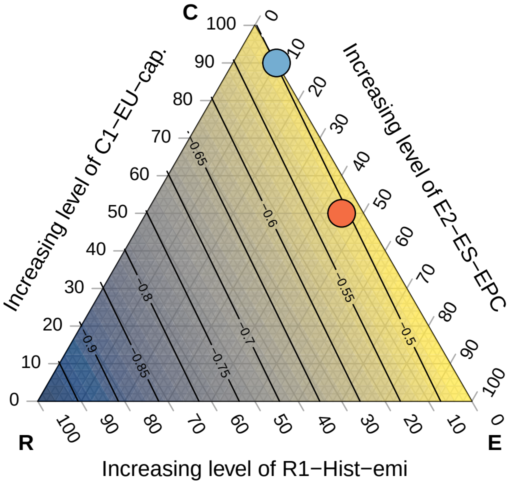

(e) E2-ES-EPC

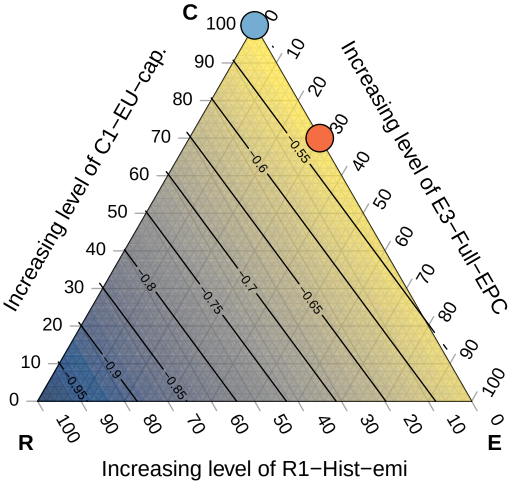

(f) E3-Full-EPC

Supplementary Figure 22. Ternary charts for Finland: combinations of various equality, responsibility and capability specifications. The current Fit for 55 target for the country is -50%.

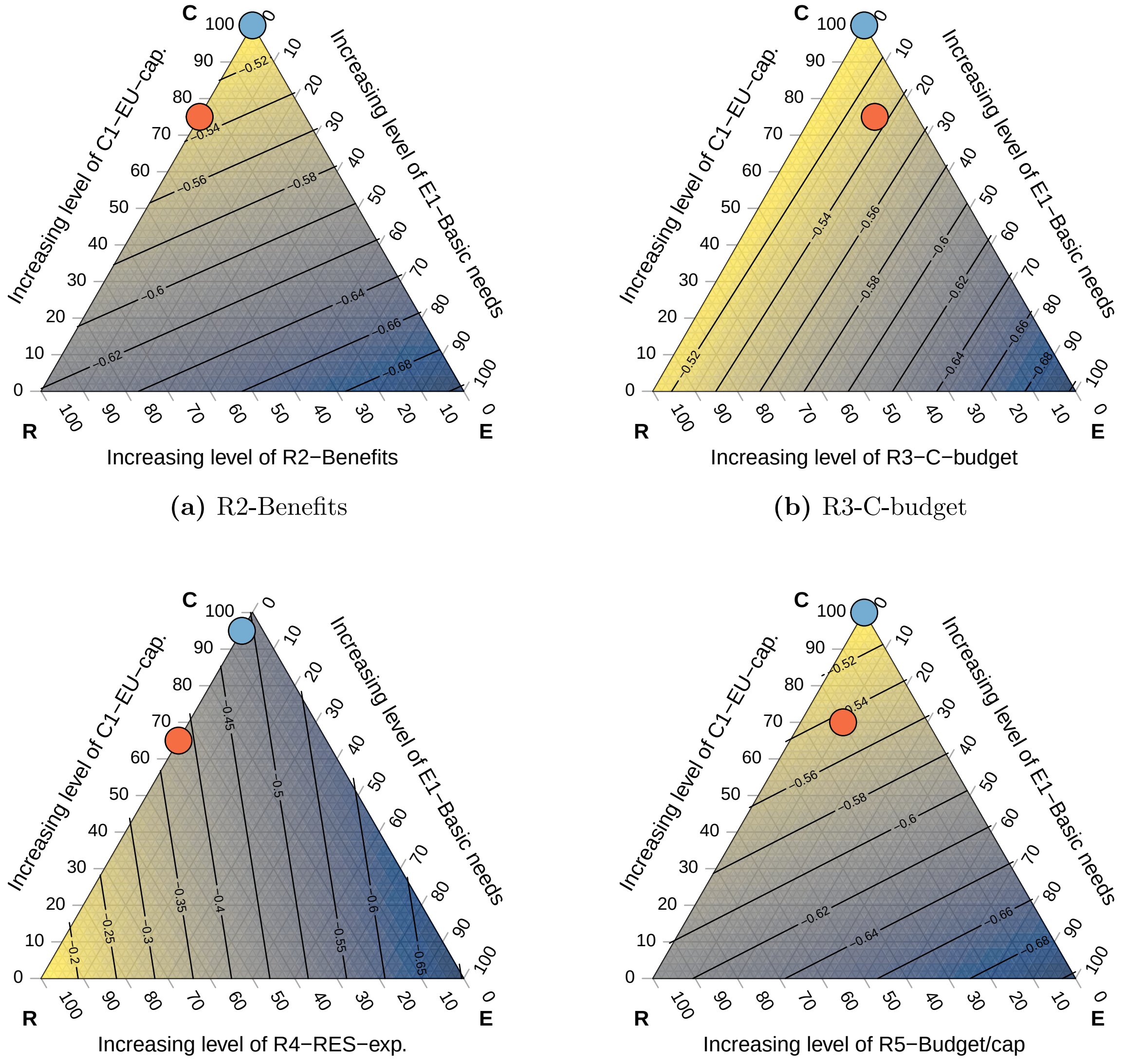

(a) R2-Benefits

(b) R3-C-budget

(c) R4-RES-expansion

(d) R5-Budget/cap

Supplementary Figure 23. Ternary charts for Finland, cont’d

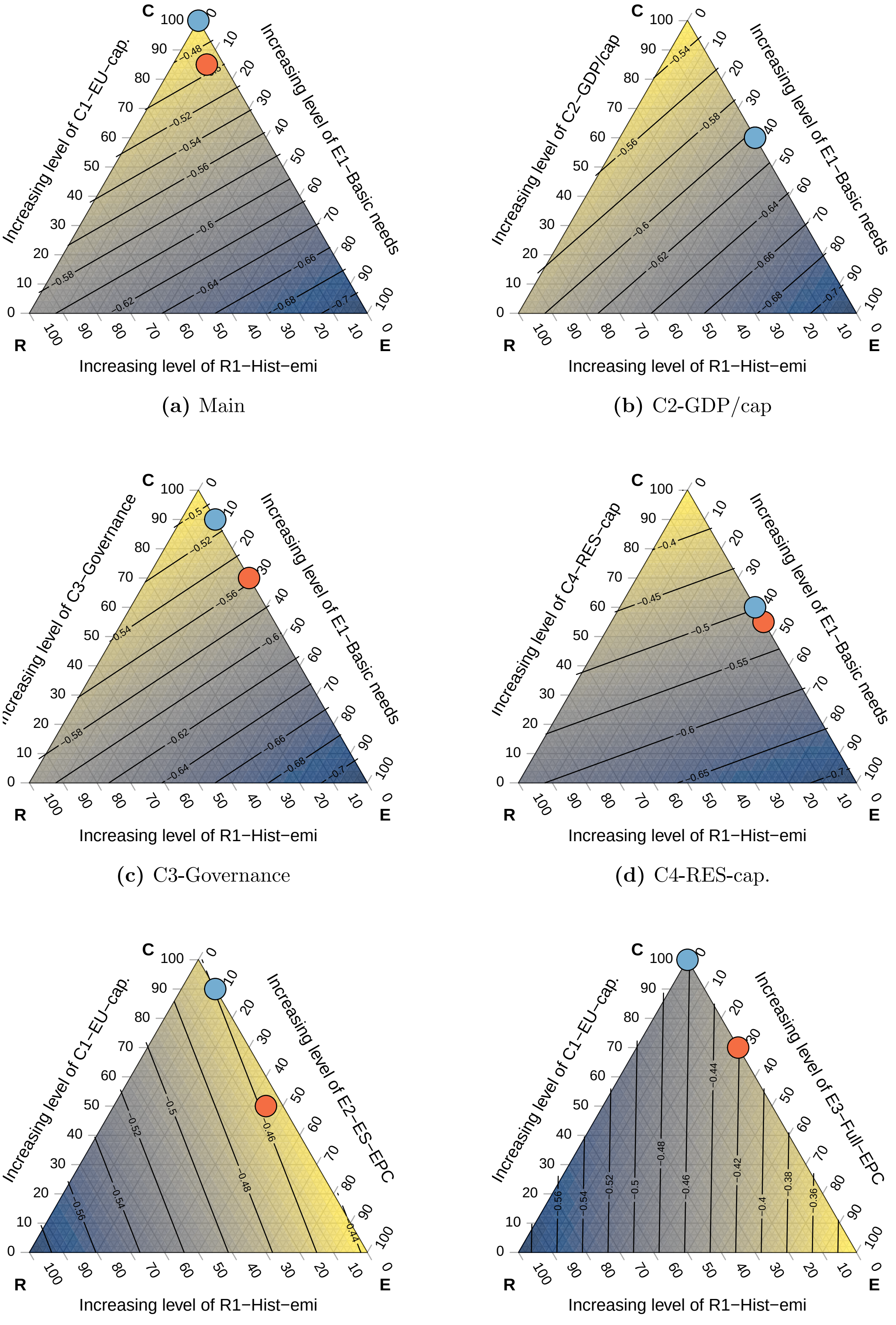

(a) Main

(b) C2-GDP/cap

(c) C3-Governance

(d) C4-RES-cap.

(e) E2-ES-EPC

(f) E3-Full-EPC

Supplementary Figure 24. Ternary charts for France: combinations of various equality, responsibility and capability specifications. The current Fit for 55 target for the country is -47.5%.

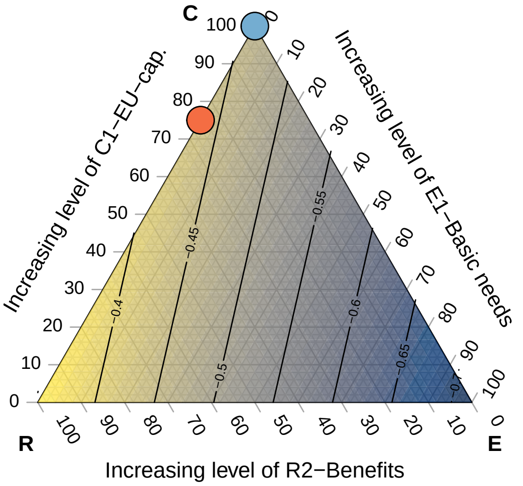

(a) R2-Benefits

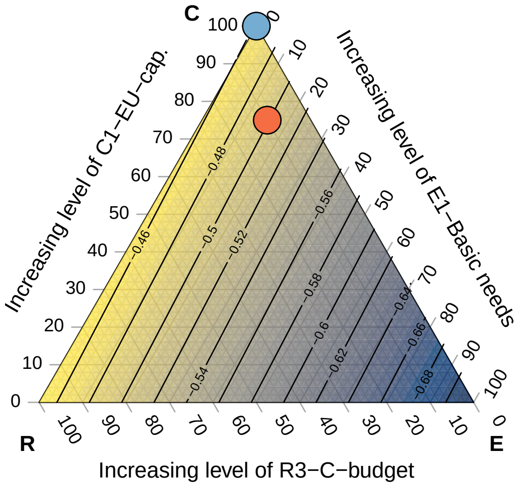

(b) R3-C-budget

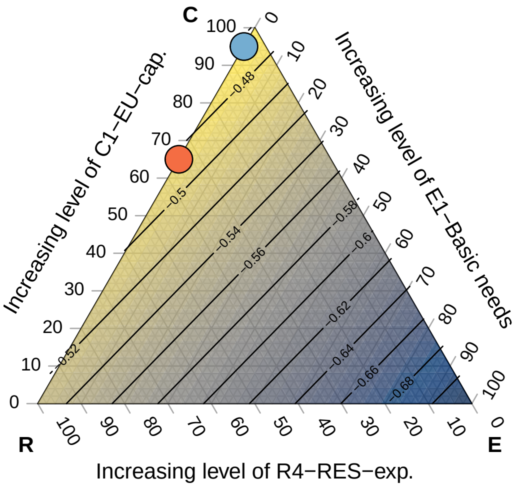

(c) R4-RES-expansion

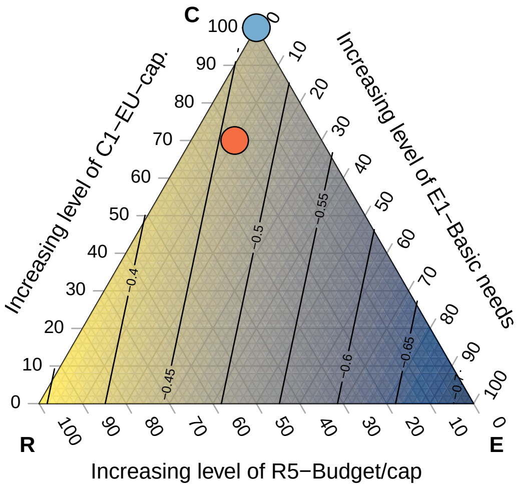

(d) R5-Budget/cap

Supplementary Figure 25. Ternary charts for France, cont'd

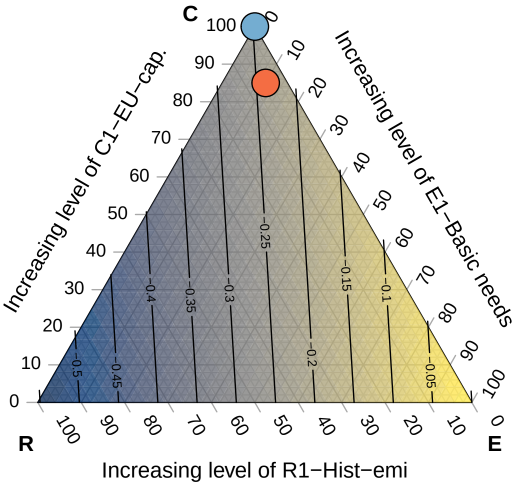

(a) Main

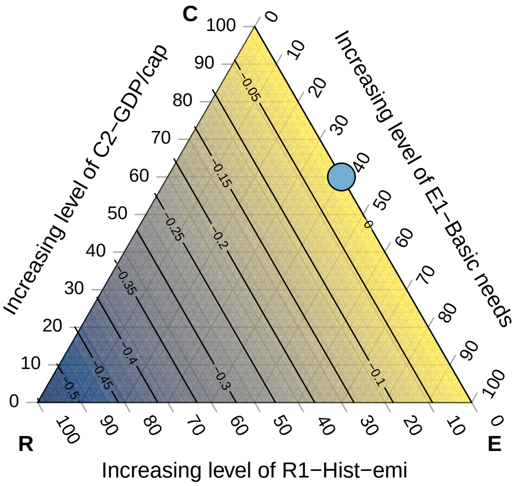

(b) C2-GDP/cap

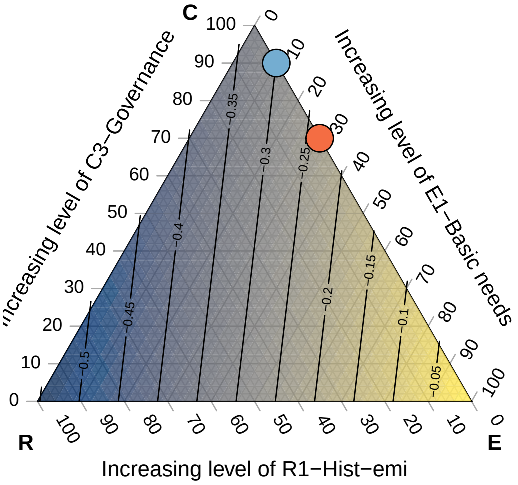

(c) C3-Governance

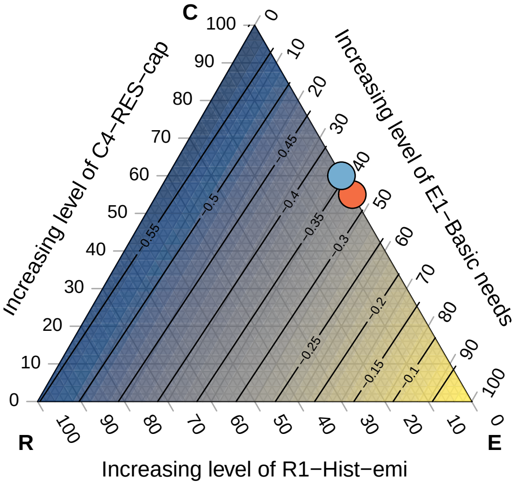

(d) C4-RES-cap.

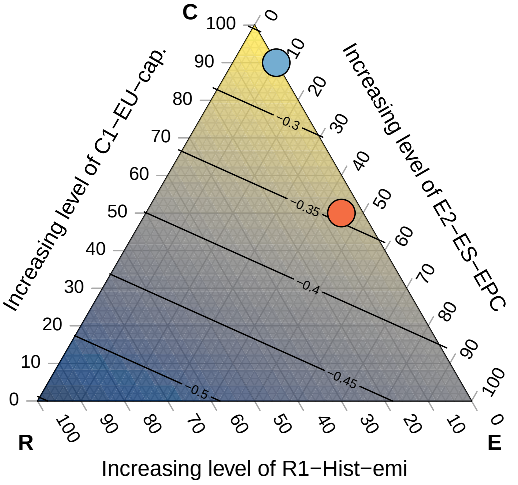

(e) E2-ES-EPC

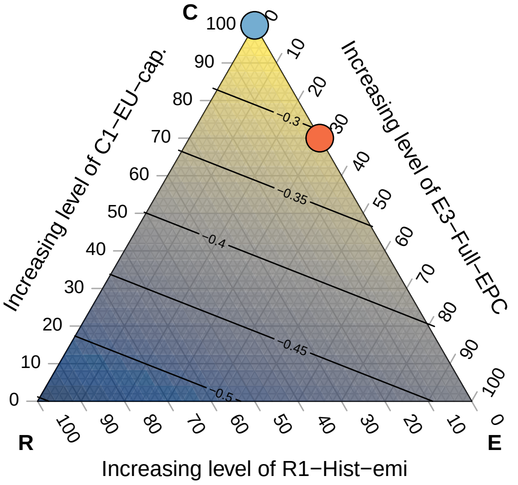

(f) E3-Full-EPC

Supplementary Figure 26. Ternary charts for Greece: combinations of various equality, responsibility and capability specifications. The current Fit for 55 target for the country is -22.7%.

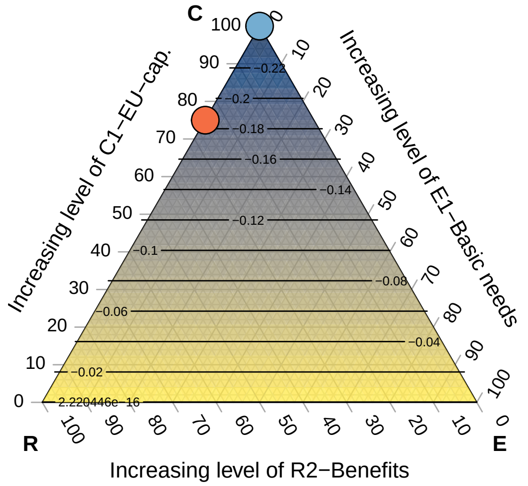

(a) R2-Benefits

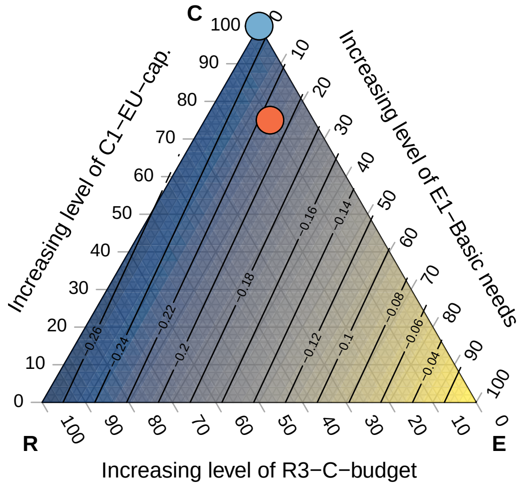

(b) R3-C-budget

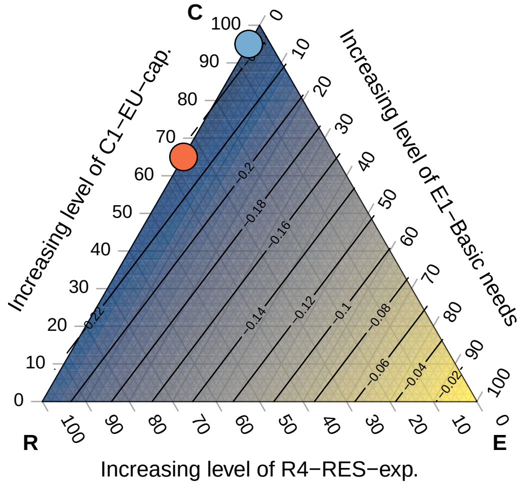

(c) R4-RES-expansion

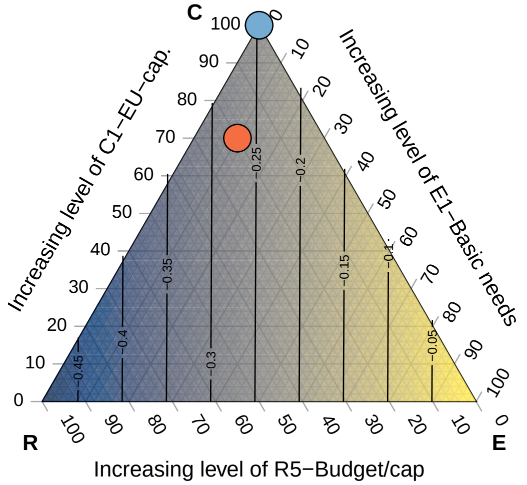

(d) R5-Budget/cap

Supplementary Figure 27. Ternary charts for Greece, cont'd

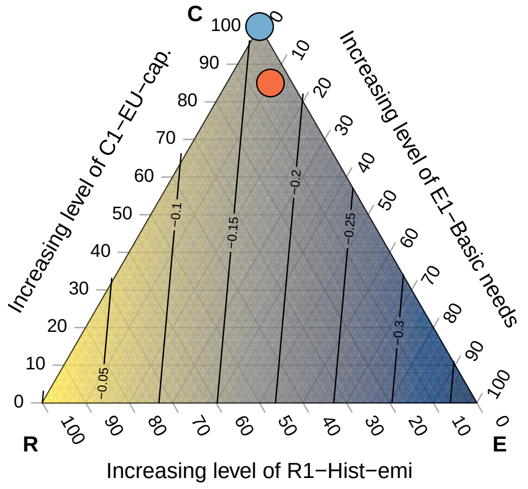

(a) Main

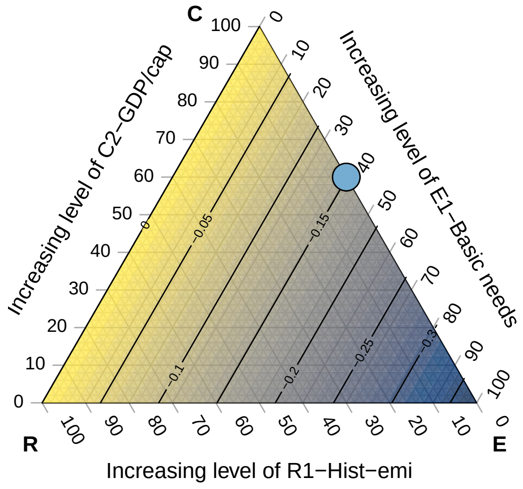

(b) C2-GDP/cap

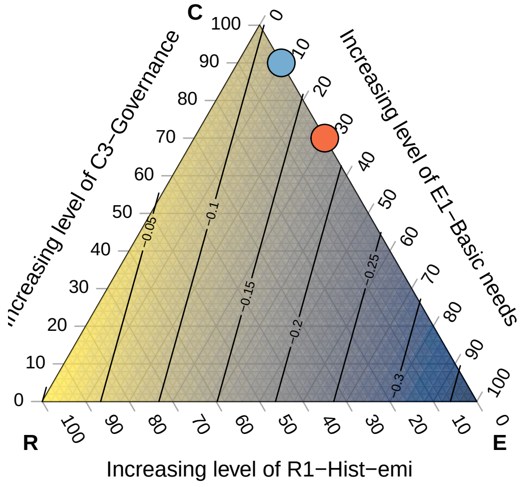

(c) C3-Governance

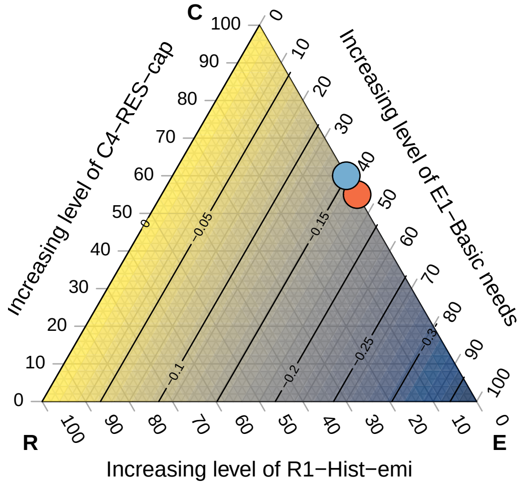

(d) C4-RES-cap.

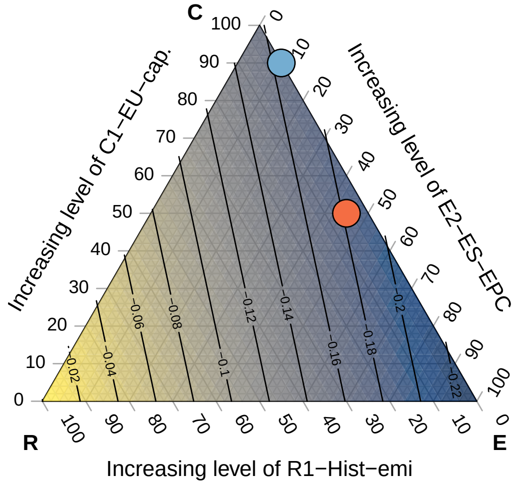

(e) E2-ES-EPC

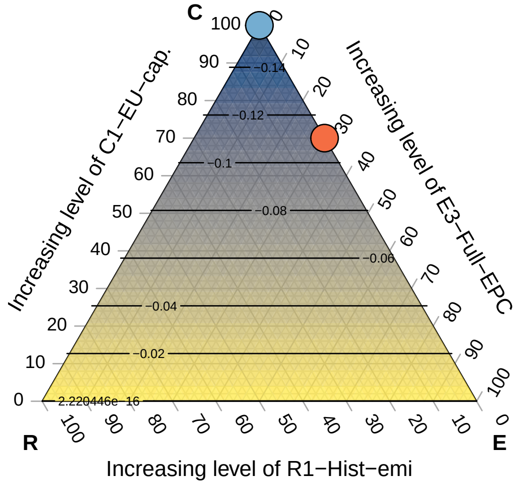

(f) E3-Full-EPC

Supplementary Figure 28. Ternary charts for Croatia: combinations of various equality, responsibility and capability specifications. The current Fit for 55 target for the country is -16.5%.

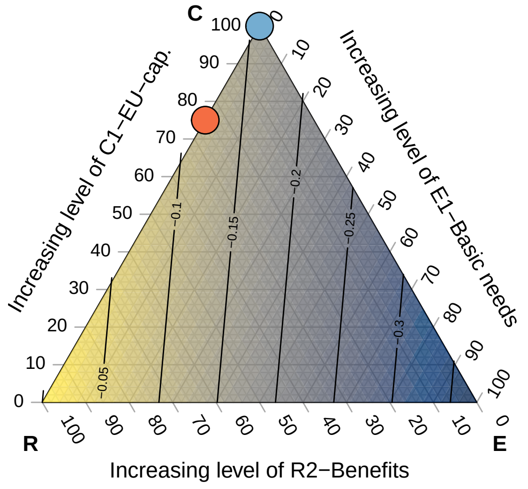

(a) R2-Benefits

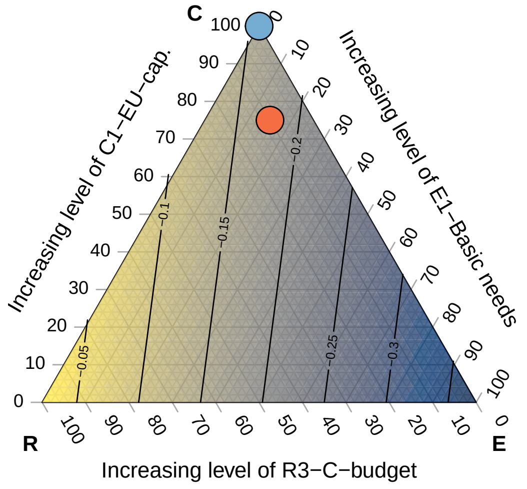

(b) R3-C-budget

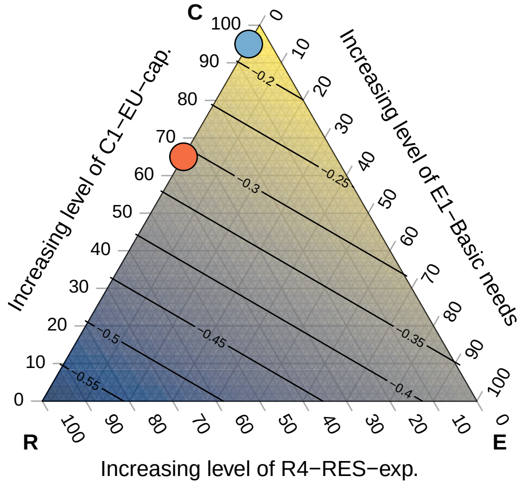

(c) R4-RES-expansion

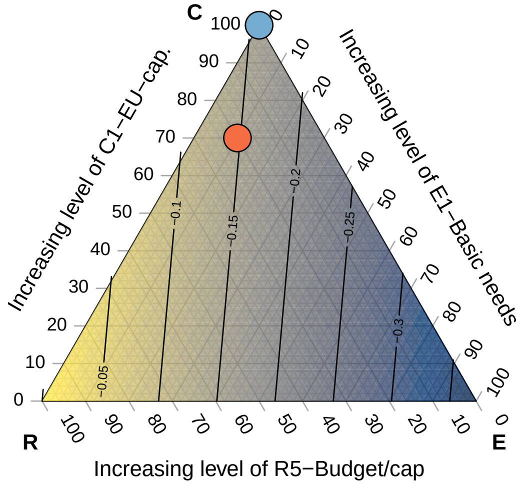

(d) R5-Budget/cap

Supplementary Figure 29. Ternary charts for Croatia, cont'd

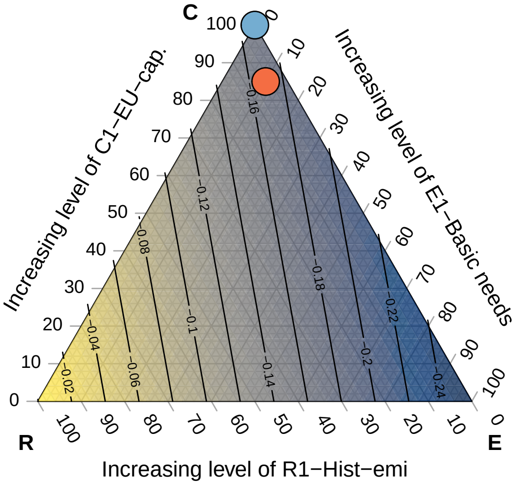

(a) Main

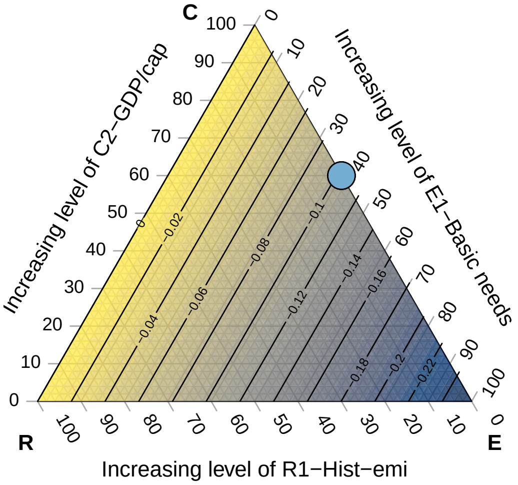

(b) C2-GDP/cap

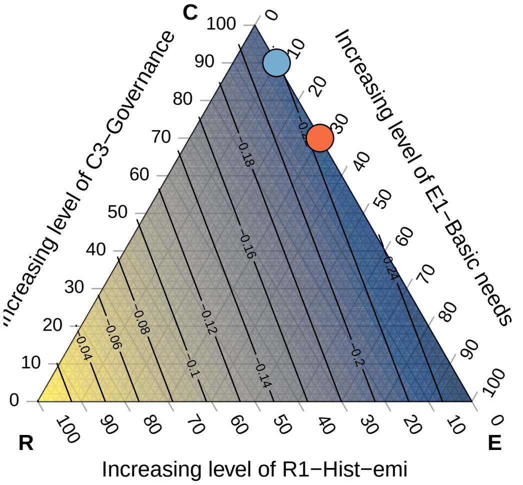

(c) C3-Governance

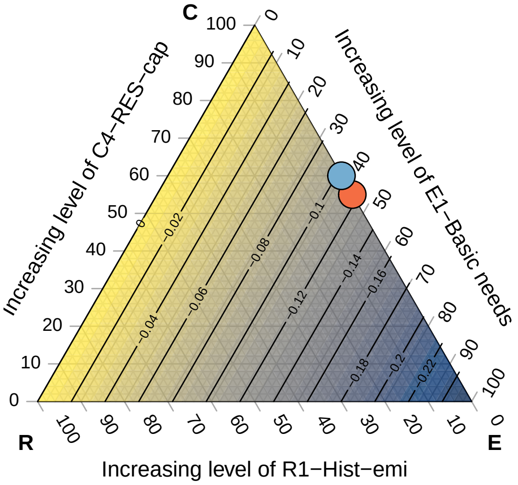

(d) C4-RES-cap.

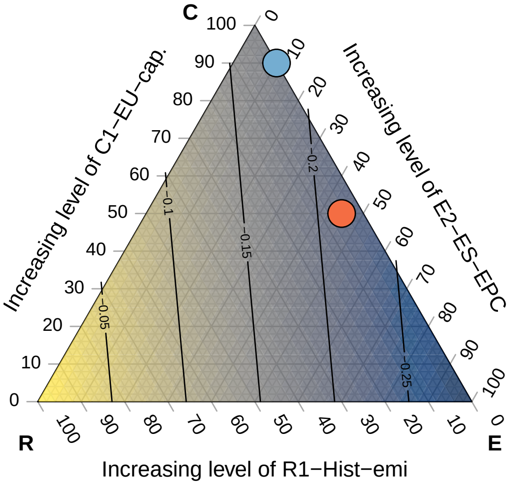

(e) E2-ES-EPC

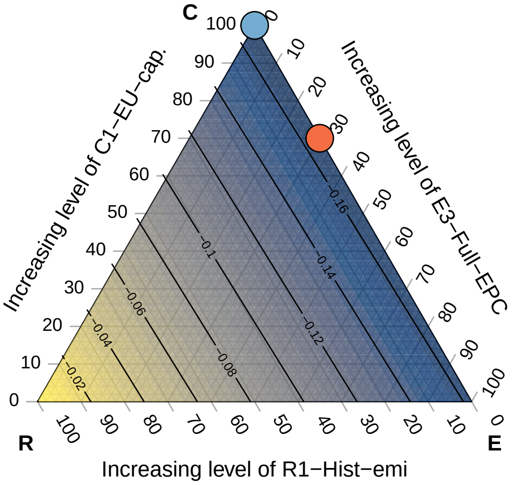

(f) E3-Full-EPC

Supplementary Figure 30. Ternary charts for Hungary: combinations of various equality, responsibility and capability specifications. The current Fit for 55 target for the country is -18.7%.

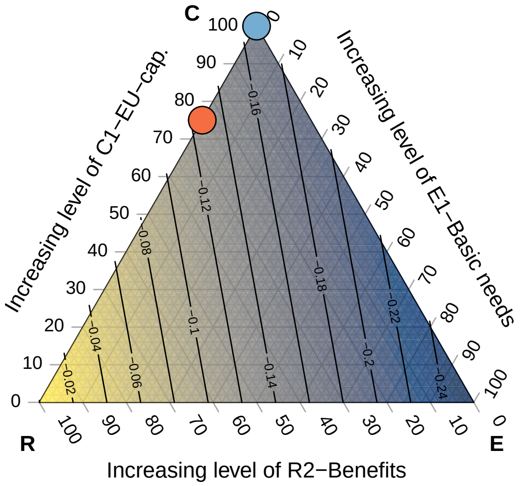

(a) R2-Benefits

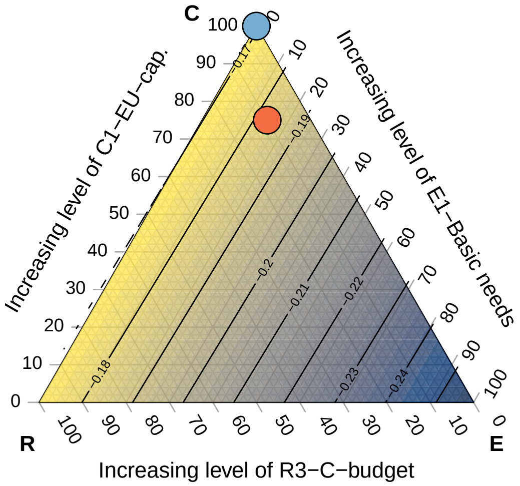

(b) R3-C-budget

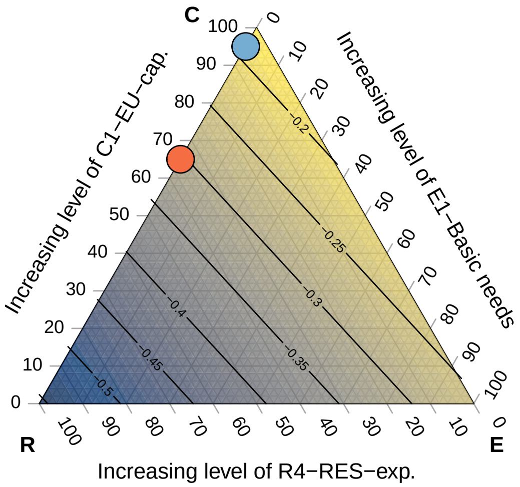

(c) R4-RES-expansion

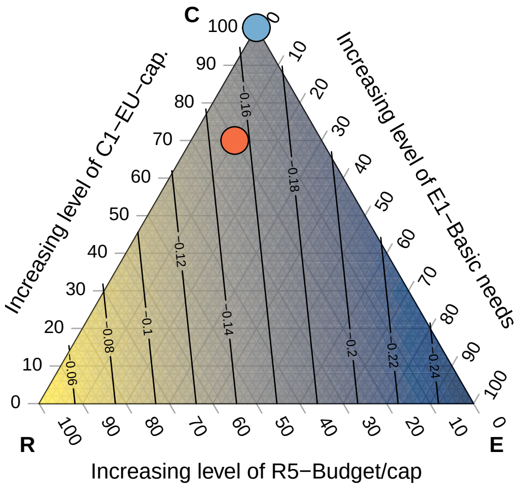

(d) R5-Budget/cap

Supplementary Figure 31. Ternary charts for Hungary, cont'd

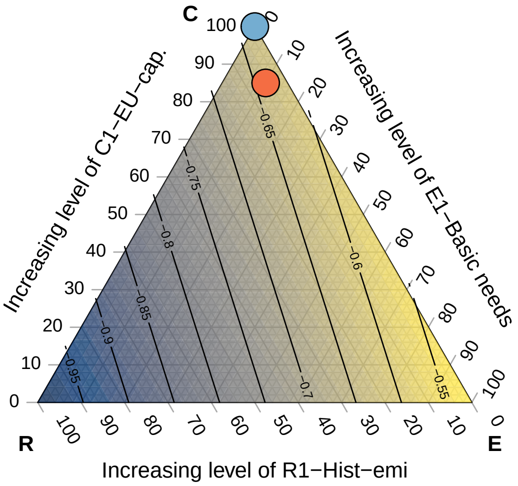

(a) Main

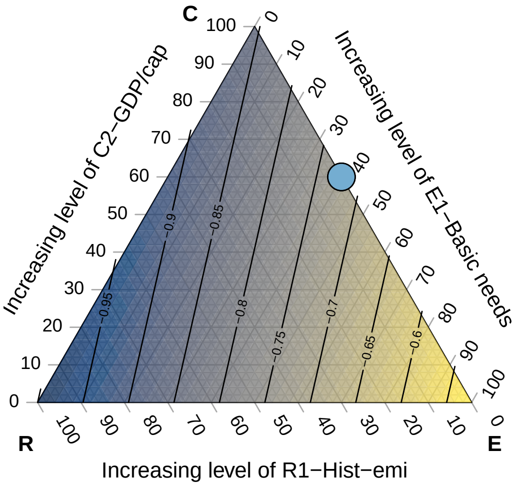

(b) C2-GDP/cap

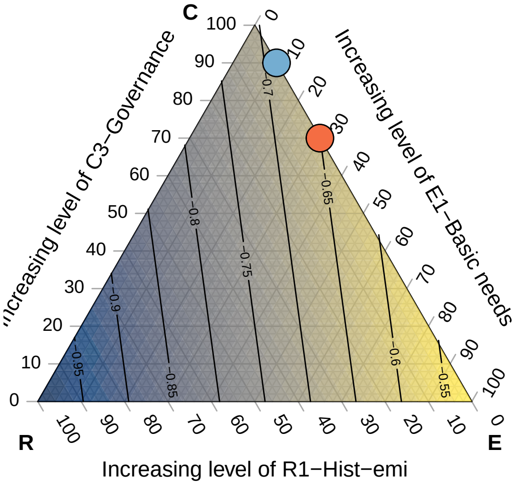

(c) C3-Governance

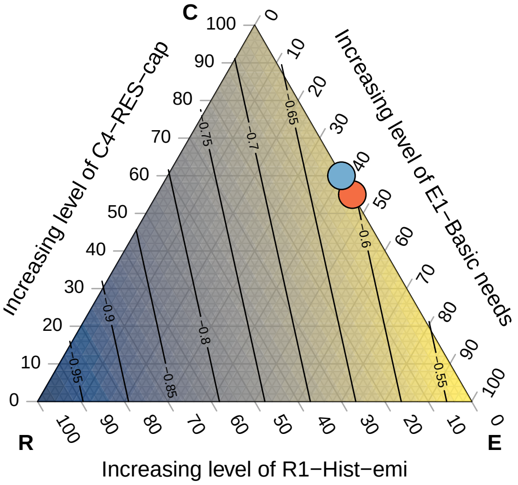

(d) C4-RES-cap.

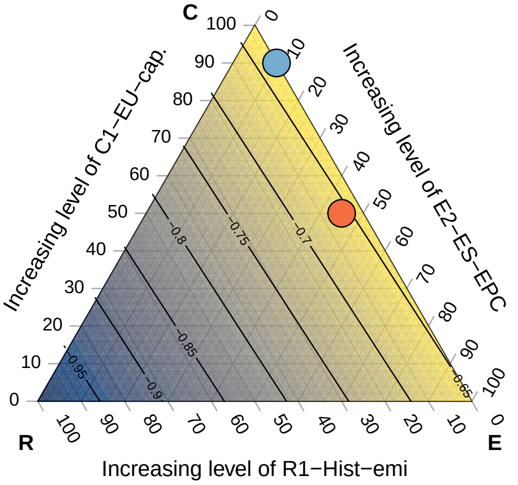

(e) E2-ES-EPC

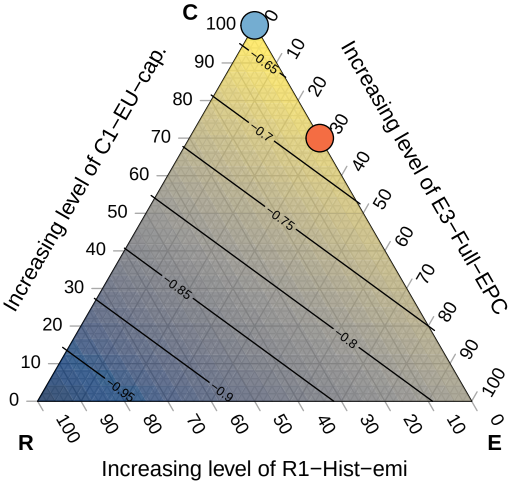

(f) E3-Full-EPC

Supplementary Figure 32. Ternary charts for Ireland: combinations of various equality, responsibility and capability specifications. The current Fit for 55 target for the country is -42%.

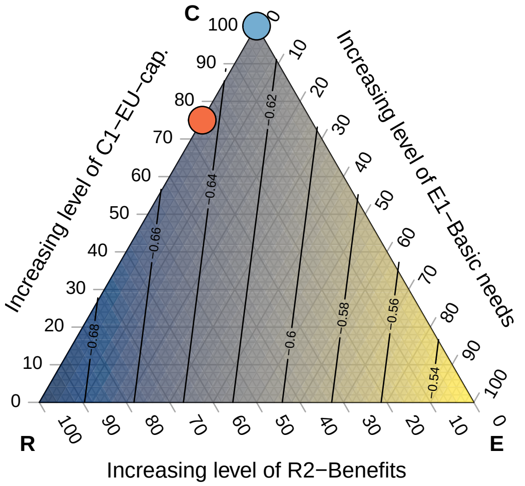

(a) R2-Benefits

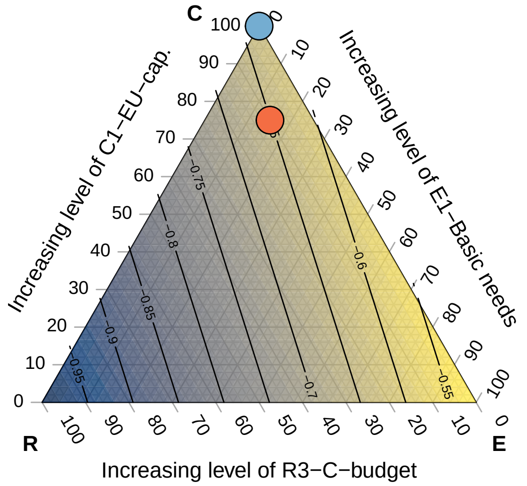

(b) R3-C-budget

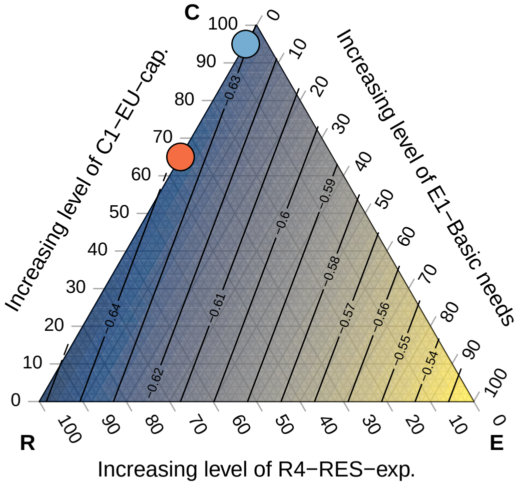

(c) R4-RES-expansion

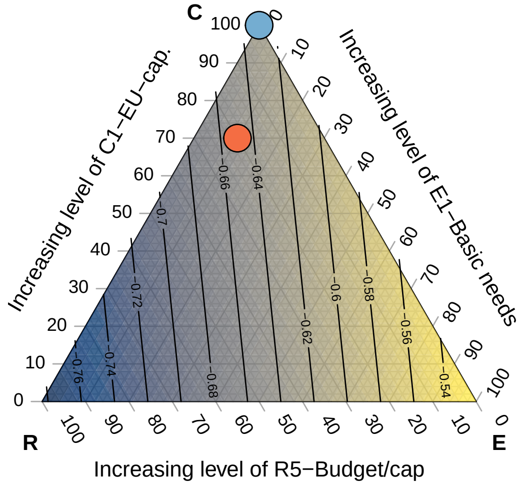

(d) R5-Budget/cap

Supplementary Figure 33. Ternary charts for Ireland, cont'd

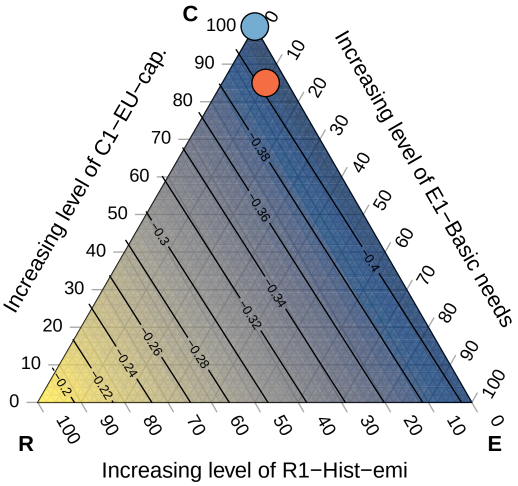

(a) Main

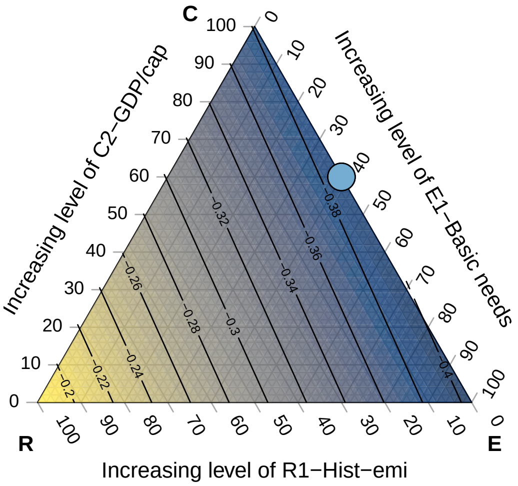

(b) C2-GDP/cap

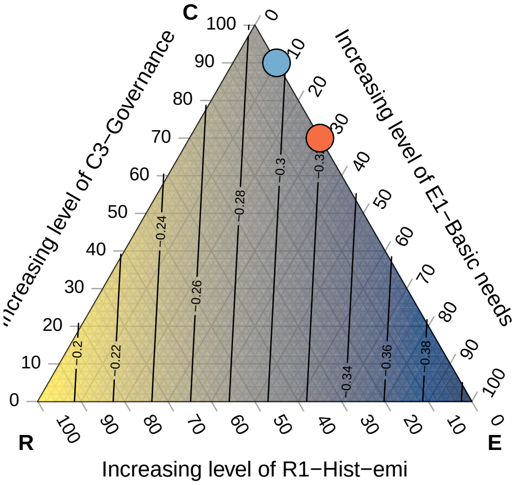

(c) C3-Governance

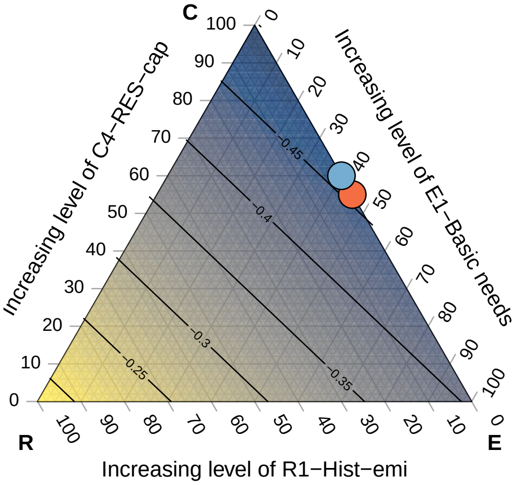

(d) C4-RES-cap.

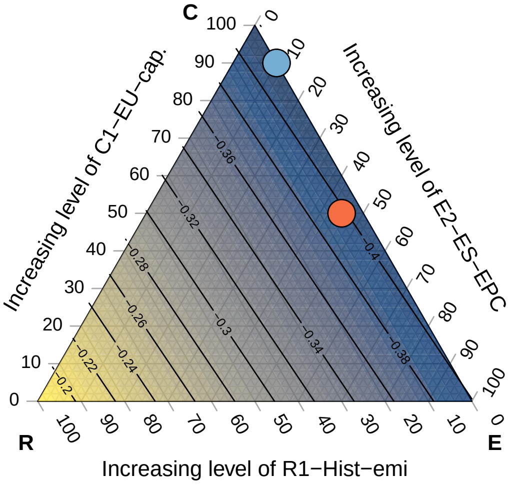

(e) E2-ES-EPC

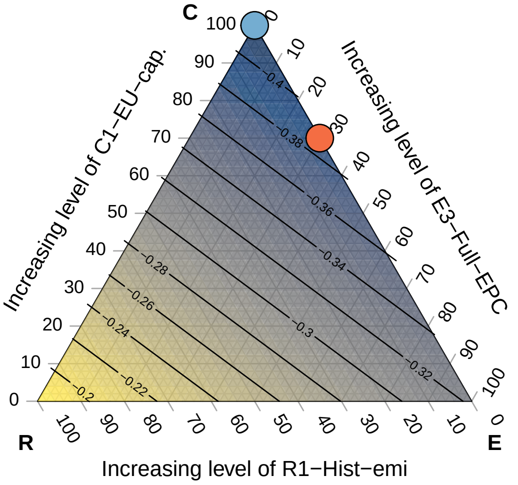

(f) E3-Full-EPC

Supplementary Figure 34. Ternary charts for Italy: combinations of various equality, responsibility and capability specifications. The current Fit for 55 target for the country is -43.7%.

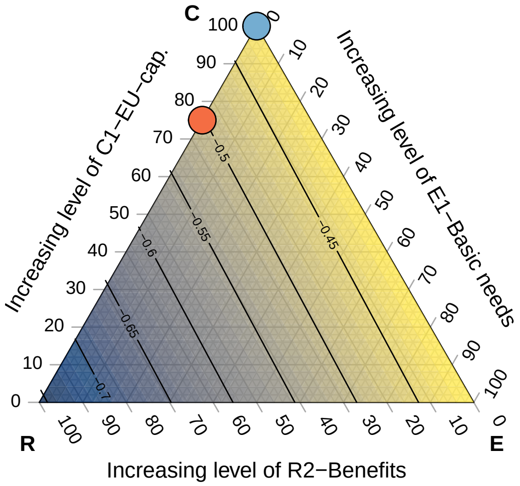

(a) R2-Benefits

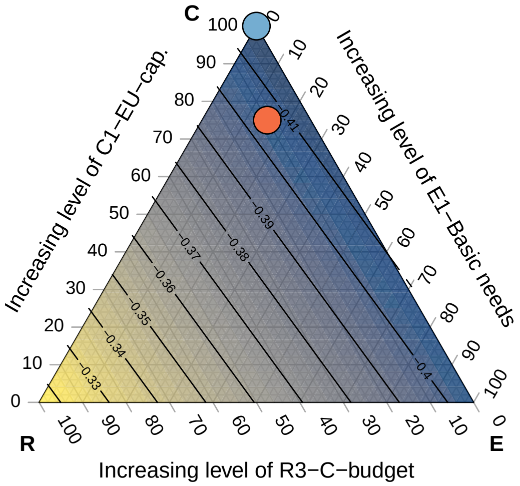

(b) R3-C-budget

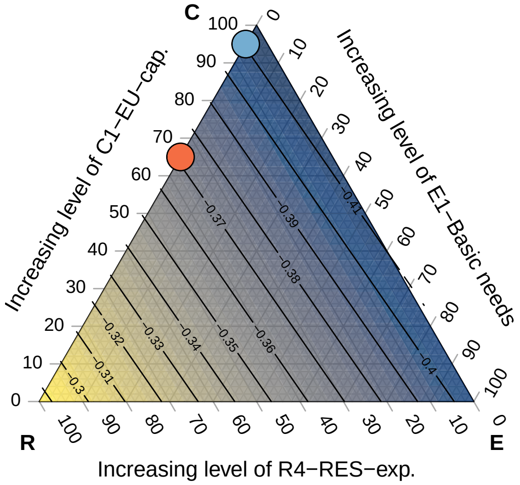

(c) R4-RES-expansion

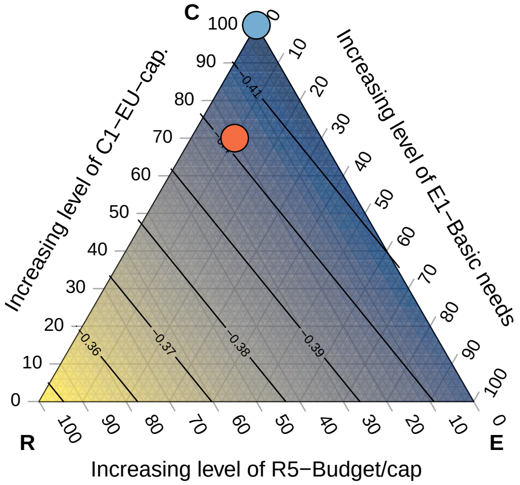

(d) R5-Budget/cap

Supplementary Figure 35. Ternary charts for Italy, cont'd

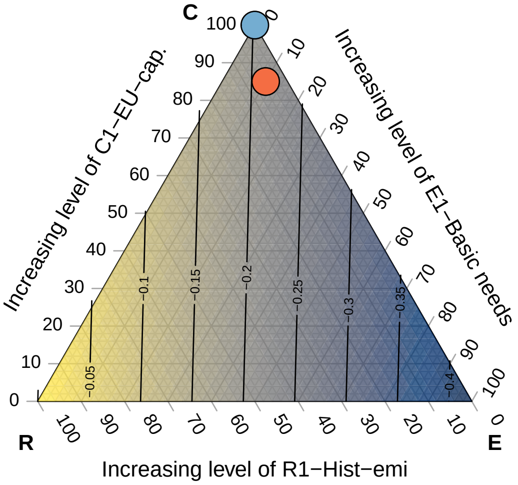

(a) Main

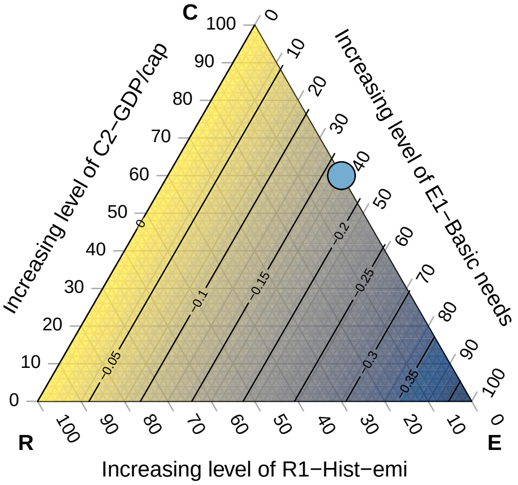

(b) C2-GDP/cap

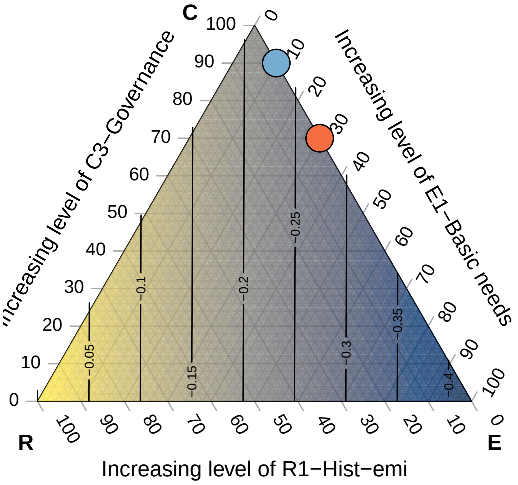

(c) C3-Governance

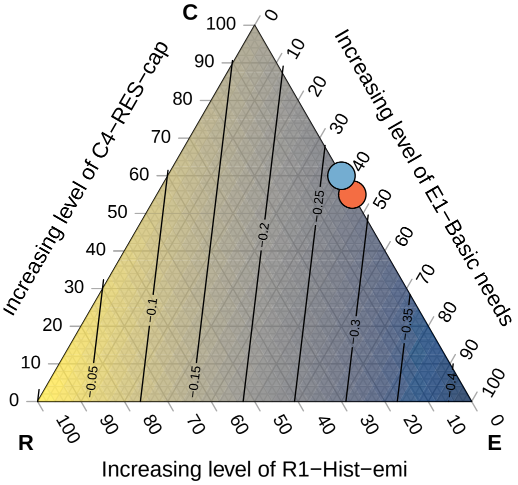

(d) C4-RES-cap.

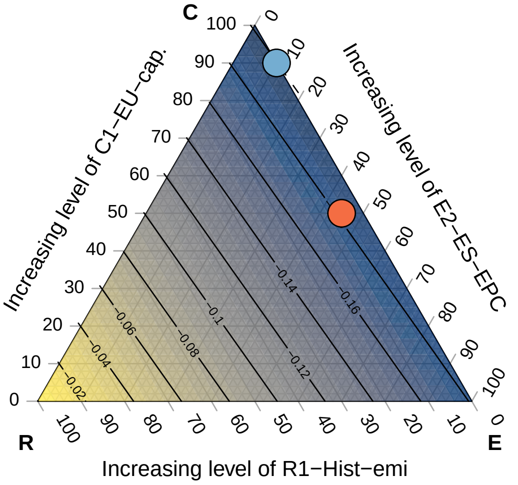

(e) E2-ES-EPC

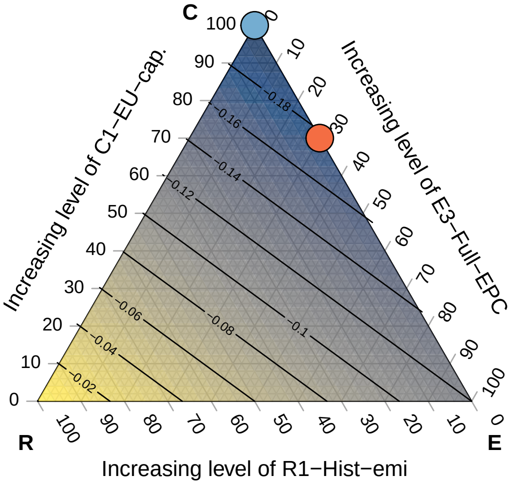

(f) E3-Full-EPC

Supplementary Figure 36. Ternary charts for Lithuania: combinations of various equality, responsibility and capability specifications. The current Fit for 55 target for the country is -21%.

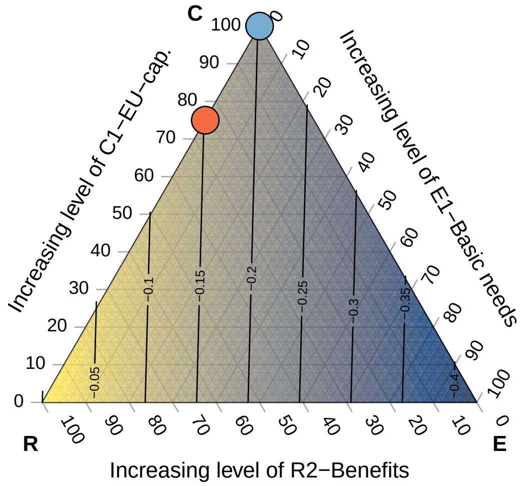

(a) R2-Benefits

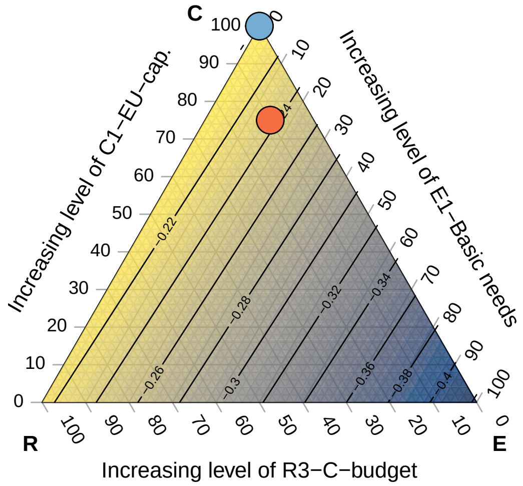

(b) R3-C-budget

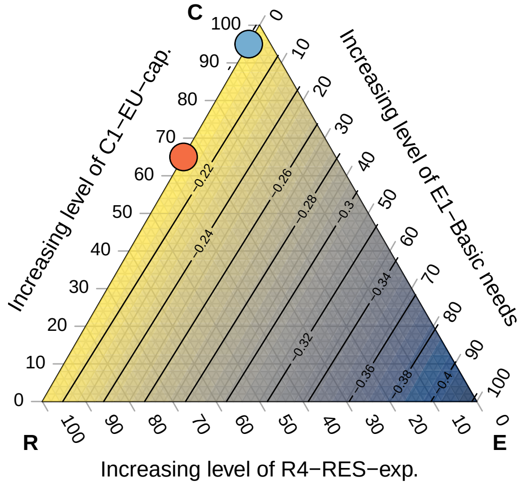

(c) R4-RES-expansion

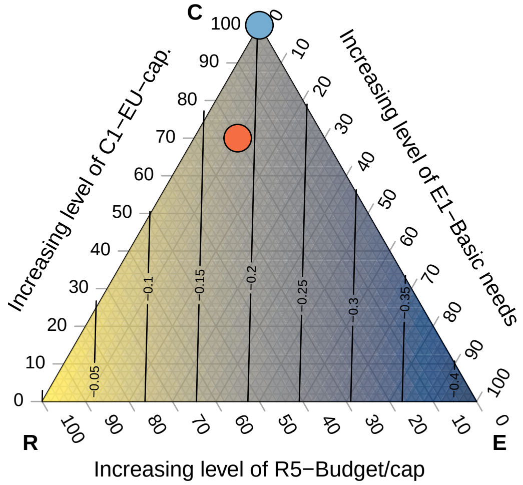

(d) R5-Budget/cap

Supplementary Figure 37. Ternary charts for Lithuania, cont'd

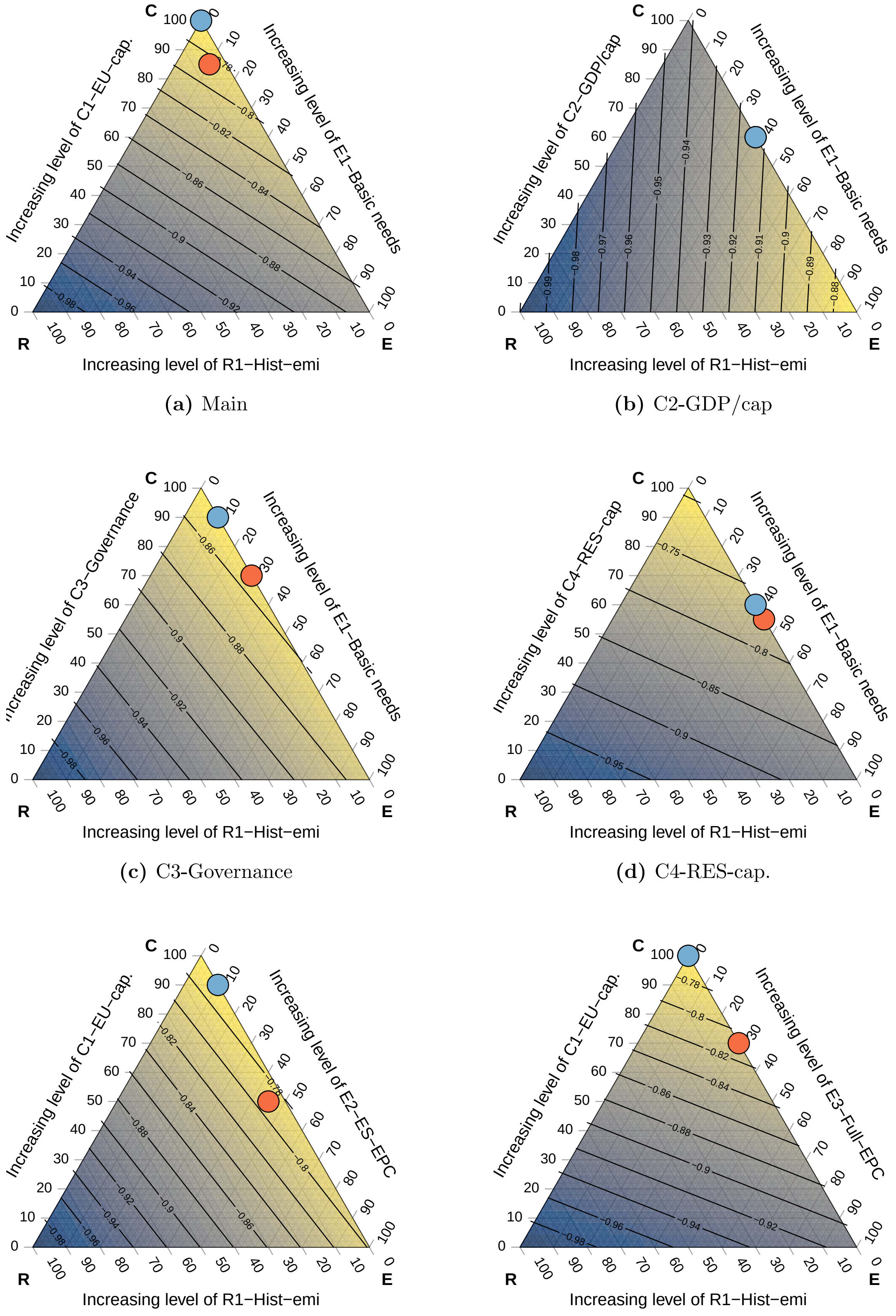

(a) Main

(b) C2-GDP/cap

(c) C3-Governance

(d) C4-RES-cap.

(e) E2-ES-EPC

(f) E3-Full-EPC

Supplementary Figure 38. Ternary charts for Luxembourg: combinations of various equality, responsibility and capability specifications. The current Fit for 55 target for the country is -50%.

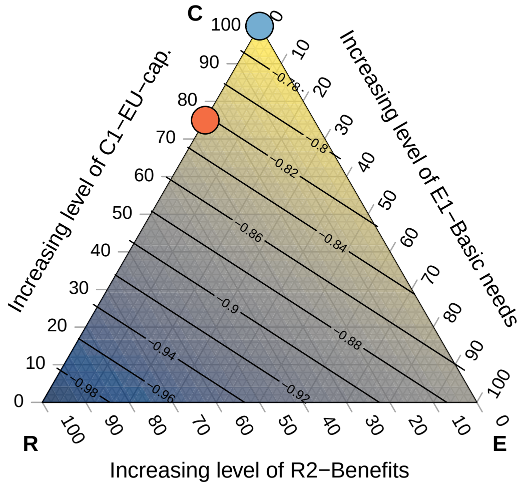

(a) R2-Benefits

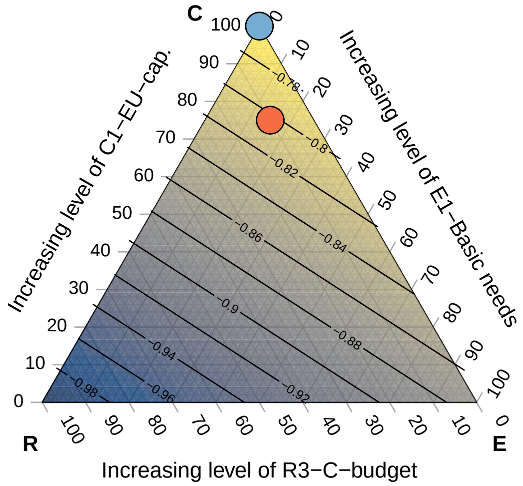

(b) R3-C-budget

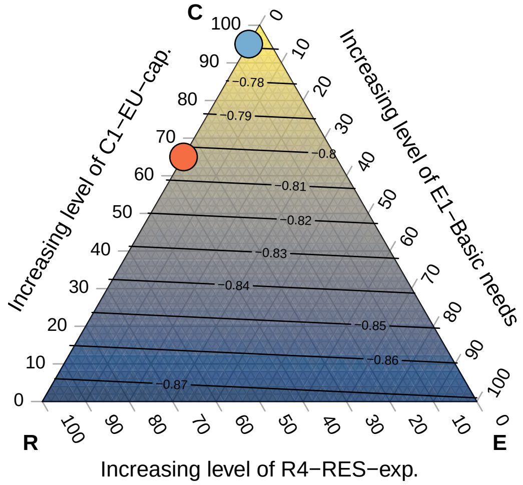

(c) R4-RES-expansion

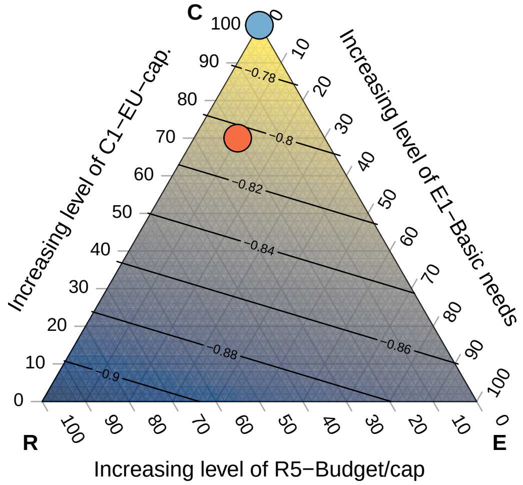

(d) R5-Budget/cap

Supplementary Figure 39. Ternary charts for Luxembourg, cont'd

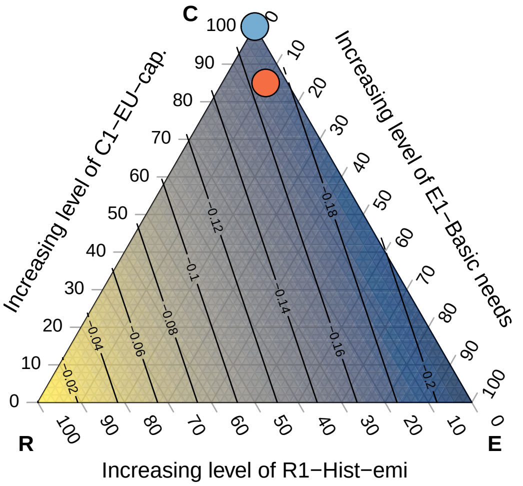

(a) Main

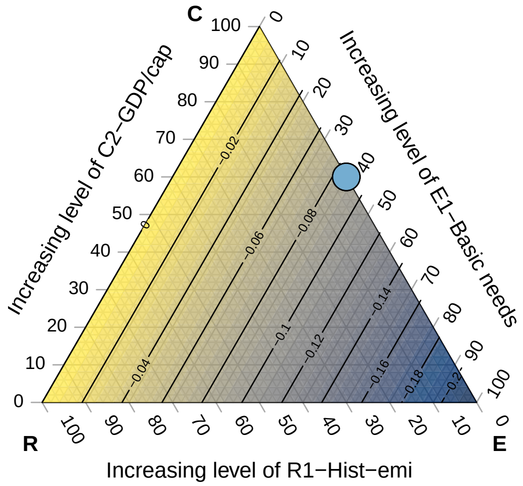

(b) C2-GDP/cap

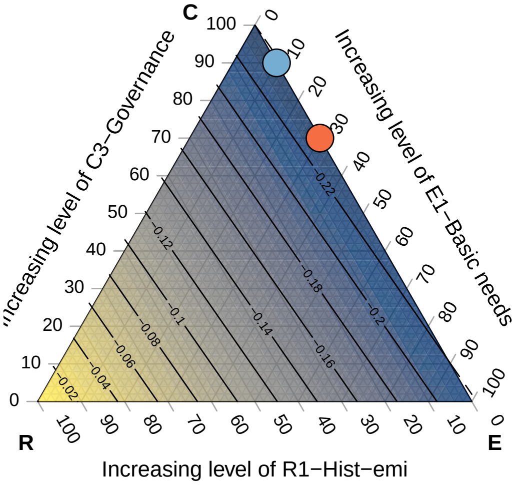

(c) C3-Governance

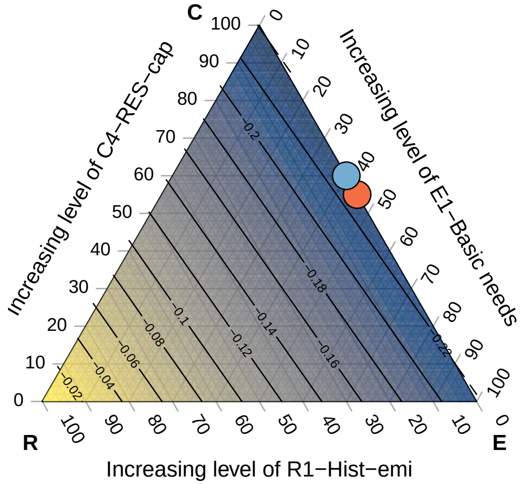

(d) C4-RES-cap.

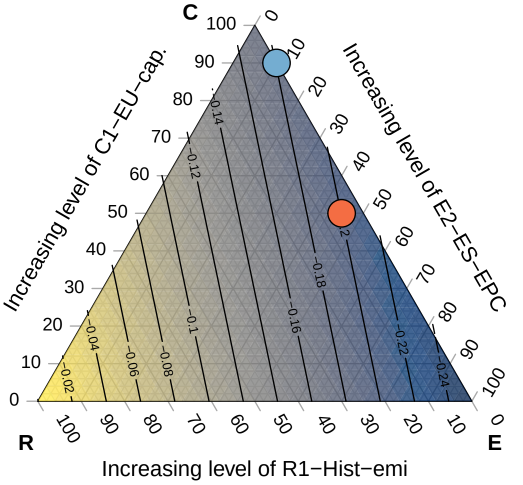

(e) E2-ES-EPC

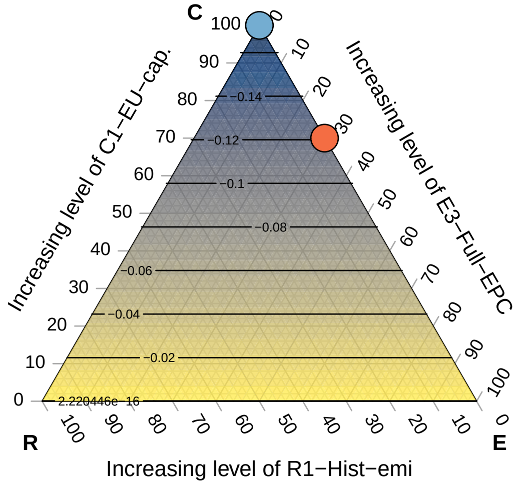

(f) E3-Full-EPC

Supplementary Figure 40. Ternary charts for Latvia: combinations of various equality, responsibility and capability specifications. The current Fit for 55 target for the country is -17%.

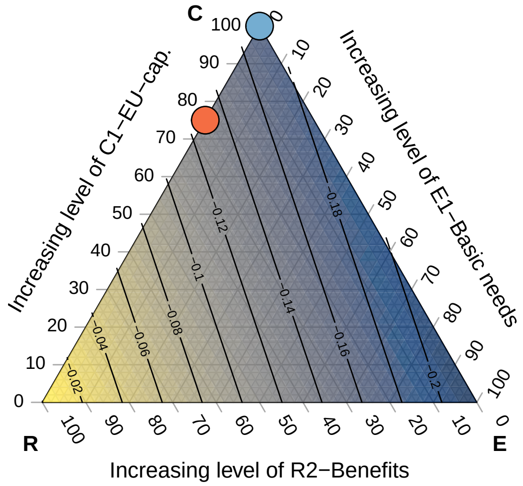

(a) R2-Benefits

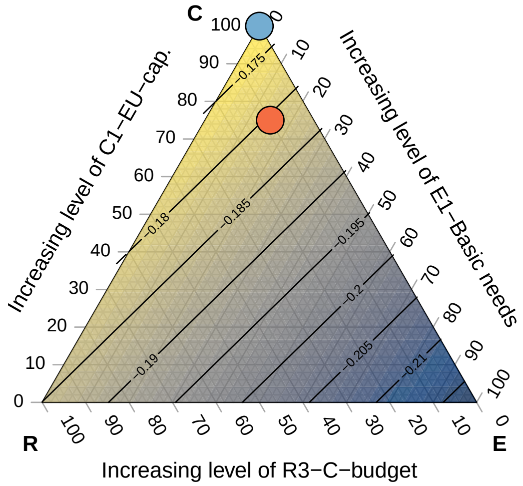

(b) R3-C-budget

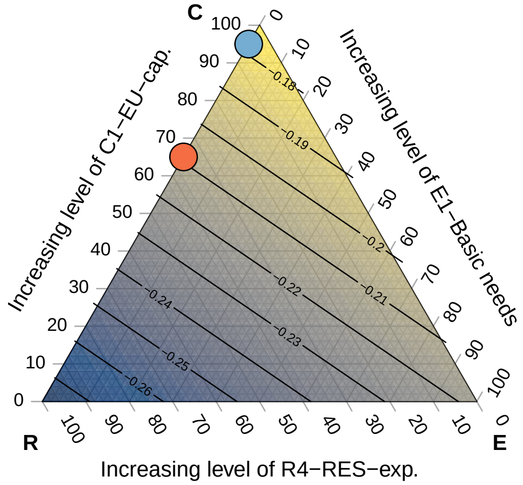

(c) R4-RES-expansion

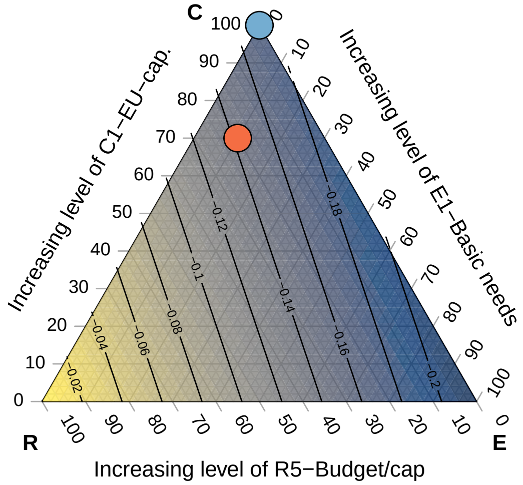

(d) R5-Budget/cap

Supplementary Figure 41. Ternary charts for Latvia, cont'd

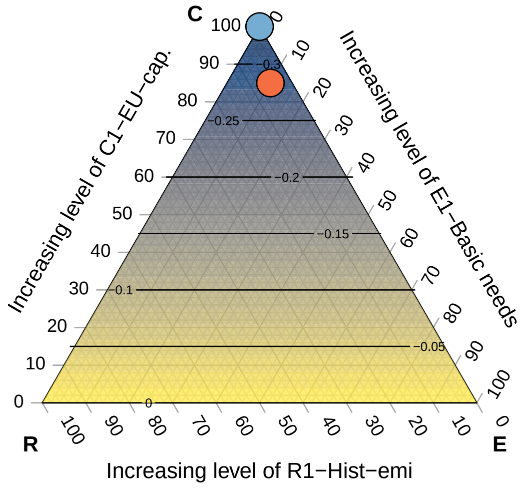

(a) Main

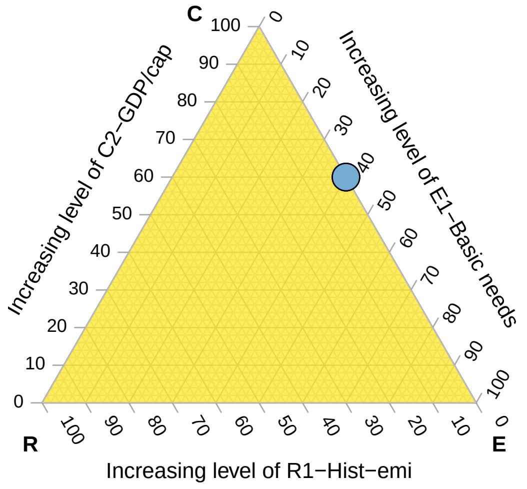

(b) C2-GDP/cap

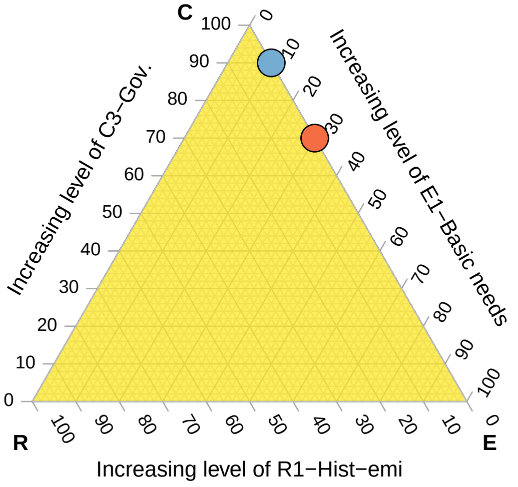

(c) C3-Governance

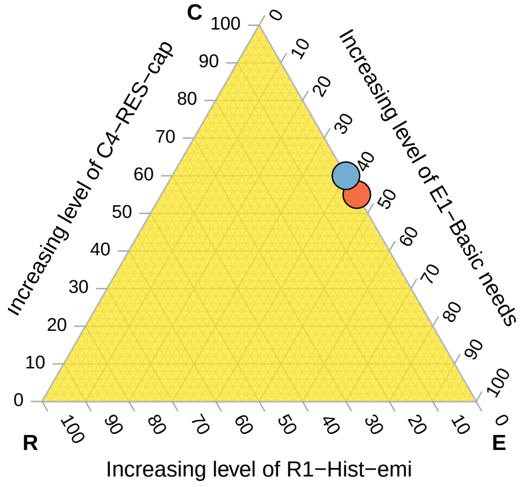

(d) C4-RES-cap.

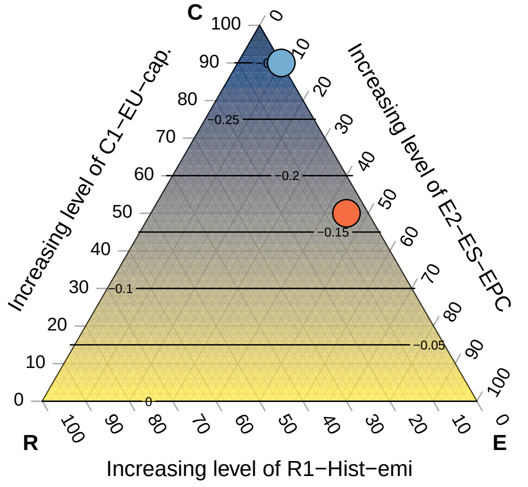

(e) E2-ES-EPC

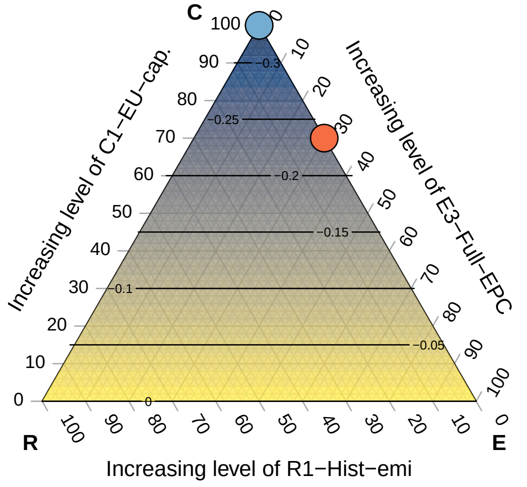

(f) E3-Full-EPC

Supplementary Figure 42. Ternary charts for Malta: combinations of various equality, responsibility and capability specifications. The current Fit for 55 target for the country is -19%. For panels (b), (c) and (d) all three interpretations result in the same reduction target.

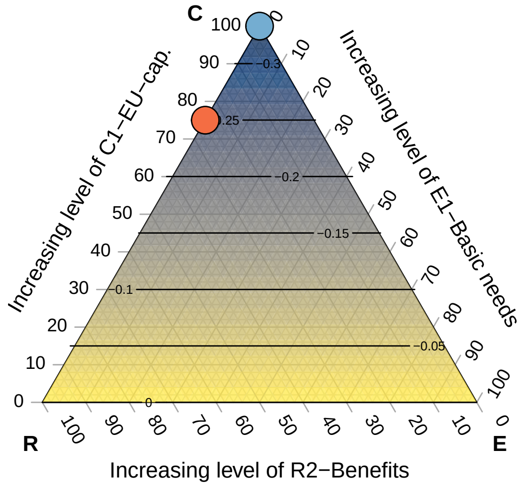

(a) R2-Benefits

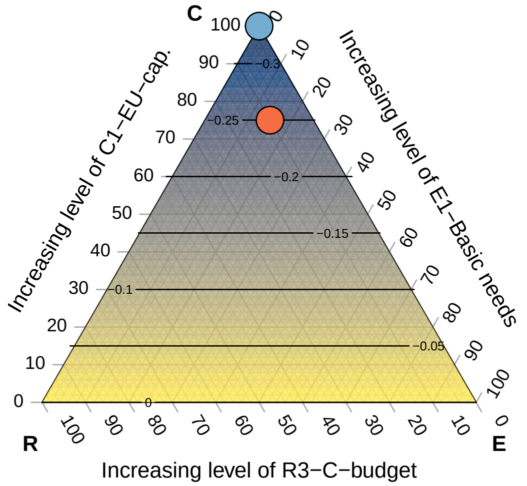

(b) R3-C-budget

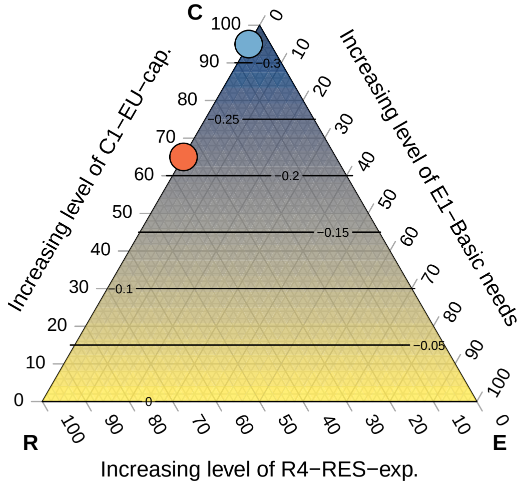

(c) R4-RES-expansion

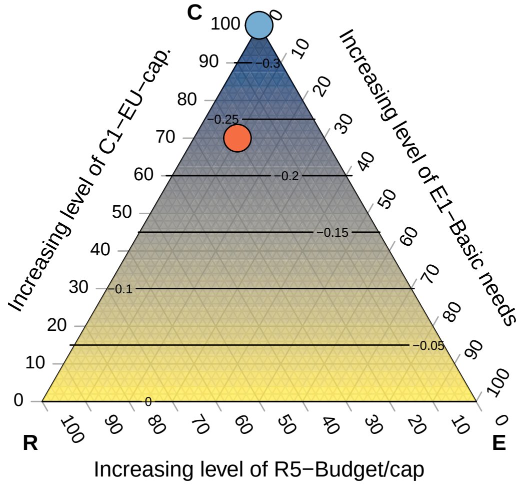

(d) R5-Budget/cap

Supplementary Figure 43. Ternary charts for Malta, cont'd

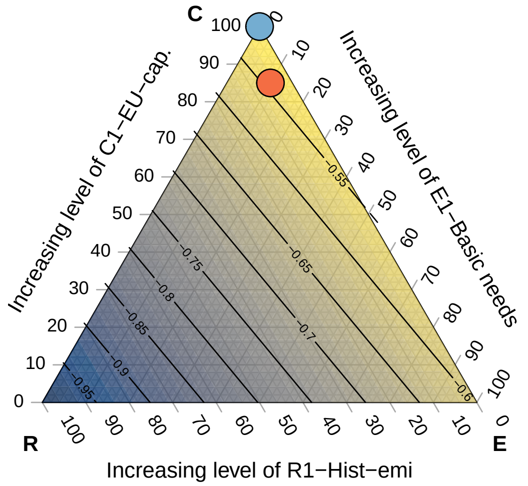

(a) Main

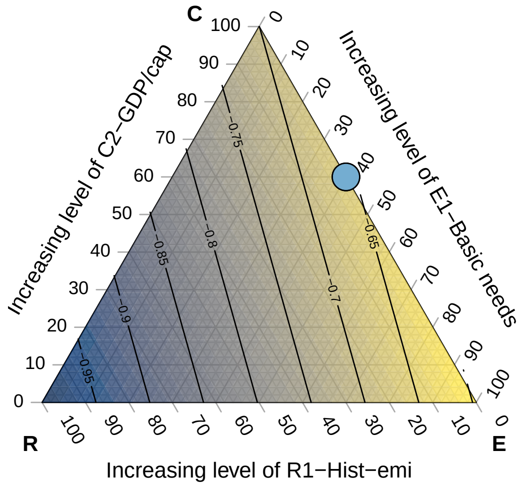

(b) C2-GDP/cap

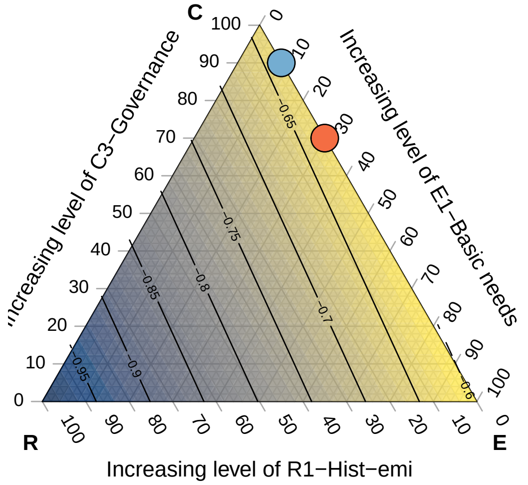

(c) C3-Governance

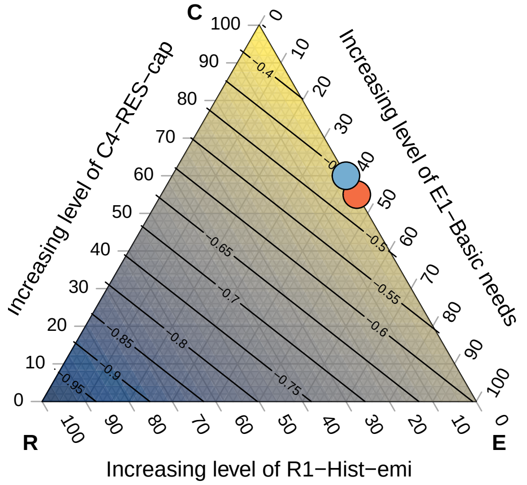

(d) C4-RES-cap.

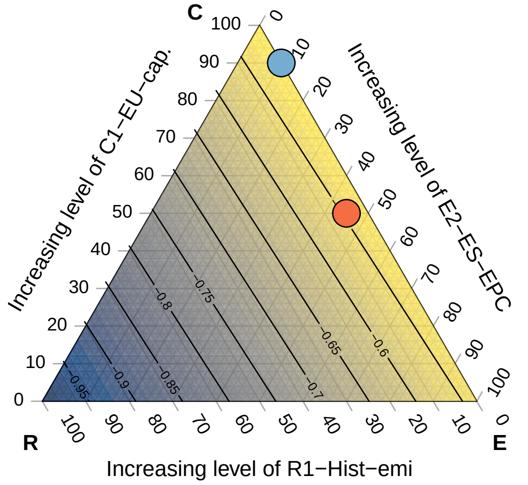

(e) E2-ES-EPC

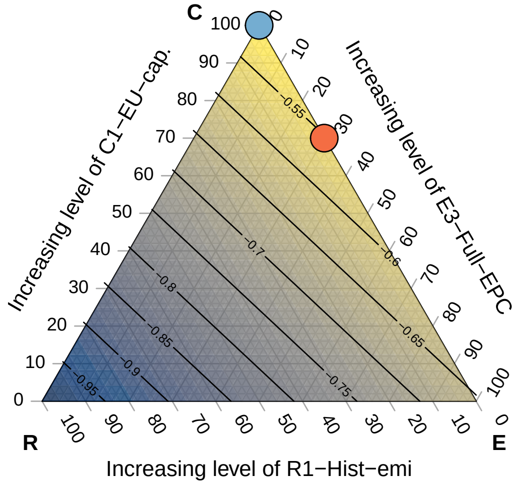

(f) E3-Full-EPC

Supplementary Figure 44. Ternary charts for the Netherlands: combinations of various equality, responsibility and capability specifications. The current Fit for 55 target for the country is -48%.

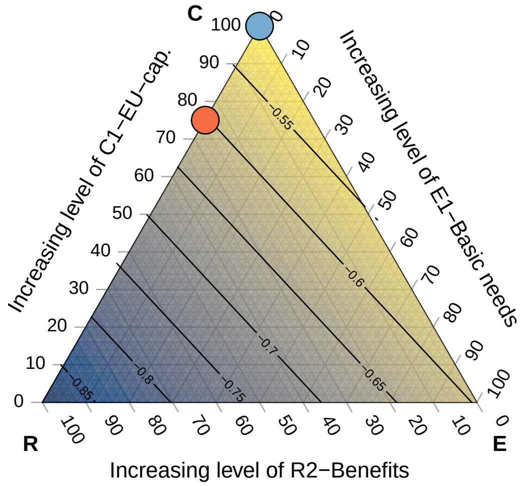

(a) R2-Benefits

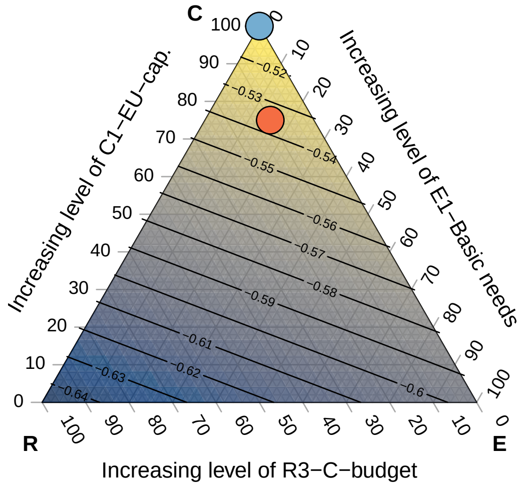

(b) R3-C-budget

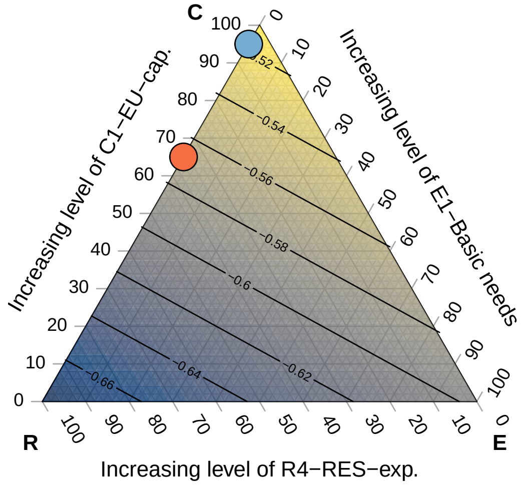

(c) R4-RES-expansion

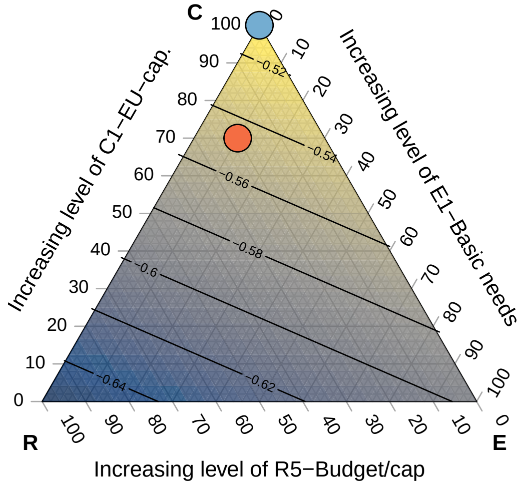

(d) R5-Budget/cap

Supplementary Figure 45. Ternary charts for the Netherlands, cont'd

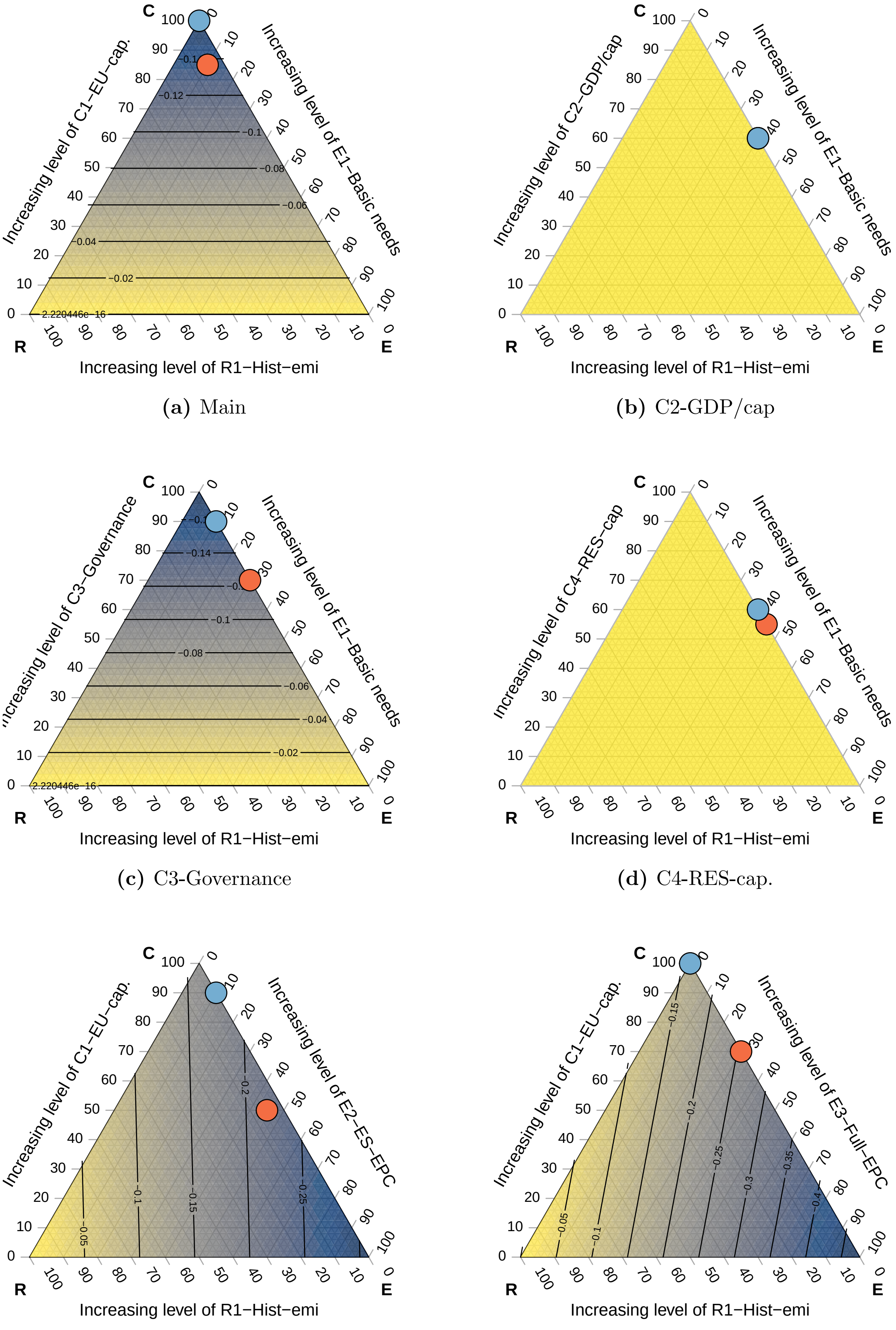

(a) Main **(b)** C2-GDP/cap

(c) C3-Governance **(d)** C4-RES-cap.

(e) E2-ES-EPC **(f)** E3-Full-EPC

Supplementary Figure 46. Ternary charts for Poland: combinations of various equality, responsibility and capability specifications. The current Fit for 55 target for the country is -17.7%. For panels (b) and (d) all three interpretations result in the same reduction target.

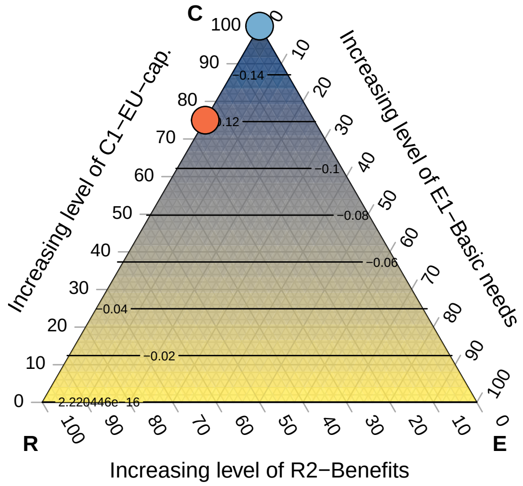

(a) R2-Benefits

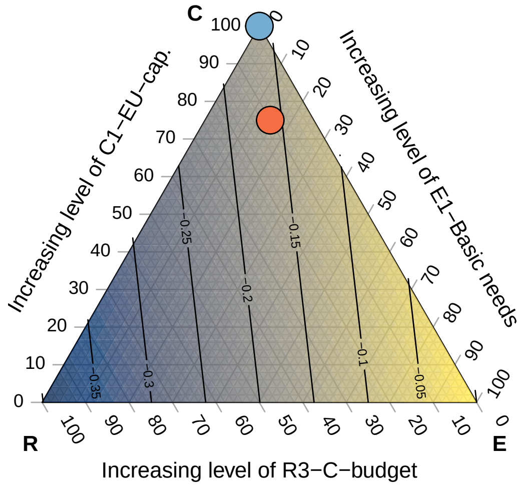

(b) R3-C-budget

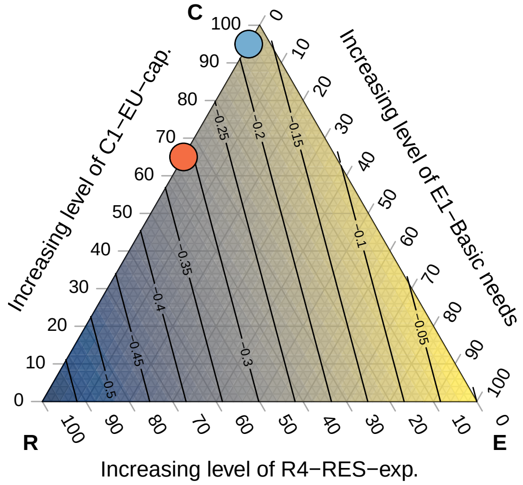

(c) R4-RES-expansion

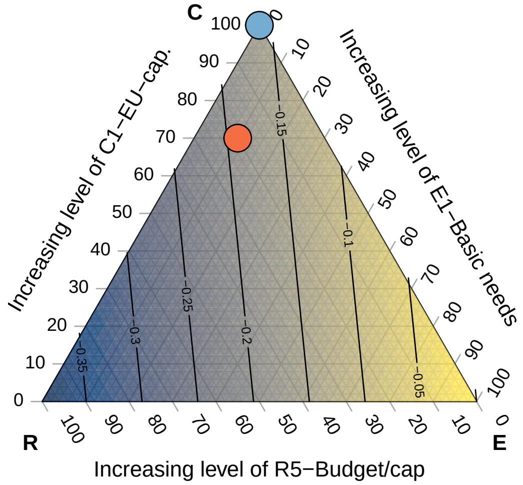

(d) R5-Budget/cap

Supplementary Figure 47. Ternary charts for Poland, cont'd

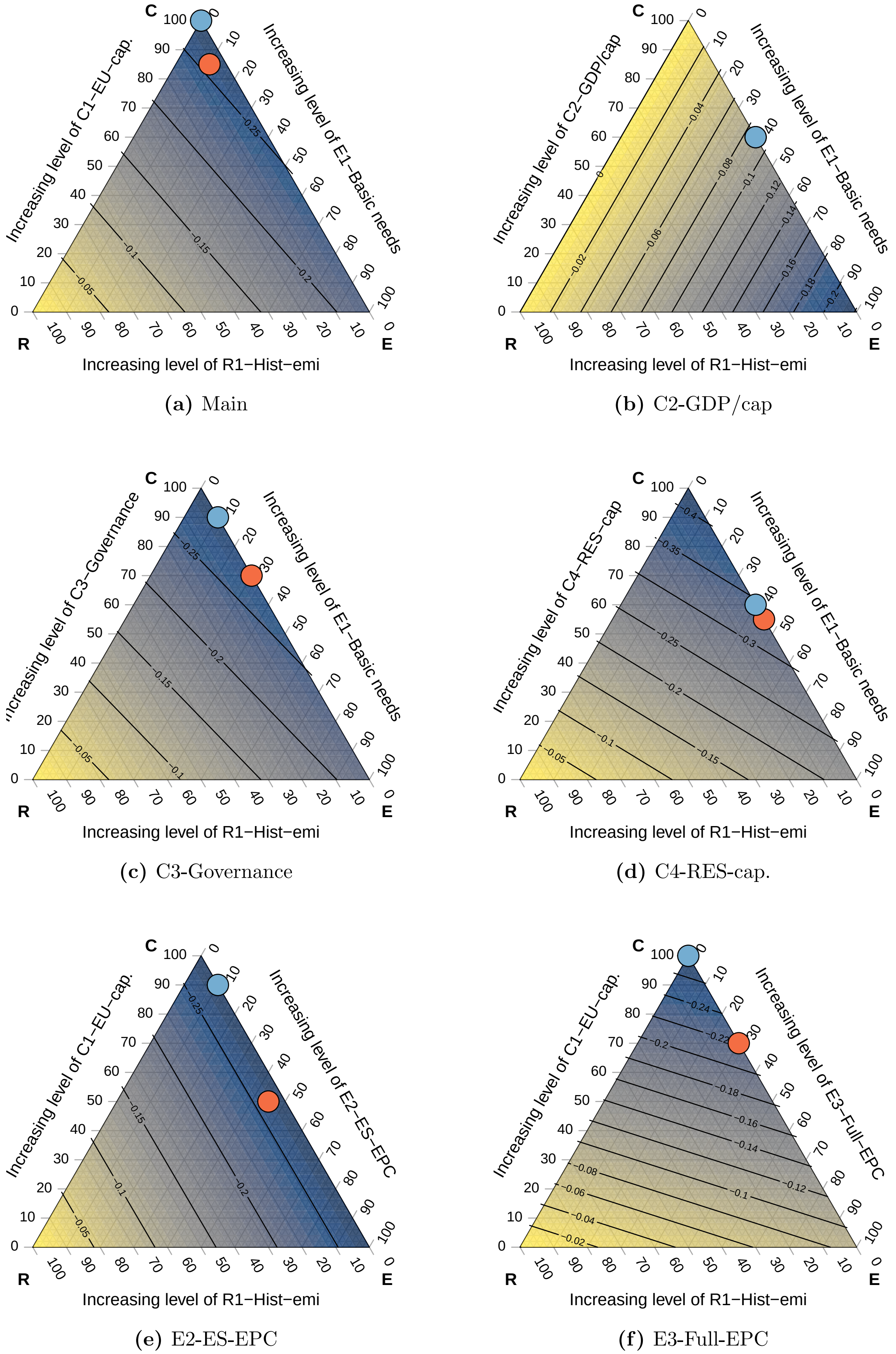

(a) Main

(b) C2-GDP/cap

(c) C3-Governance

(d) C4-RES-cap.

(e) E2-ES-EPC

(f) E3-Full-EPC

Supplementary Figure 48. Ternary charts for Portugal: combinations of various equality, responsibility and capability specifications. The current Fit for 55 target for the country is -28.7%.

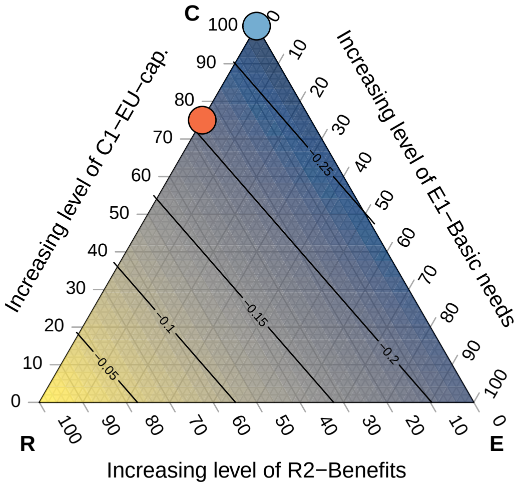

(a) R2-Benefits

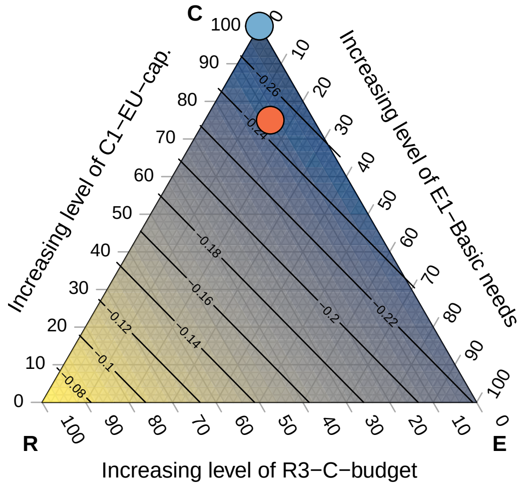

(b) R3-C-budget

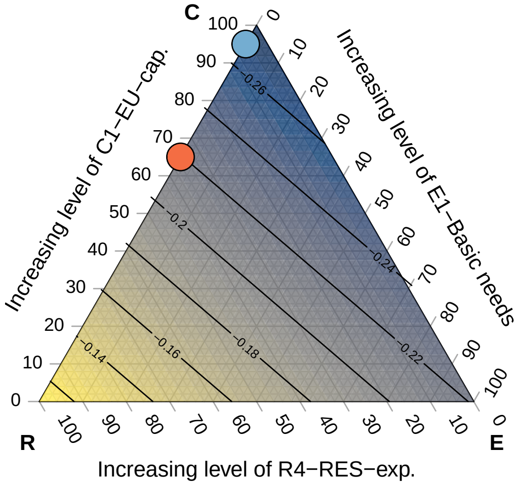

(c) R4-RES-expansion

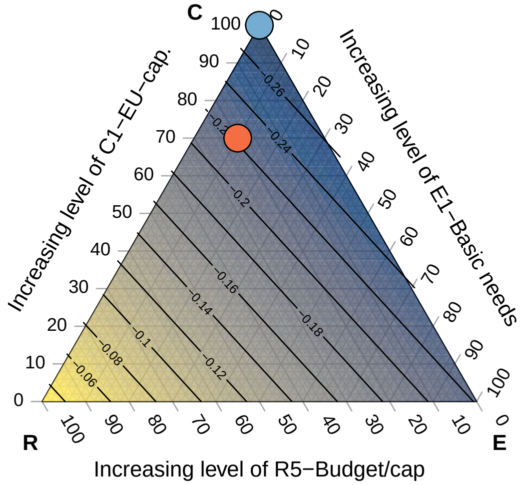

(d) R5-Budget/cap

Supplementary Figure 49. Ternary charts for Portugal, cont'd

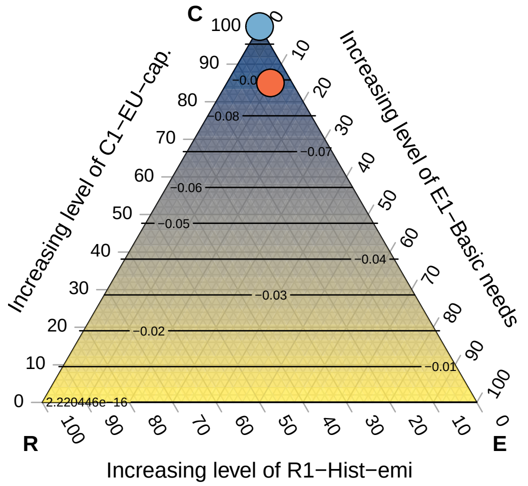

(a) Main

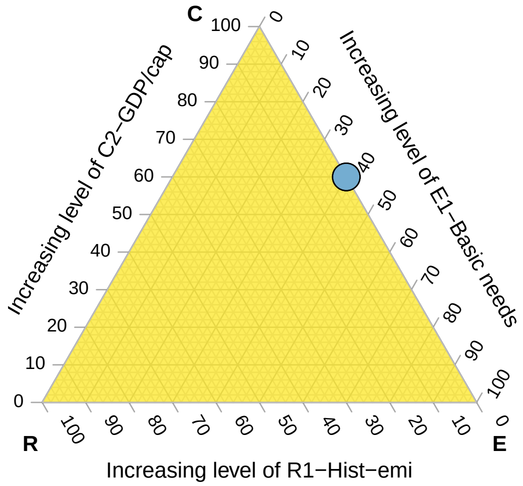

(b) C2-GDP/cap

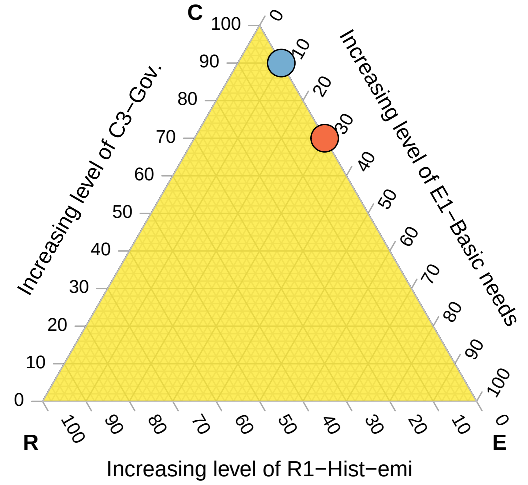

(c) C3-Governance

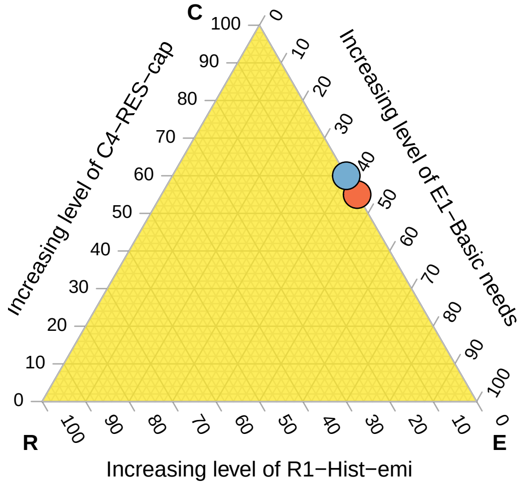

(d) C4-RES-cap.

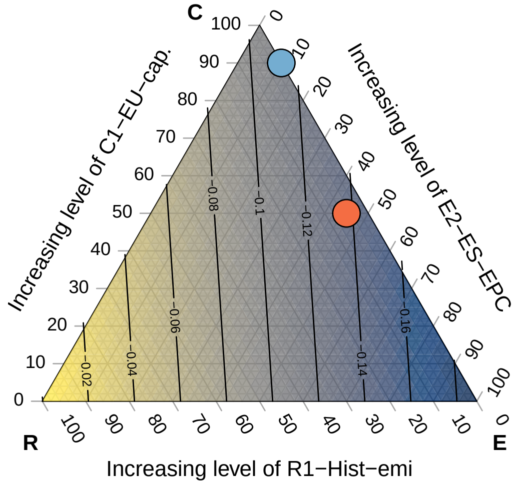

(e) E2-ES-EPC

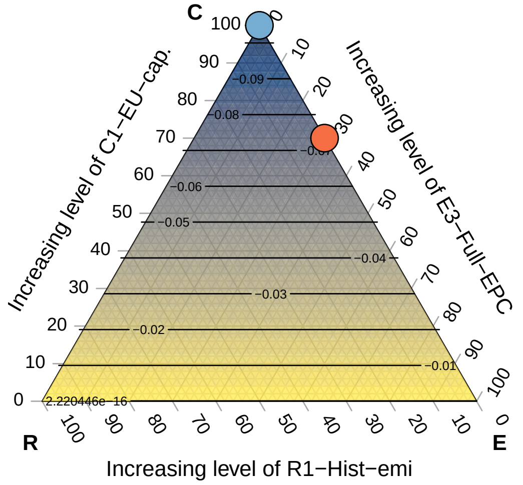

(f) E3-Full-EPC

Supplementary Figure 50. Ternary charts for Romania: combinations of various equality, responsibility and capability specifications. The current Fit for 55 target for the country is -12.7%. For panels (b), (c) and (d) all three interpretations result in the same reduction target.

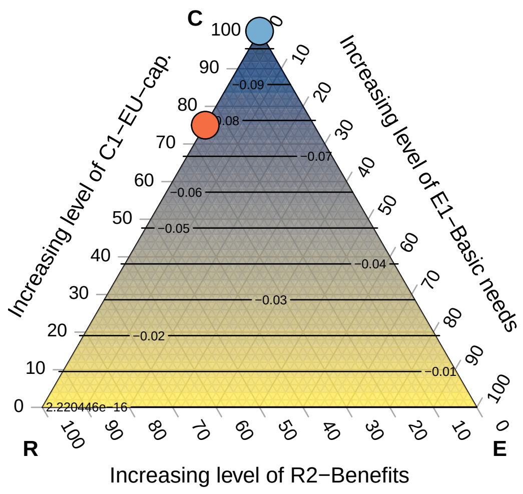

(a) R2-Benefits

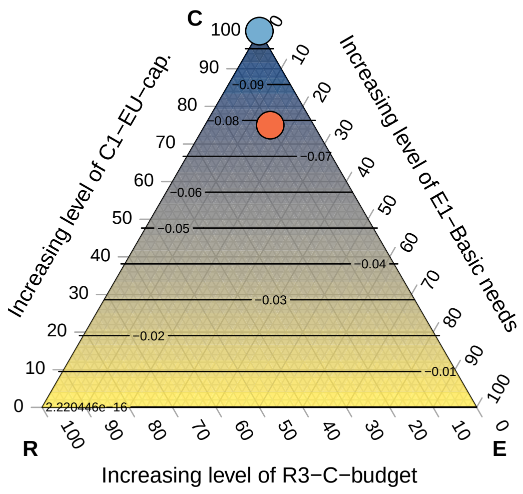

(b) R3-C-budget

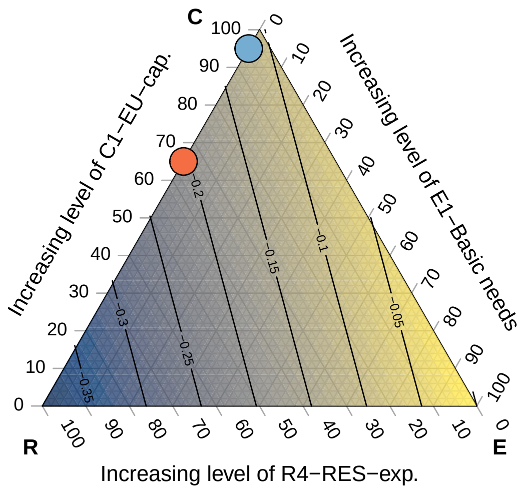

(c) R4-RES-expansion

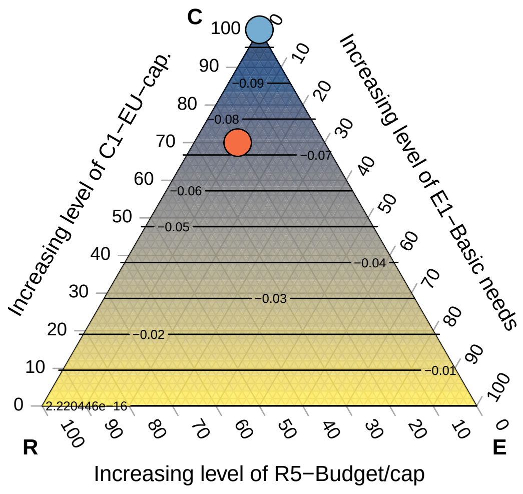

(d) R5-Budget/cap

Supplementary Figure 51. Ternary charts for Romania, cont'd

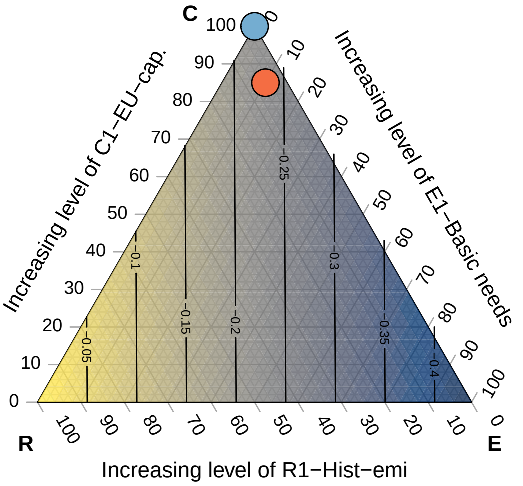

(a) Main

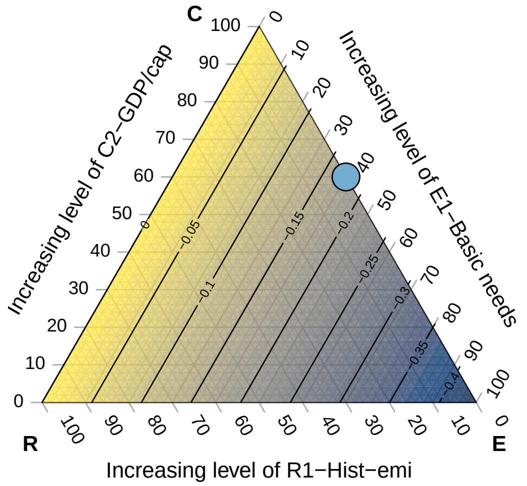

(b) C2-GDP/cap

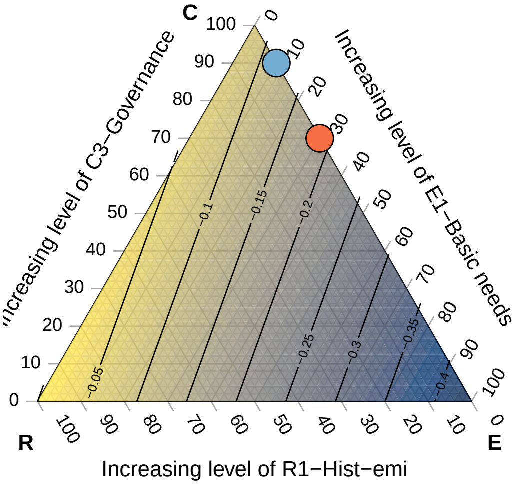

(c) C3-Governance

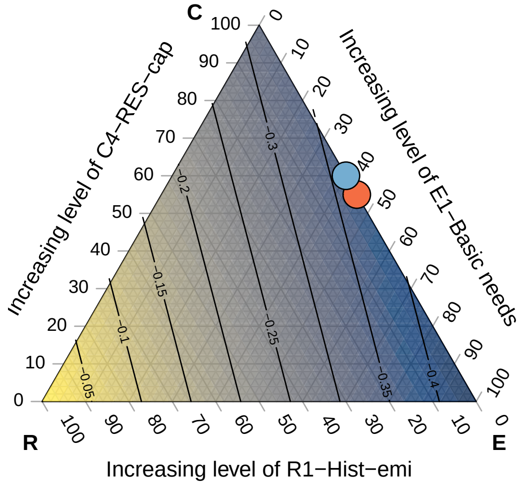

(d) C4-RES-cap.

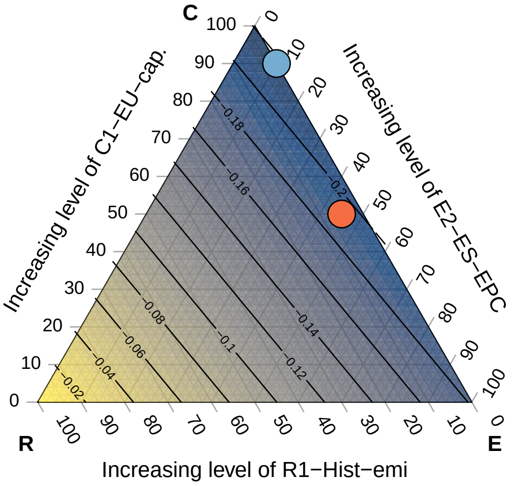

(e) E2-ES-EPC

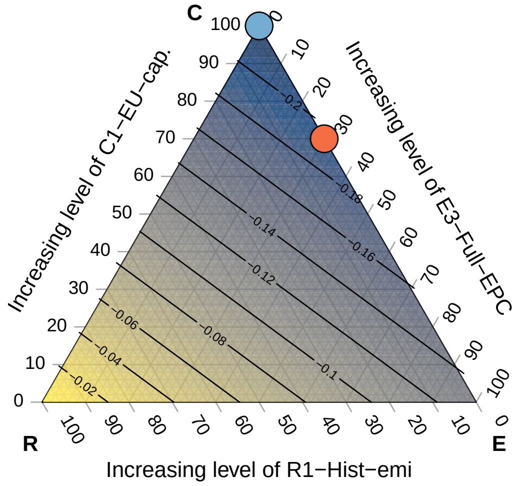

(f) E3-Full-EPC

Supplementary Figure 52. Ternary charts for Slovakia: combinations of various equality, responsibility and capability specifications. The current Fit for 55 target for the country is -22.7%.

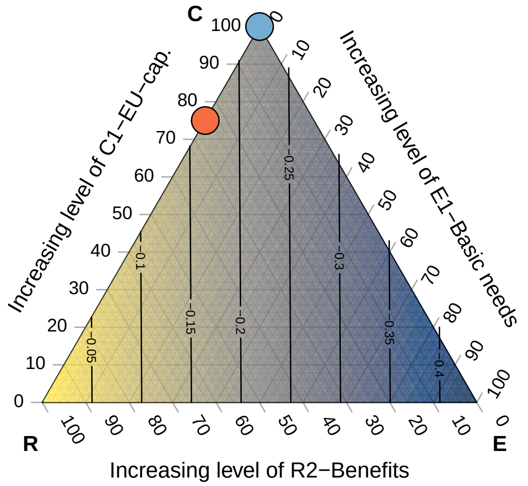

(a) R2-Benefits

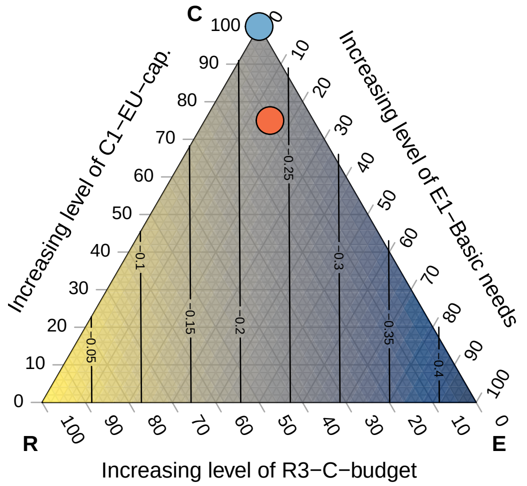

(b) R3-C-budget

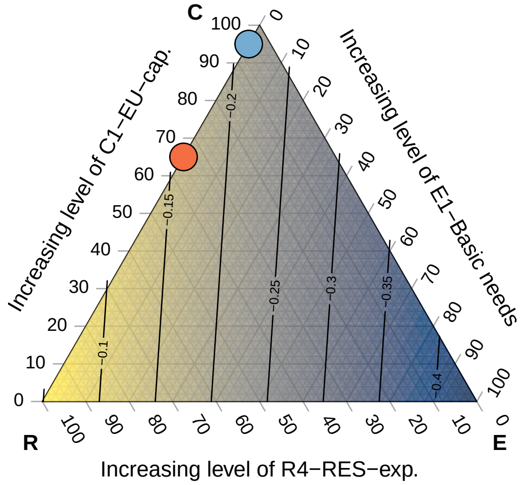

(c) R4-RES-expansion

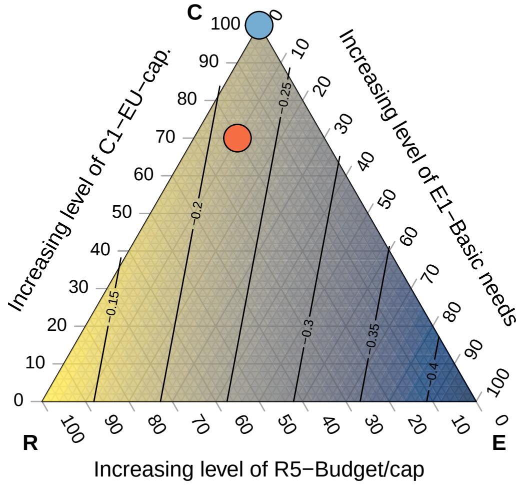

(d) R5-Budget/cap

Supplementary Figure 53. Ternary charts for Slovakia, cont'd

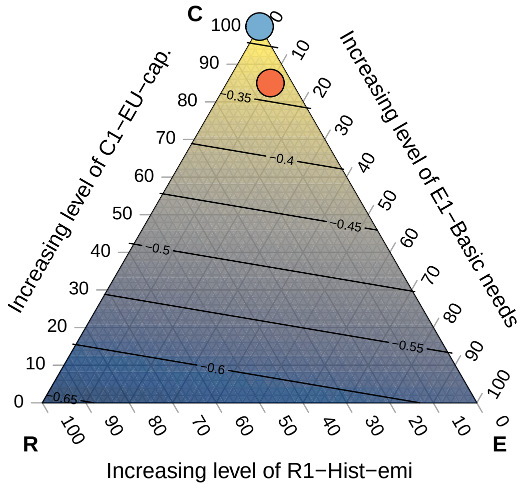

(a) Main

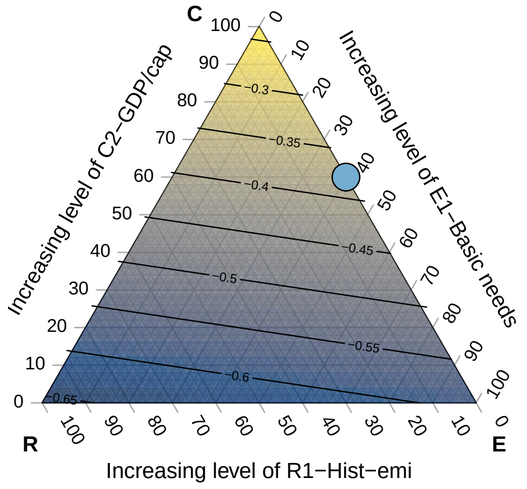

(b) C2-GDP/cap

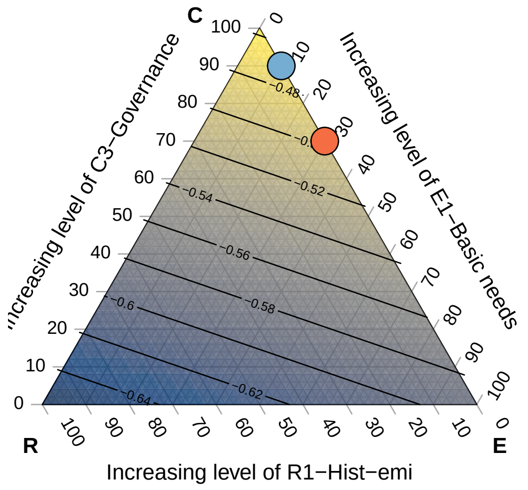

(c) C3-Governance

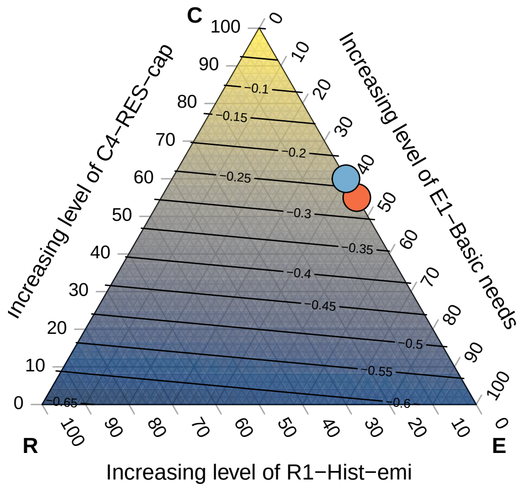

(d) C4-RES-cap.

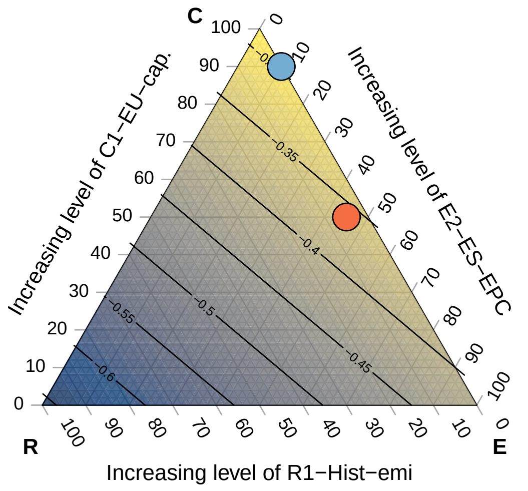

(e) E2-ES-EPC

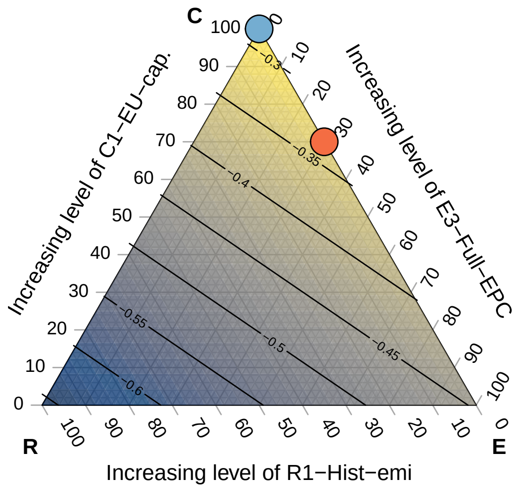

(f) E3-Full-EPC

Supplementary Figure 54. Ternary charts for Slovenia: combinations of various equality, responsibility and capability specifications. The current Fit for 55 target for the country is -27%.

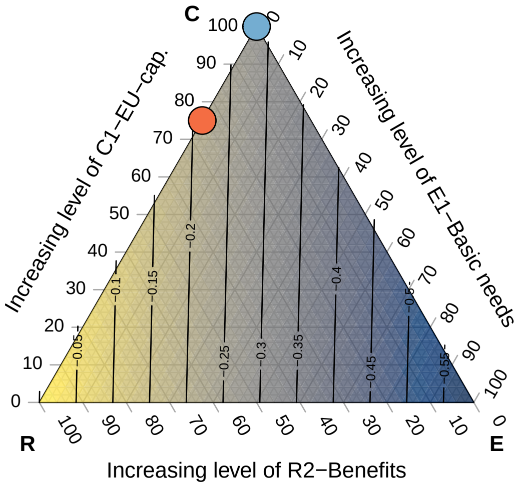

(a) R2-Benefits

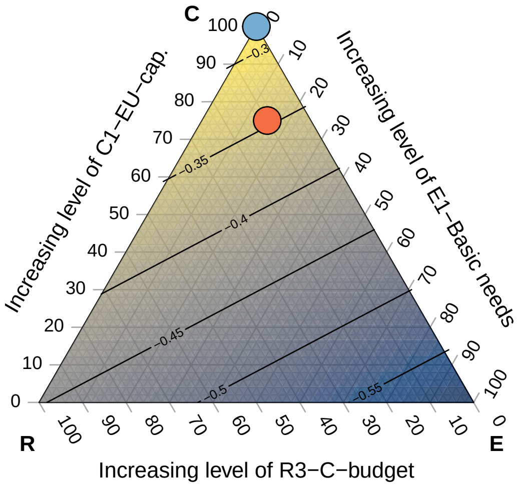

(b) R3-C-budget

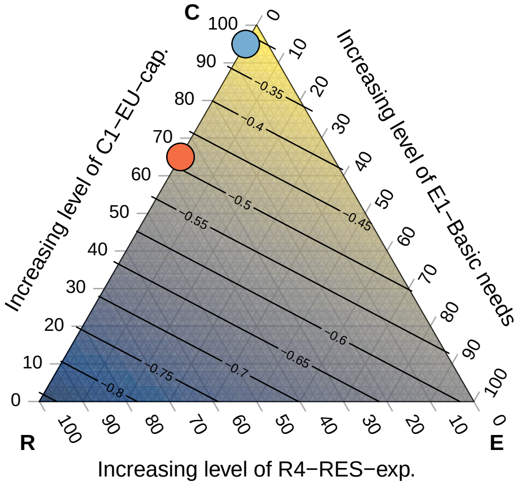

(c) R4-RES-expansion

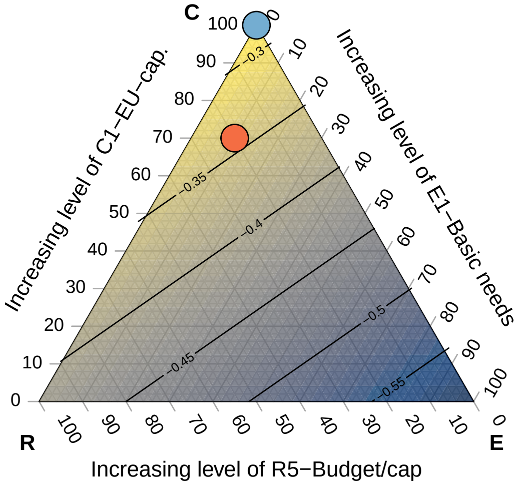

(d) R5-Budget/cap

Supplementary Figure 55. Ternary charts for Slovenia, cont'd

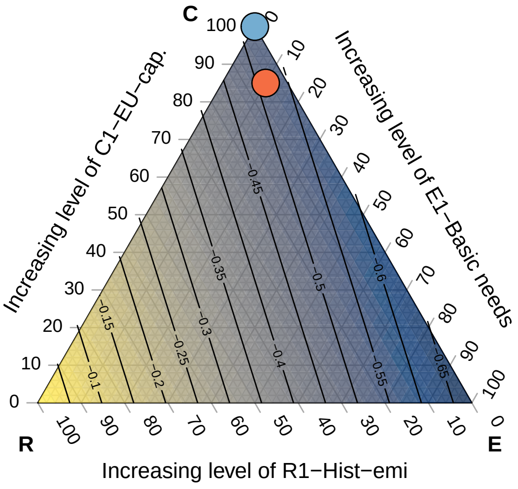

(a) Main

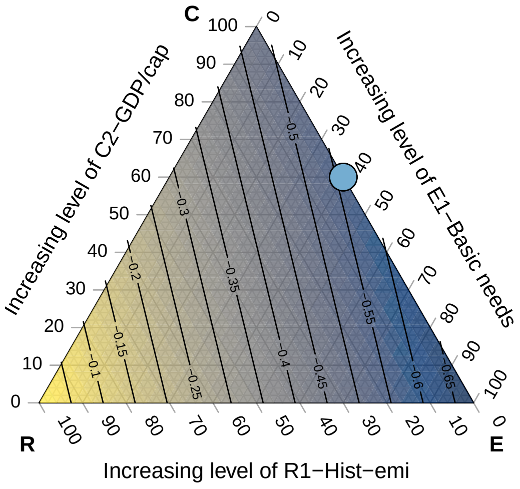

(b) C2-GDP/cap

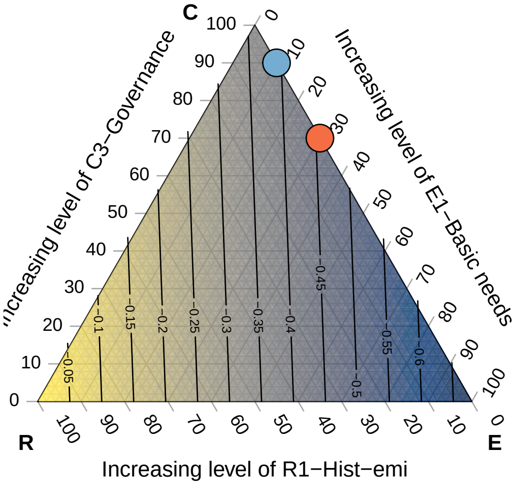

(c) C3-Governance

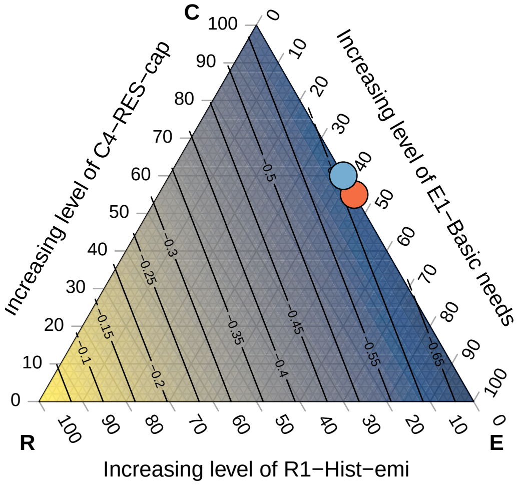

(d) C4-RES-cap.

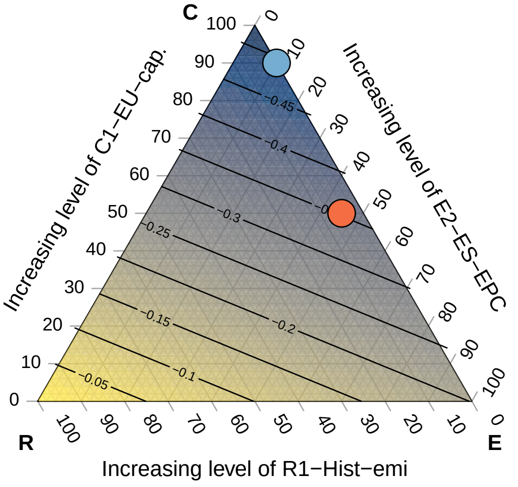

(e) E2-ES-EPC

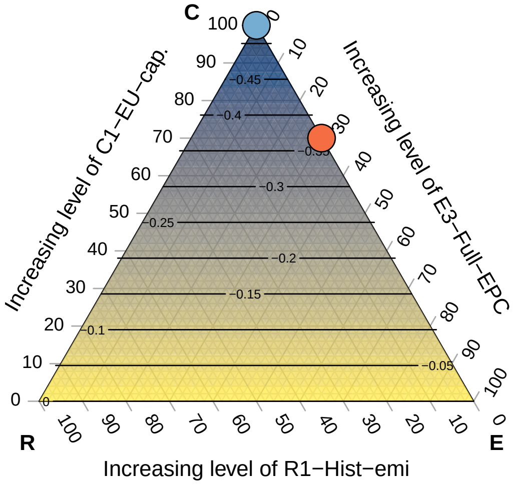

(f) E3-Full-EPC

Supplementary Figure 56. Ternary charts for Sweden: combinations of various equality, responsibility and capability specifications. The current Fit for 55 target for the country is -50%.

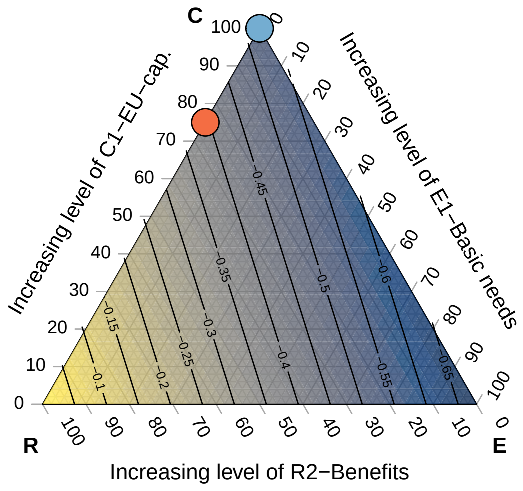

(a) R2-Benefits

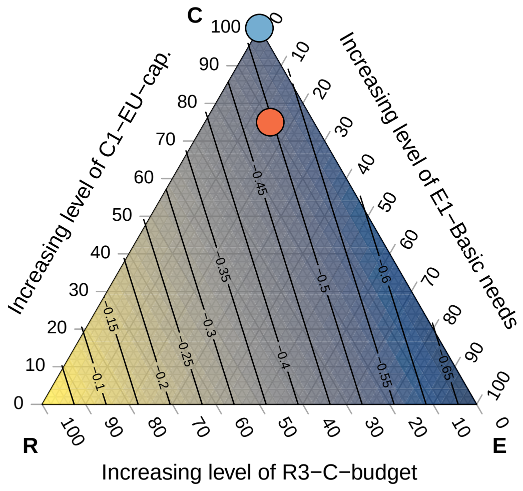

(b) R3-C-budget

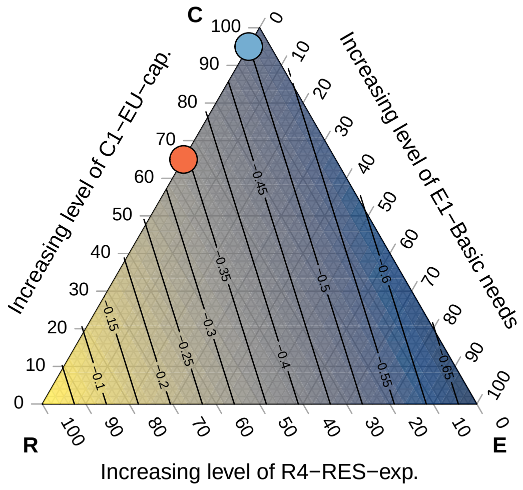

(c) R4-RES-expansion

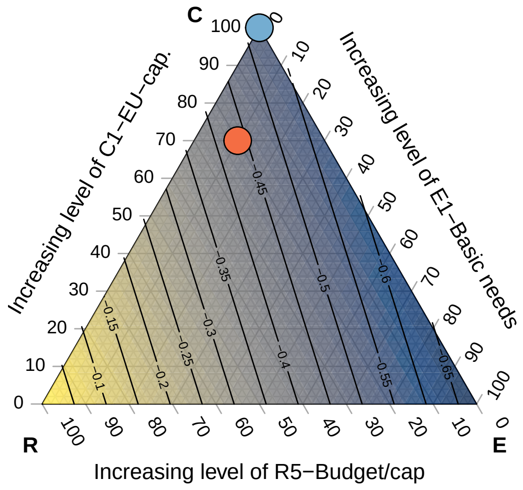

(d) R5-Budget/cap

Supplementary Figure 57. Ternary charts for Sweden, cont'd

References

1. Ringius. Differentiation, Leaders, and Fairness: Negotiating Climate Commitments in the European Community. *Int. Negot.* **4**, 133–166 (1999).

2. Sijm, J. P. M., Berk, M. M., den Elzen, M. G. J. & van den Wijngaart, R. A. *Options for post-2012 EU burden sharing and EU ETS allocation*. 80 (2007).

3. Cingolani, L. *The State of State Capacity : a review of concepts, evidence and measures. MERIT Working Papers* https://ideas.repec.org/p/unm/unumer/2013053.html (2013).

4. Savoia, A. & Sen, K. Measurement, Evolution, Determinants, and Consequences of State Capacity: A Review of Recent Research. *J. Econ. Surv.* **29**, 441–458 (2015).

5. Hanson, J. K. & Sigman, R. Leviathan's Latent Dimensions: Measuring State Capacity for Comparative Political Research. *J. Polit.* **83**, 1495–1510 (2021).

6. Hendrix, C. S. Measuring state capacity: Theoretical and empirical implications for the study of civil conflict. *J. Peace Res.* **47**, 273–285 (2010).

7. Kaufmann, D., Kraay, A. & Mastruzzi, M. *The Worldwide Governance Indicators: Methodology and Analytical Issues*. https://papers.ssrn.com/abstract=1682130 (2010).

8. Teorell, J. *et al. The Quality of Government Standard Dataset, version Jan21*. https://papers.ssrn.com/abstract=3786283 (2021) doi:10.2139/ssrn.3786283.
